# Pan-human consensus genome significantly improves the accuracy of RNA-seq analyses

**DOI:** 10.1101/gr.275613.121

**Published:** 2022-04

**Authors:** Benjamin Kaminow, Sara Ballouz, Jesse Gillis, Alexander Dobin

**Affiliations:** 1 Cold Spring Harbor Laboratory, Cold Spring Harbor, New York 11724, USA;; 2 Tri-Institutional Ph.D. Program in Computational Biology and Medicine, Weill Cornell Graduate School of Medical Sciences, New York, New York 10065, USA;; 3 Garvan-Weizmann Centre for Cellular Genomics, Garvan Institute of Medical Research, Darlinghurst, New South Wales 2010, Australia;; 4 School of Medical Sciences, University of New South Wales, Sydney, New South Wales 2052, Australia

## Abstract

The Human Reference Genome serves as the foundation for modern genomic analyses. However, in its present form, it does not adequately represent the vast genetic diversity of the human population. In this study, we explored the consensus genome as a potential successor of the current reference genome and assessed its effect on the accuracy of RNA-seq read alignment. To find the best haploid genome representation, we constructed consensus genomes at the pan-human, superpopulation, and population levels, using variant information from The 1000 Genomes Project Consortium. Using personal haploid genomes as the ground truth, we compared mapping errors for real RNA-seq reads aligned to the consensus genomes versus the reference genome. For reads overlapping homozygous variants, we found that the mapping error decreased by a factor of approximately two to three when the reference was replaced with the pan-human consensus genome. We also found that using more population-specific consensuses resulted in little to no increase over using the pan-human consensus, suggesting a limit in the utility of incorporating a more specific genomic variation. Replacing the reference with consensus genomes impacts functional analyses, such as differential expressions of isoforms, genes, and splice junctions.

In 2003, 15 years of work culminated with the International Human Genome Sequencing Consortium publishing the first finished version of the Human Reference Genome (International Human Genome Sequencing Consortium 2004; https://www.genome.gov/human-genome-project/Completion-FAQ). Despite the utility and continuous improvements over the years, it is still not without flaws, primarily the lack of variation information. Around 93% of the current GRCh38 assembly is composed of DNA from just 11 individuals (International Human Genome Sequencing Consortium 2001; https://www.ncbi.nlm.nih.gov/grc/help/faq/). Because such a large portion of the reference comes from such a small pool of individuals, it does not adequately represent the vast diversity present in the human population (Chen and Butte 2010; Rosenfeld et al. 2012; Sherman et al. 2019). To explore and capture human diversity, researchers have continued sequencing thousands of genomes. The first of such projects, The 1000 Genomes Project Consortium, sequenced 2504 individuals across 26 populations. Overall, it was estimated that approximately 3000 genomes would be necessary to capture the most common variants (Ionita-Laza et al. 2009); however, structural variation present in the human population has challenged this (Berlin et al. 2015). One particularly glaring example was shown in a recent construction of an African pan-genome, which contained almost 300 million bases of DNA not seen in GRCh38 (Sherman et al. 2019). This lack of variation information negatively affects all kinds of genomic analyses that use the reference, such as disease studies and GWAS analyses (Chen and Butte 2010; Rosenfeld et al. 2012; Stevenson et al. 2013; Buchkovich et al. 2015; Castel et al. 2015; Sherman et al. 2019). However, despite the ubiquity of RNA-seq alignment and quantification, the improvements in mapping from using a more diverse reference have not been shown.

Although graph genomes are theoretically capable of encapsulating all observed variation information (Church et al. 2015; Paten et al. 2017; Garrison et al. 2018; Valenzuela et al. 2018; Rakocevic et al. 2019; Sirén et al. 2021), it remains challenging to use these tools for large-scale expression analyses such as in RNA-seq quantification. In prior work, we proposed using a consensus genome to inherently capture common variation while still retaining the structure and functionality of the current reference assembly (Ballouz et al. 2019). A consensus genome is a linear haploid genome that incorporates population variation information by replacing all minor alleles in the reference genome with the major allele of that variant (Fig. 1A; Balasubramanian et al. 2011; Dewey et al. 2011; Karthikeyan et al. 2017; Barbitoff et al. 2018; Pritt et al. 2018; Ballouz et al. 2019; Shukla et al. 2019). Because allele frequencies must be defined with respect to a population, a consensus genome is representative of the population used to define the major and minor alleles. Prior work has shown that using a consensus genome can have positive effects on variant calling (Karthikeyan et al. 2017; Pritt et al. 2018; Shukla et al. 2019), and the construction of population-specific consensus genomes has been a major goal of multiple projects (Cho et al. 2016; Fakhro et al. 2016; Higasa et al. 2016; Sherman et al. 2019; Takayama et al. 2021). Additionally, replacing the current reference genome with a consensus genome in existing analysis pipelines is straightforward because the consensus genome is still a linear haploid sequence.

**Figure 1.**
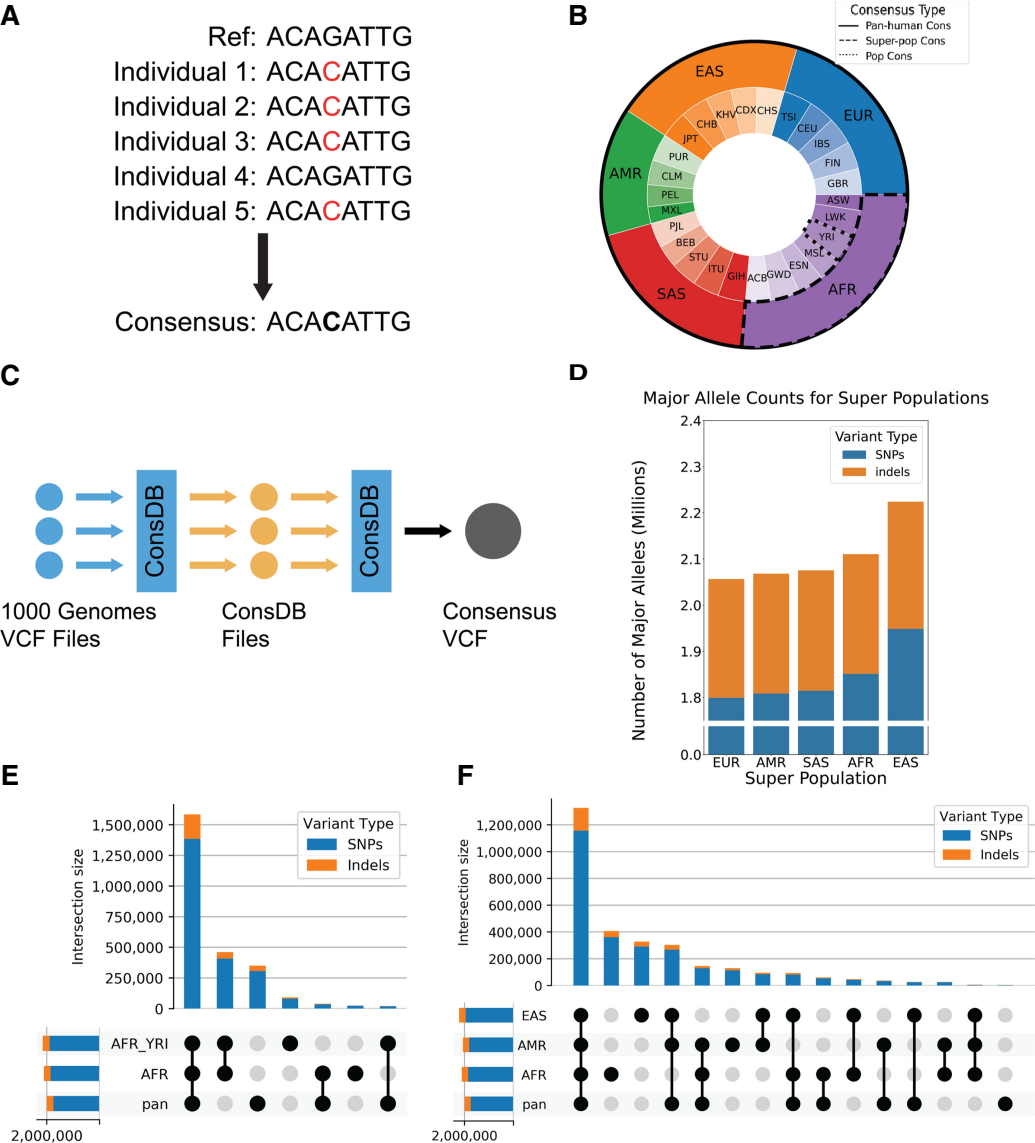
Construction of the consensus genome with major allele replacements. (*A*) Construction of a consensus genome: The minor allele in the reference is replaced by the most common (major) allele in the population. (*B*) Visual representation of the individuals used to construct consensus genomes of varying population specificity. (*C*) ConsDB workflow. (*D*) Number of major alleles for each population consensus genome that were replaced in the reference. (*E*) Number of SNPs and indels shared between different combinations of the pan-human, superpopulation, and population consensus genomes for the African population. The bars in the *top* bar plot show the number of SNPs and indels that are unique to the intersection of genomes indicated in the dot matrix *below*. The horizontal bars on the *bottom left* show the total number of SNPs and indels present in each genome. (*F*) Number of SNPs and indels shared between different combinations of the pan-human consensus and all three superpopulation consensus genomes. The bars in the *top* bar plot show the number of SNPs and indels that are unique to the intersection of genomes indicated in the dot matrix *below*. The horizontal bars on the *bottom left* show the total number of SNPs and indels present in each genome.

Here, we seek to answer the question of which linear reference representation is best for RNA-seq mapping and downstream analyses. We considered several consensus genomes, built by replacing all minor alleles in the reference with the major alleles at different population levels: pan-human, superpopulation, and population. To work with consensus genomes, we developed ConsDB to construct pan-human and population-level consensuses, as well as STAR-consensus to streamline RNA-seq mapping to consensus genomes. We defined the ground truth by mapping the individuals’ RNA-seq reads to their personal haploid genomes and evaluated the mapping accuracy improvements arising from replacing the GRCh38 reference with the pan-human consensus, superpopulation, or population consensus genomes. We found that for all individuals, the pan-human consensus decreased the mapping error from the reference by approximately two- to threefold, whereas the superpopulation and population consensuses did not perform significantly better than the pan-human consensus. To assess the functional impact, we measured errors in splice junction expression quantification for different genome representations with respect to the ground truth of the personal genome. We again found that the pan-human consensus offers an improvement over the reference, with about five times as many splice junctions having a larger quantification error for the reference than for the pan-human consensus.

## Results

### Pan-human consensus captures the majority of population deviation from the reference

The construction of consensus genomes requires population allele frequency (AF) information. Currently, several databases contain this information (Sherry et al. 2001; Church et al. 2015; The 1000 Genomes Project Consortium 2015; Karczewski et al. 2020). In this study, we used The 1000 Genomes Project Consortium database, which was established to discover and catalog human genome variant information (The 1000 Genomes Project Consortium 2015; Clarke et al. 2017). To avoid population bias, the individuals genotyped in The 1000 Genomes Project Consortium were selected to create an even population distribution across 26 populations, which are grouped into five superpopulations (Fig. 1B; The 1000 Genomes Project Consortium 2015). This balance between the different populations means that The 1000 Genomes Project Consortium database is well suited for creating a draft pan-human consensus genome, whereas other popular databases are more skewed toward specific populations and will therefore produce a biased pan-human consensus genome. Additionally, the information from The 1000 Genomes Project Consortium is publicly available through the International Genome Sample Resource (IGSR) and can be downloaded in the form of VCF files, which contain variant genotype information for all individuals (The 1000 Genomes Project Consortium 2015).

We constructed three types of consensus genomes based on the various population levels present in The 1000 Genomes Project Consortium: a pan-human consensus genome, a superpopulation consensus genome, and a population consensus genome (Fig. 1B). For the pan-human consensus, we calculated AF using genotype information from all individuals present in the database. For the superpopulation and population consensuses, we used genotype information from all individuals of a given superpopulation or population. For the eight individuals whose RNA-seq data we used in this study, we used the consensus genomes built from the superpopulation and population each individual belongs to.

To construct these consensuses, we replaced all minor alleles (alleles with a population AF < 0.5) present in the reference with the major alleles (AF > 0.5). This procedure is applied to both single-nucleotide variants and insertions/deletions. For simplicity, we omit overlapping indels. We will call these variants replaced in the reference the major allele replacements (MARs).

The release of The 1000 Genomes Project Consortium database that we used contained only biallelic variants; that is, each variant had exactly one minor allele and one major allele. Additionally, it only contained SNPs and small insertions and deletions (<50 bp), whereas large structural variants (https://www.ncbi.nlm.nih.gov/dbvar/content/overview/) were not considered in this study. Although structural variants are a large source of genomic variation, they are understudied and not sufficiently cataloged to be used in consensus genomes owing to mapping and classification difficulties (Mahmoud et al. 2019).

To facilitate working with the large VCF files of The 1000 Genomes Project Consortium database, we developed ConsDB, a Python package that provides a convenient, class-based interface to work with the large number of variants contained in The 1000 Genomes Project Consortium database. It also provides the main script with several run modes to perform common tasks associated with consensus genomes, such as constructing the consensus genome VCF files used in this study. ConsDB operates using a simple workflow (Fig. 1C). The first step is downloading the database VCF files. For this study, we used The 1000 Genomes Project Consortium, but ConsDB is also capable of parsing gnomAD VCF files. The next step is for ConsDB to parse the database VCF files and save them in the ConsDB format. At this point, files from different databases (if multiple databases are being used) can be combined into one file per chromosome. Finally, ConsDB uses these parsed files to generate the end result: in this case, a VCF file defining a consensus genome.

One of ConsDB's main benefits is that it facilitates working with large variant databases. Although tools such as BCFtools (Danecek et al. 2021) can also construct consensus genomes from VCF files, creating a population-specific consensus requires building complex expressions for variant inclusion that may prove difficult for some users. ConsDB is designed to be easy to use and allows the construction of population-specific consensuses using just a single additional file with population information. ConsDB also exposes a powerful and easy-to-use backend that allows more advanced users to incorporate its capabilities into their own pipelines.

The personal haploid genomes were constructed using the individual genotypes from The 1000 Genomes Project Consortium database. For each individual, all homozygous variants that differ from the reference were inserted into the reference. Additionally, all heterozygous alleles were randomly chosen with a probability of 0.5 to be included or excluded. Although these haploid personal genomes are a crude approximation of the actual diploid genome, they are sufficient for comparison of mapping accuracy between haploid consensuses and the haploid reference, and thus, we used them to define the ground truth for RNA-seq mapping in this study.

Figure 1D shows the number of minor alleles in the GRCh38 reference that must be replaced with the major alleles for each of the superpopulation consensus genomes. The European consensus is the most similar to the reference, and it still requires approximately 2.1 million SNP and indel corrections from the reference. Other superpopulation consensuses contain even larger numbers of major allele deviations from the reference, with the East Asian consensus differing most from the reference. We note that such a large number of minor alleles in the reference with respect to any population stems from its construction, which used sequences from only one individual for most of the genomic loci, and thus incorporated individual-specific low-frequency alleles.

In Figure 1E, we compute intersections of the MARs in the pan-human, African superpopulation, and Yoruban population consensus genomes. The pan-human consensus shares most of the major alleles with the superpopulation and population consensuses (about 1.5 million), whereas the latter two share about 400,000 MARs not present in the pan-human consensus. The pan-human consensus contains about 300,000 MARs not present in either superpopulation or population consensuses. Finally, the Yoruban population consensus has about 50,000 unique MARs. The intersections of MARs look similar for other populations (Supplemental Figs. S1, S2) and personal homozygous variants (Supplemental Figs. S3–S5). Figure 1F shows the intersections between the MARs for the pan-human consensus and three superpopulation consensuses. The MARs shared by all four of these genomes make up the largest group, containing about 1.2 million MARs and representing well over half of the MARs in any one genome. This group is more than three times as large as the next largest group, showing that most of the population deviation from the reference is captured in the pan-human consensus.

### Consensus genomes significantly improve RNA-seq mapping

Next, we analyzed to what extent the consensus genomes improve RNA-seq mapping accuracy. The RNA-seq reads were obtained from the Human Genome Structural Variation Consortium, which sequenced three father–mother–daughter trios from The 1000 Genomes Project Consortium (Fairley et al. 2020). One of these individuals (HG00514 from the East Asian trio) is not present in the database version used in this analysis and was excluded from our analyses.

**Figure 2.**
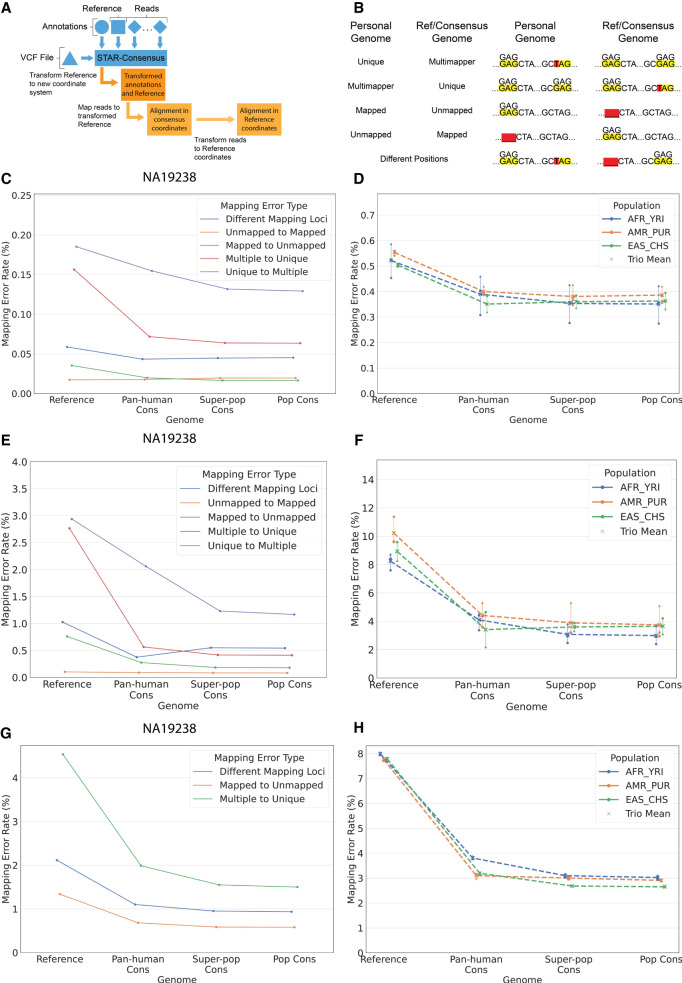
Mapping accuracy improvements owing to switching from reference to consensus genomes. (*A*) Internal workflow of STAR-consensus. (*B*) Different types of mapping errors based on the read's mapping status in the individual's haploid personal genome and the reference or given consensus genome. (*C*) Overall mapping error rate for each error type for individual NA19238. Genome is shown on the *x*-axis, and the mapping error rate is shown on the *y*-axis. (*D*) Overall mapping error rate for all individuals. Individuals from the same population are grouped together by color, with each marker shape representing one individual in the population. The dashed line shows the average error rate for the population, and the solid vertical line indicates the range of the population. (*E*) Homozygous mapping error rate for each error type for individual NA19238. (*F*) Homozygous mapping error rate for all individuals. Individuals from the same population are grouped together by color, with each marker shape representing one individual in the population. The dashed line shows the average error rate for the population, and the solid vertical line indicates the range of the population. (*G*) Homozygous mapping error rates for each error type for simulated reads for individual NA12938. (*H*) Population-average homozygous mapping error rates for simulated reads for all individuals.

To simplify alignment to the consensus genome, we developed STAR-consensus, an extension to the RNA-seq aligner STAR (Fig. 2A; Dobin et al. 2013). It imports variants from a VCF file and incorporates them into the reference genome sequence, thus creating a transformed genome for mapping. Importantly, after mapping the reads to the transformed genome, STAR-consensus can reverse the alignment coordinates back to the original reference genome coordinates. This transformation is nontrivial when insertion or deletion variants are included and allows performing all downstream analyses in the reference coordinate system. Such an approach is an incremental step toward taking advantage of the consensus genome while at the same time using the conventional coordinate system.

The summary statistics for alignments to the reference and consensus genomes are presented in Supplemental Table S1. The changes in the overall mapping rates are marginal because only a small proportion of reads overlap the MARs. The effect is more pronounced for reads that overlap personal homozygous SNPs (2.2% of all reads): The unique mapping rate for such reads increases from 92.6% to 94.5%, whereas the mismatch error rate is reduced from 1.3% to 0.5%. Similar effects are observed for reads overlapping homozygous indels, which constitute only 0.15% of all reads.

To assess the error rate, we needed to compare the read mappings in the various genomes to ground truth. However, because the true mapping location of these reads is unknown, we used the personal haploid genome alignments as the ground truth. The personal haploid genomes correctly incorporate individual homozygous variants and thus can serve as a first-order approximation to the actual diploid personal genomes. Because typically both heterozygous alleles are present in RNA-seq reads, choosing one of them randomly (Supplemental Fig. S36) should be neutral for alignment accuracy on average (i.e., it should not make the alignment better or worse). Hence, the haploid genome is a good proxy for the actual personal diploid genome as it improves the alignment accuracy of homozygous variants while not affecting the heterozygous variants.

We classified mapping errors into five types based on the change of the read's alignment status in the reference/consensus genome compared with the ground truth (Fig. 2B). The different error types are as follows: reads that are mapped uniquely in the personal genome but mapped to multiple loci in the other genome (unique to multiple), reads that are mapped to multiple loci in the personal genome but mapped uniquely in the other genome (multiple to unique), reads that mapped to the personal genome but not to the other genome (mapped to unmapped), reads that did not map to the personal genome but did map to the other genome (unmapped to mapped), and reads that mapped uniquely in both genomes but to different positions (different mapping loci). The mapping error rate for an error type is defined as the number of erroneously mapped reads normalized by the total number of reads from an individual.

For each individual, we calculated the error rates for mapping to the reference and their respective consensus genomes (pan-human, superpopulation, population). Figure 2C shows the overall error rates for each error type for the individual NA19238. The most significant error comes from the reads that switch from mapping uniquely in the personal genome to mapping to multiple loci in the reference/consensus genomes, followed by reads that map to multiple loci in the personal genome but map uniquely in the reference/consensus.

We also separately plotted the error rate for reads that overlap indel variants (Supplemental Fig. S6), which are very small compared with the overall error rates in Figure 2C. These plots look similar for the other individuals (Supplemental Figs. S7–S20).

Figure 2D shows the overall mapping error rate for all eight individuals, summed over the five error types. We see a noticeable decrease in the error rate when the reference genome is replaced with the pan-human consensus. Additionally, increasing population specificity to the superpopulation or population consensus does not result in a significant further reduction of the error rate. This trend mirrors the observation about the minor alleles discussed above (Fig. 1E,F) and supports the conjecture that the majority of the mapping accuracy improvement is captured by the pan-human consensus, with little additional benefit from the superpopulation or population consensuses.

Replacement of the minor alleles in the reference with the major alleles in the consensus can only correct the mapping errors caused by the homozygous alternative alleles in an individual. Of course, the actual individual genome is diploid and contains millions of heterozygous variants (i.e., both the major and minor alleles are present), which cannot be truthfully represented in a haploid reference or consensus genome. To elucidate this issue, we defined the homozygous mapping error rate as the number of erroneously mapped reads that overlap homozygous variants normalized by the total number of reads overlapping homozygous variants for an individual. The homozygous mapping error rate shows the effect of different genomes, specifically on read alignments that can be affected by these genomes. Because the genomes used in this study are all haploid, we do not expect reads that overlap heterozygous variants to be significantly affected by the specific genome used.

We plotted the homozygous mapping error rates for the individual NA19238 (for each error type) in Figure 2E and all eight individuals (summed over all error types) in Figure 2F. Compared with Figure 2C and D, the homozygous error rates (Fig. 2E,F) show a much steeper decrease when the reference genome is replaced with the pan-human consensus. Additionally, the heterozygous error rate is higher than the homozygous error rate and stays relatively constant across all genomes (Supplemental Figs. S21–S28). This supports the notion that consensus genomes significantly improve the mapping accuracy of the reads that overlap homozygous variants; however, owing to their haploid nature, they cannot improve the alignment of the reads overlapping heterozygous loci.

We have investigated the robustness of our results with respect to the consensus allele definition (Supplemental Fig. S30). We see significant improvement in mapping accuracy even for relaxed (AF > 40%) or stringent (AF > 60%) major allele frequency thresholds, only slightly different from the standard definition (AF > 50%). These results show that the consensus genome benefits do not strongly depend on the precise definition of the consensus alleles or the databases used to calculate allele frequencies. This is not surprising because the main accuracy improvements are owing to the elimination of relatively rare minor alleles from the reference. We find the same trends for RNA-seq data for 100 European and African individuals sequenced by the Geuvadis (Lappalainen et al. 2013; Fairley et al. 2020) consortium (Supplemental Fig. S29).

In the calculations above, the error rates were defined relative to the personal genome alignments, which were considered the ground truth. To corroborate our findings, we simulated reads from the personal genomes of each individual and calculated the error rate with respect to the true read loci (Fig. 2G,H). The simulated error rates show a significant reduction when switching from the reference to the pan-human consensus, and a much smaller decrease for superpopulation and population consensuses, very similar to the results obtained for real RNA-seq data (Fig. 2E,F).

To further test the robustness and generalizability of these results, we also analyzed the mapping error rate reduction in consensus genomes for another popular RNA-seq aligner, HISAT2 (Kim et al. 2019). Supplemental Figure S31 shows that the trends for HISAT2-mapped reads are qualitatively similar to our STAR results (Fig. 2). These results show that consensus genomes will be advantageous regardless of the alignment algorithm used.

### Mapping RNA-seq reads to unrelated consensus genomes outperforms the reference

**Figure 3.**
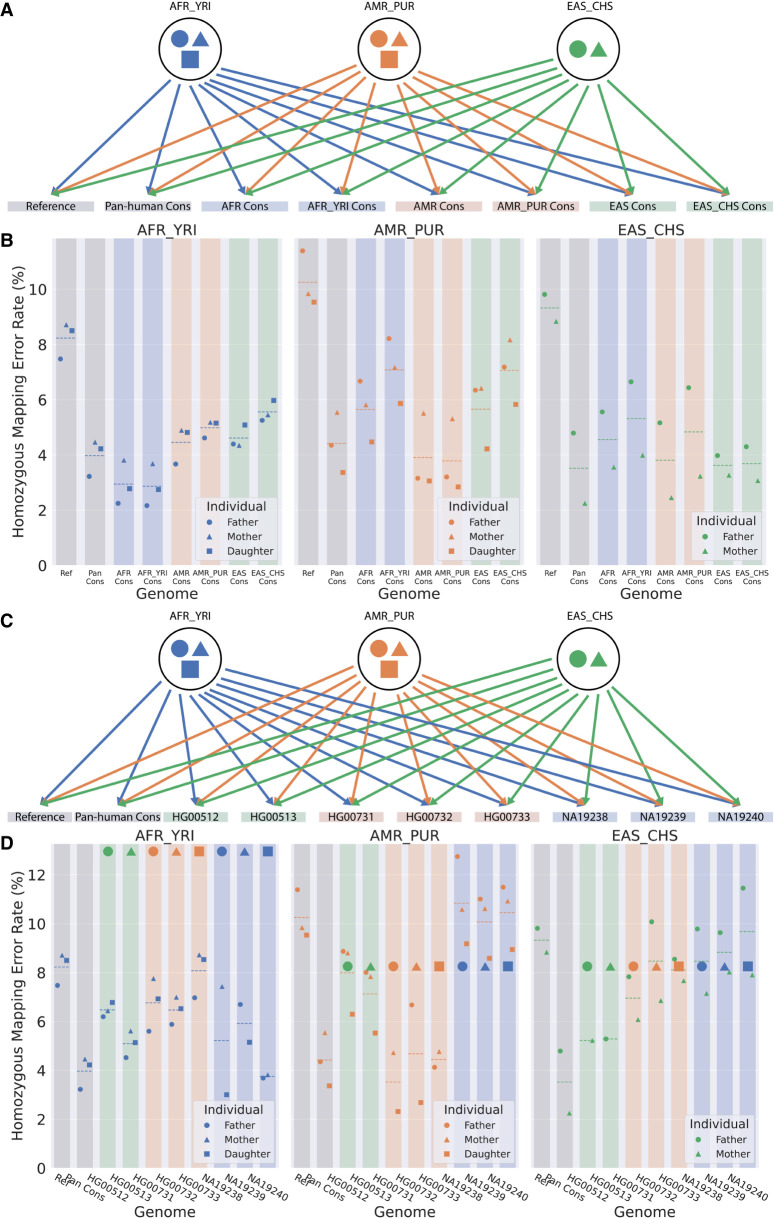
Mapping accuracy improvements owing to switching from reference to consensus genomes when mapping to alternative genomes. (*A*) Each individual from each population is independently mapped to the reference, pan-human consensus, and all population and superpopulation consensus genomes. (*B*) Homozygous mapping error rate when mapping to different consensus. The color of the marker indicates the population to which that individual belongs, whereas the shape of the marker identifies the individual within the trio. The color of the background rectangle indicates the population of the genome. The dashed line in each column represents the mean mapping error for that combination of genome and individuals. (*C*) Each individual from each population is independently mapped to the reference, pan-human consensus, and all personal haploid genomes. (*D*) Homozygous mapping error rate when mapping to different personal haploid genomes.

We investigated the effects of mapping an individual's RNA-seq reads to consensus genomes of different populations (Fig. 3A) and other personal haploid genomes (Fig. 3C). We used the same reads, individuals, and genomes as previously discussed and mapped all individuals to all genomes. The homozygous mapping error rate is calculated as before and is shown in Figure 3, B and D.

As expected, Figure 3B shows that the unrelated consensus genomes perform worse than both the related population consensus and the pan-human consensus because each population consensus contains many major alleles unique to that population. On the other hand, unrelated consensus genomes still perform better than the reference. This is explained by the fact that the reference contains a large number of minor alleles specific to the individuals who contributed to the reference assembly. Conversely, the personal genomes of unrelated individuals are unlikely to share many MARs. This is illustrated in Figure 3D: The mapping error rate to personal genomes from different populations is higher than mapping to the pan-human consensus and is comparable with mapping to the reference. Even mapping to the unrelated individual genome from the same population (mother to father and father to mother) does not improve the accuracy significantly. However, because the daughter in each trio will share many of her MARs with her parents, we see the error rates for mapping daughters’ RNA-seq reads to their parents’ genome (and vice versa) are slightly better than mapping to the pan-human consensus.

The results show that the reference genome performs worse than any consensus genome, even consensuses from a different population. The accuracy of mapping to the reference is comparable to mapping to unrelated personal genomes. On the other hand, the pan-human consensus outperforms mapping to the unrelated individual genomes of the same or different population, and its performance is comparable with mapping to the genomes of related individuals (parent to child).

### MARs affect gene sequences

**Figure 4.**
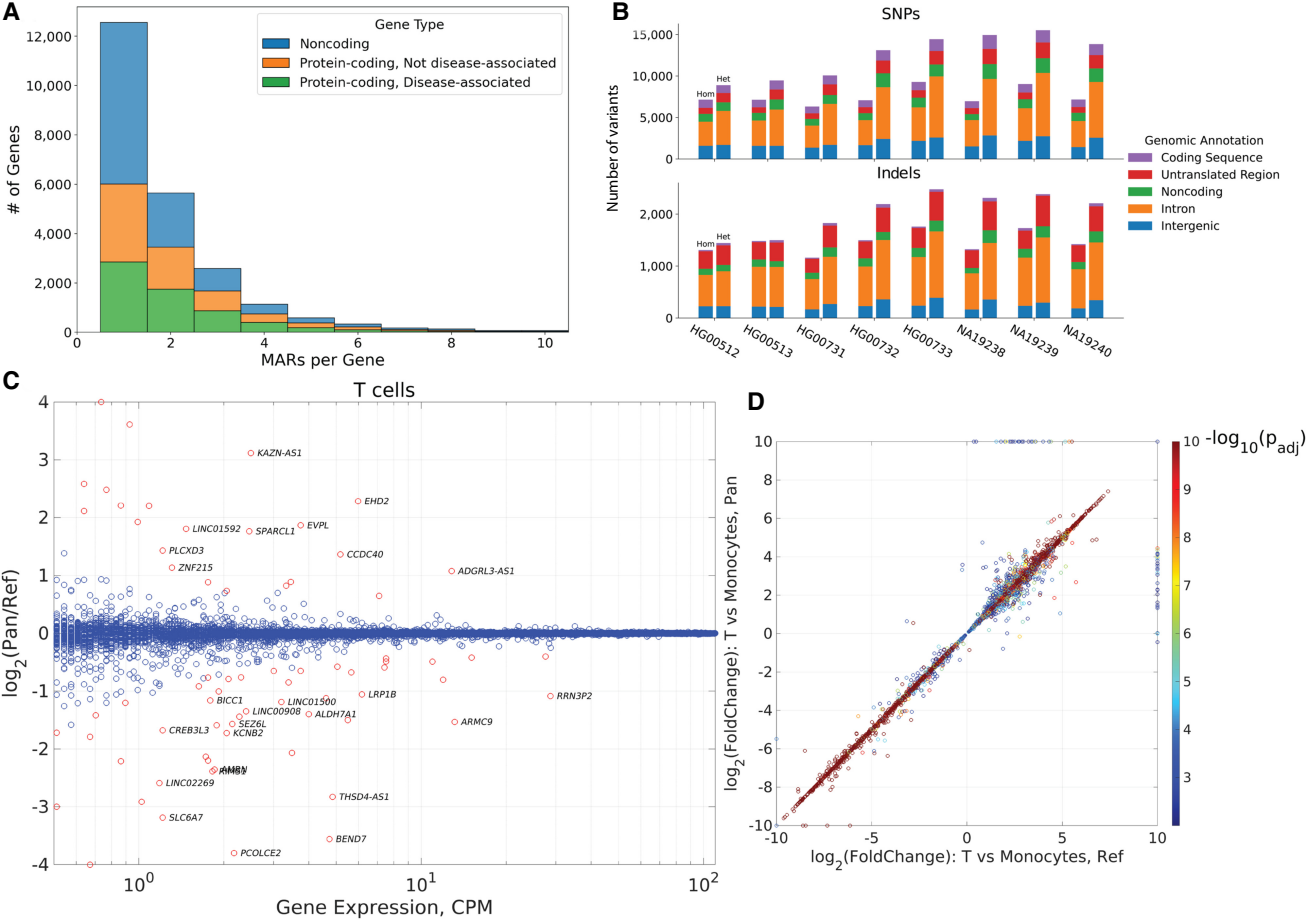
Functional effects of replacing the reference genome with a consensus. (*A*) Histogram of the number of MARs in the exons of noncoding, protein-coding, and disease-associated genes. (*B*) Counts of variants in the personal haploid genome that cause mapping errors in the reference, classified by the genomic feature in which the variant is located. For each set of bars, the *left* bar shows the number of homozygous variants, and the *right* bar indicates the number of heterozygous variants. (*C*) The gene expression log_2_ fold change between the pan-human consensus and the reference genome as a function of the maximum expression in counts per million of the T cell cluster. Red circles indicate genes with an adjusted *P*-value < 0.1. (*D*) Comparison of the gene expression log_2_ fold change between T cells and monocytes in the pan-human consensus and the reference genome. The log_2_ fold change values were capped between −10 and 10.

To investigate the genomic mechanisms underlying these mapping errors, we classified the genomic loci of the error-causing variants by overlapping error-causing reads with the GENCODE v29 annotations. Only a small proportion of the error-causing variants occur in the coding regions, whereas most are located in the intronic regions, followed by UTRs and intergenic regions (Fig. 4B). Because poly(A)^+^ RNA-seq reads should generally not map to introns, these errors are likely attributable to reads switching between being uniquely mapped and mapping to multiple locations (unique to multiple and multiple to unique error types). This corresponds with the previous observation that the largest sources of errors were the unique to multiple and multiple to unique error types.

The distribution of MARs in the exons of annotated genes is shown in Figure 4A. Overall, 12,000 protein-coding and 11,000 noncoding genes contain at least one exonic MAR. Approximately 50% of the protein-coding genes containing MARs have known disease associations (Piñero et al. 2016). Although most genes contain fewer than 10 MARs, 235 genes carry more than 10 MARs. These results show that the many transcript sequences in the current reference contain minor alleles, which are replaced with more representative major alleles in the consensus genome. Of course, even larger numbers of MARs per gene are located in the intronic regions (Supplemental Fig. S32).

### MARs affect gene expression

Here, we exemplify the effects of replacing the reference with a consensus genome on gene expression in a single-cell RNA-seq data set. The prevalent droplet-based single-cell sequencing technologies allow studying the differential transcriptomic programs between cell types. Because a large proportion of reads generated by these technologies originate from UTRs and introns, they are especially susceptible to incorrect mapping owing to minor alleles in the reference. In this example, we used the peripheral blood mononuclear cell data set generated by the 10x Chromium v3 protocol. The changes in the gene expression between the pan-human and reference genomes are shown in Figure 4C for the T cell cluster. Although the gene expression changes are small for the majority of the genes, several genes (red circles) show a significant change in expression when minor alleles in the reference are replaced with major alleles (Supplemental Table S2). The genes that show increased expression (23 genes, 12 protein-coding, seven disease-associated) in the pan-human consensus represent an improvement in sensitivity. On the other hand, the genes whose expression is higher in the reference (51 genes, 31 protein-coding, 22 disease-associated) are false positives that are eliminated in the consensus genome.

This effect can also be observed in the differential gene expression between the different clusters. Figure 4D shows how the differential gene expression between T cells and monocytes changes when the reference is replaced with the consensus genome. Although, as before, only several genes are impacted, the biological interpretation for such genes will be significantly altered by the consensus genome. Given these observations, we can conjecture that other gene expression–based analyses, such as eQTL and TWAS, can also be improved by replacing the reference genome with the pan-human consensus. Furthermore, we found that analyses that go beyond gene expression, such as alternative splicing and differential isoform expression, are also noticeably affected by the reference replacement with the consensus (Supplemental Figs. S34, S35).

## Discussion

In any data analysis, often a first central question is how much variation to include. This might be accomplished by dimension reduction, quality control, feature selection, stratification, or other techniques. The human genome is no exception, and considering how best it should be summarized remains a crucial problem, which may have a use-dependent solution: What is essential for disease variant detection may not be necessary for RNA-seq alignment, and vice versa. The current reference genome has had enormous utility, and before tearing down the infrastructure that has been built up to exploit it, it is important to consider alternatives carefully. Graph genome methods are one promising option, and they resolve the primary deficiency in the reference: effectively incorporating all variation (or aspiring to). However, this comprehensiveness comes with its own host of issues, such as the lack of a simple coordinate system, difficulties with visualization, and significantly inflated computing requirements. The wide adoption of a graph-based reference genome will likely take a long time, given the history of switching from one version of the linear reference to the next: GRCh38 was released in December 2013 (https://genome.ucsc.edu/FAQ/FAQreleases.html), and at the time of this writing, over eight years later, studies are still being published using GRCh37.

Although the full adoption of a graph genome may be several years in the future, the path there need not be a straight line. We may explore methods that partially improve on the current reference while imposing a fraction of the costs of the graph methods. By progressively assessing the role of population variation (in essence, moving from low principal components to higher ones), we can develop intermediate forms moving from the current reference to more accurate reflections of population variation, particularly ones that still opt to summarize variability to some degree. The consensus genomes have substantial utility at the pan-human level and then show a fall off past that point, suggesting that the pan-human consensus can be considered a first step in the direction of adding population variation information to the reference. Although consensus genomes are unable to represent all human genotypic variation comprehensively, they are still a desirable alternative to the reference as they eliminate the millions of spurious minor alleles present in the current reference genome while maintaining a simple linear coordinate system.

Second-order approximations to the consensus reference have also been proposed. For instance, in the MajorFlow (Chen et al. 2021) algorithm, reads are mapped to a collection of reference genomes incorporating population variation, and the reference with the best alignment for each read is selected. Applying this methodology to RNA-seq data is an exciting possibility to be explored in future work.

Consensus genomes have a straightforward representation in graph genomes: The consensus sequence is the locally most probable linear path in the variation graph genome (i.e., the path where alternative variants with the highest population frequency are selected). Thus, consensus genomes can be thought of as a first-order approximation of the full variation graph genome. Graph-based aligners, such as VG (Hickey et al. 2020), HISAT2 (Kim et al. 2019), and minigraph (Li et al. 2020), have been shown to increase the accuracy of mapping. We can envision that after computing alignments as paths through the variation graph, these aligners can project the graph alignments into the linear consensus path, hence allowing for a more straightforward output that is more compatible with the downstream processing pipelines.

This study explored the advantages and limitations of using consensus genomes for RNA-seq mapping. We used read alignments to the haploid personal genome as a proxy for the ground truth to quantify the rate of erroneous alignments to the reference genome and compared it to the three levels of consensus: pan-human, superpopulation, and population.

The overall mapping error rate caused by reference shortcomings is relatively small at only ∼0.5%–0.6% of all reads for the reference genome and further reduced to 0.3%–0.4% for the consensus genomes, leaving relatively small room for further improvements (Fig. 2D). However, for some analyses, such as allele-specific expression or de novo variant calling, the only reads of interest are those that overlap the variants. If we normalize the number of the erroneous reads by the number of reads that overlap the personal variants for each individual, we observe much higher corresponding error rates of ∼8%–10%, which decrease to ∼2%–4% when using a consensus genome.

The homozygous error rate (defined for reads that overlap only homozygous variants) is substantially decreased (by approximately two- to threefold) when the pan-human consensus replaces the reference genome. Using the superpopulation or population consensuses does not further improve the mapping accuracy, indicating that the pan-human consensus captures most population variation information that can be captured in a linear haploid genome. Using the superpopulation or population consensus genomes may not be worth the loss of generality: For instance, it will severely complicate interpopulation comparisons owing to the lack of a standard coordinate system.

These mapping results call into question the time and resources spent on constructing consensus genomes for particular populations (Cho et al. 2016; Fakhro et al. 2016; Higasa et al. 2016; Sherman et al. 2019; Takayama et al. 2021). One would expect that more specific consensus genomes would increase the mapping accuracy for the populations they represent. However, our results indicate that a universal pan-human consensus genome is sufficient to attain the best possible accuracy that can be achieved with a haploid reference, and the expensive efforts to construct more population-specific references are likely futile for improving the accuracy of RNA-seq analyses.

On the other hand, the heterozygous error rate (for reads that overlap heterozygous variants) is not significantly reduced by replacing the reference with a consensus of any population level. This is not surprising given that the haploid genome can only include one of the alleles of a heterozygous locus and hence cannot truthfully represent it. Graph genomes or other nonlinear reference representations will be required to reduce error rates for heterozygous loci.

Although there is still work to be performed on improving the reference genome, the pan-human consensus already offers noticeable improvements in downstream analyses, as indicated by the difference in splice junction expression quantification. We showed that the accuracy of the splice junction quantification is significantly improved by switching from the reference to the pan-human consensus. These improvements imply important consequences in functional analyses such as alternative splicing, transcript abundance quantification, and differential isoform usage. Splice junction differences are subtle, but the fivefold difference in the number of splice junctions with a higher quantification error in the reference than in the pan-human consensus shows that the pan-human consensus offers meaningful improvements over the reference. Results from a similar analysis of gene isoform expression (Supplemental Information) provide additional support for this claim.

At the same time, mapping to the consensus genome instead of the reference leads to marginal increases in computational time (∼2%) (Supplemental Fig. S33) and memory (∼10%, from 29 GB to 32 GB). These increases are driven mainly by the need to convert the consensus alignment coordinates to the reference coordinates, which will be eliminated if the consensus genome becomes the reference.

This study was focused on the benefits of a consensus reference for RNA-seq analyses. To illustrate that these results can be generalized for other types of functional sequencing assays, we calculated mapping error rates for the H3K4me3 histone modification ChIP-seq data set from the ENCODE consortium (Supplemental Fig. S37). Similar to the RNA-seq results, the error rate for reads overlapping homozygous variants is reduced from 6.1% to 1.9% when the reference is replaced with the pan-human consensus, whereas population-specific consensuses do not improve the accuracy significantly.

This study only considered single-nucleotide variants and small insertions/deletions. Large structural variants can add or remove large sequence fragments from the genome (Sherman et al. 2019) and thus may have an even bigger effect on mapping accuracy. The new generation of long-read technologies shows promise for the confident detection of large structural variants. However, at this time, allele frequency information is unavailable for large structural variants, and thus, they cannot be included in the consensus reference construction.

Ultimately, the best reference sequence for each individual is their own personal genome. As sequencing costs are rapidly decreasing, personal genomes are becoming more available. Nevertheless, there will be a need for a common reference capable of representing the analysis results in a universal coordinate system.

The pan-human consensus appears to be a strict improvement over the current reference with minimal costs, and thus, we propose replacing the current reference with the pan-human consensus. Besides the question of absolute utility, we also advocate using consensus genomes as a mechanism to develop practices to improve genome representation more generally. Recent years have seen genomics pipelines using the reference become entrenched, to varying degrees, by researchers unwilling to upgrade. Because the consensus genome requires minor changes in pipelines, it can be used as a straightforward, first-order approximation to assess and explore the sensitivity of specific genomic analyses to genome variation. For instance, the benefits of the consensus genome for RNA-seq mapping can be explored via the STAR-consensus pipeline, which aligns reads to the consensus genome and then transforms the coordinates to the reference genome coordinates, thus eliminating the need for changes in the downstream processing. By incorporating consensus genomes, we envision not only improvements in the absolute performance of diverse research projects but also a greater understanding of the dependencies in those methods, thus setting the stage for a more flexible and robust future for genomics.

## Methods

### Calculating consensus alleles

We calculated the consensus allele for each variant on a per-haplotype basis: The number of occurrences of each allele was counted, and the most common allele was selected. For the pan-human consensus, the alleles were counted across all individuals. For each superpopulation and population consensus, the alleles were counted across all individuals within that group. This counting was performed in Python by ConsDB by reading through each VCF file one line at a time and parsing the genotype for each individual in the group for which the consensus is being constructed.

### Genome generation and read mapping

All genome generation and read mapping were performed with STAR v2.7.7a (Dobin et al. 2013). We used GRCh38 (Schneider et al. 2017) as the reference FASTA file and GENCODE v29 (Frankish et al. 2019) as the reference GTF file. We masked the PAR regions on the Y Chromosome to avoid any sex-based differences in mapping. For the generation of consensus and personal haploid genomes, we used the ‐‐genomeTransformType Haploid option and the ‐‐genomeTransformVCF option with the appropriate VCF file. For the read mapping, we used the ‐‐genomeTransformOutput SAM SJ and the ‐‐quantMode GeneCounts TranscriptomeSAM options. We also used the ‐‐outSAMreadID Number option in order to keep track of reads in the analysis steps more easily. Other than these options, we used the default STAR parameters.

### Mapping error calculations

Before calculating the mapping error, we made several preparations. First, we used awk to construct VCF files that contained only the individual's phased genotype. Next, we used these full VCFs to partition the variants for each consensus genome for each individual into four separate VCF files: one for homozygous SNPs, one for heterozygous SNPs, one for homozygous indels, and one for heterozygous indels. These four split VCFs needed to be generated for each individual, including individuals from within the same population, because variants may be homozygous in one individual but heterozygous in a different individual.

For each individual, filtered alignments for the reference, pan-human consensus, superpopulation consensus, and population consensus were compared with the filtered alignment for their personal haploid genome using an awk script. We compared the genomes on a per-read basis, checking for differences in mapping position and number of mapped loci. To determine what types of variants each read overlapped, we overlapped the filtered BAM files with each of the four split VCF files using BEDTools, for each genome and each individual. We compared the read IDs from this overlap with the read IDs obtained from the genome mapping comparisons using grep in order to find error-causing variants.

The final steps of read counting and plotting were performed using a Python script. For each individual, we summed the read counts for each combination of error type and homozygous/heterozygous variants across all four genomes being analyzed. The two normalization constants used for these figures were the total number of mapped reads for each individual and the total number of reads that overlapped personal homozygous variants. The total mapped read numbers were extracted from the STAR Log.final.out file. The counts of reads overlapping personal homozygous variants were found by counting the number of reads present in the previously found overlap files for reads overlapping homozygous variants in the personal haploid genome.

We applied the same mapping error calculation methodology to the ENCODE H3K4me3 ChIP-seq data set for the GM12878 cell line derived from The 1000 Genomes Project Consortium individual NA12878. The FASTQ files ENCFF598WCX and ENCFF825QGB were downloaded from the ENCODE portal. STAR was run with an additional –alignIntronMax 1 option to prohibit spliced alignments.

### Special considerations for reads aligned with HISAT2

Because HISAT2 does not have the same consensus-to-reference transformation capabilities as STAR-Consensus, the mapping error calculation pipeline must be adjusted to work with HISAT2. First, each individual's personal VCF file and all consensus VCF files were collapsed to remove overlapping variants, following the same procedure that the BCFtools consensus command uses. This variant filtering was performed using a custom Python script available in the accompanying GitHub repository. Following the VCF filtering, a FASTA file was generated for each individual and each consensus using these reduced VCF files with BCFtools. At this point, the standard HISAT2 index-generation and mapping commands were used. Because the coordinate system for the HISAT2 alignments was specific for each genome used, we used levioSAM (Mun et al. 2021) in conjunction with the previously generated VCF files to transform the alignment coordinates back to the reference. Other than this liftOver step, the comparison of alignments was identical to the pipeline used with the STAR-Consensus results.

### Simulating reads with personal variants

We used the following procedure to simulate personal reads and compare their alignments to the consensus genomes with the true coordinates. First, the sequences of all annotated transcripts were extracted from the reference genome, and each base of these sequences was associated with the reference coordinate. Next, we modified the transcript sequences for each individual using their personal single-nucleotide variants and indels, both homo- and heterozygous, which resulted in two haplotypes for each transcript. We then extracted the 50-bp read sequences from both personal haplotypes, covering all transcripts uniformly. The true coordinates of these reads in the reference genome were taken from the information recorded in the first step. Sequences that appeared multiple times in the personal genome were eliminated. Finally, we aligned the reads to the consensus genomes using STAR-Consensus and transformed the alignments to the reference coordinates, allowing us to compare their mapped positions to the true simulated coordinates.

### Finding error-causing variant locations

To find the genomic annotations of error-causing variants, we first selected the error-causing variants as described above. We next used BEDTools to intersect these variants with the GENCODE v29 (Frankish et al. 2019) GTF file and find all genomic annotations that each variant overlaps. Because certain genomic annotations always fall within other genomic annotations (e.g., an exon will necessarily be located within a gene), a given variant is likely to have multiple genomic annotations that it overlaps. We used a Python script to determine the most specific genomic annotation overlapped by each variant and count the number of variants falling within each type of genomic annotation.

### Processing single-cell RNA-seq data set

We used the STARsolo gene/count matrix generated with the ‐‐soloFeatures GeneFull_ExonOverIntron option as a starting point for the SCANPY (Wolf et al. 2018) 1.6.0 pipeline. We used the Leiden clustering algorithm to identify four main clusters: T cells, B cells, natural killer (NK) cells, and monocytes. The differentially expressed genes in each cluster were evaluated using SCANPY's implementation of the *t*-test.

### Software availability

The ConsDB package is available at GitHub (https://github.com/kaminow/consdb). STAR-consensus is available at GitHub (https://github.com/alexdobin/star). Scripts to reproduce the analysis in this study, including the Supplemental Information and Supplemental Figures, are also available at GitHub (https://github.com/kaminow/ConsDB_analysis) and as Supplemental Code.

## Supplementary Material

Supplemental Material
