# Supplementary material for: Pan-human consensus genome significantly improves the accuracy of RNA-seq analyses
Source: Genome Res. 2022 Apr;32(4):738–49. doi: 10.1101/gr.275613.121 (PMC8997357; doi:10.1101/gr.275613.121)
Supplement: Supplemental Material [file supp_32_4_738__DC1.html]

Pan-human consensus genome significantly improves the accuracy of RNA-seq analyses — Supplemental Material 

# Pan-human consensus genome significantly improves the accuracy of RNA-seq analyses

## Supplemental Material

- Supplemental\_Code.zip
- Supplemental\_Information.pdf
- Supplemental\_Tables.pdf
- Supplemental\_Figures.pdf
