# Supplementary material for: Pan-human consensus genome significantly improves the accuracy of RNA-seq analyses
Source: Genome Res. 2022 Apr;32(4):738–49. doi: 10.1101/gr.275613.121 (PMC8997357; doi:10.1101/gr.275613.121)
Supplement: Supplemental Material [file supp_gr.275613.121_Supplemental_Code.zip › Supplemental_Code/ConsDB/docs/annotated.html]

ConsDB: Class List


|  |
| --- |
| ConsDB  1.0  Tool for creating consensus genomes from variant databases. |


Class List

Here are the classes, structs, unions and interfaces with brief descriptions:

[detail level 123]

|  |  |
| --- | --- |
| ▼N**RSEntry** |  |
| CRSCollection |  |
| ▼CRSEntry |  |
| CRSVar |  |
| ▼N**SlimRSCollection** |  |
| CBitRSCollection |  |


---

Generated by  

 1.8.17
