# Supplementary material for: Pan-human consensus genome significantly improves the accuracy of RNA-seq analyses
Source: Genome Res. 2022 Apr;32(4):738–49. doi: 10.1101/gr.275613.121 (PMC8997357; doi:10.1101/gr.275613.121)
Supplement: Supplemental Material [file supp_gr.275613.121_Supplemental_Code.zip › Supplemental_Code/ConsDB/docs/classes.html]

ConsDB: Class Index


|  |
| --- |
| ConsDB  1.0  Tool for creating consensus genomes from variant databases. |


Class Index

b | r

|  |  |  |  |
| --- | --- | --- | --- |
| |  | | --- | | b | | |  | | --- | | r | | RSEntry (RSEntry) |  |
|  |  | RSEntry.RSVar (RSEntry) |  |
| BitRSCollection (SlimRSCollection) | RSCollection (RSEntry) |  |  |
|  |  |  |  |

b | r


---

Generated by  

 1.8.17
