# Supplementary material for: Pan-human consensus genome significantly improves the accuracy of RNA-seq analyses
Source: Genome Res. 2022 Apr;32(4):738–49. doi: 10.1101/gr.275613.121 (PMC8997357; doi:10.1101/gr.275613.121)
Supplement: Supplemental Material [file supp_gr.275613.121_Supplemental_Code.zip › Supplemental_Code/ConsDB/docs/classRSEntry_1_1RSCollection-members.html]

ConsDB: Member List


|  |
| --- |
| ConsDB  1.0  Tool for creating consensus genomes from variant databases. |


- **RSEntry**
- RSCollection

RSEntry.RSCollection Member List

This is the complete list of members for RSEntry.RSCollection, including all inherited members.

|  |  |  |
| --- | --- | --- |
| \_\_add\_\_(self, rsc) | RSEntry.RSCollection |  |
| \_\_eq\_\_(self, rsc) | RSEntry.RSCollection |  |
| \_\_getitem\_\_(self, key) | RSEntry.RSCollection |  |
| \_\_iadd\_\_(self, rsc) | RSEntry.RSCollection |  |
| **\_\_init\_\_**(self) (defined in RSEntry.RSCollection) | RSEntry.RSCollection |  |
| \_\_len\_\_(self) | RSEntry.RSCollection |  |
| \_\_repr\_\_(self) | RSEntry.RSCollection |  |
| \_\_str\_\_(self) | RSEntry.RSCollection |  |
| add\_entry(self, e) | RSEntry.RSCollection |  |
| add\_entry\_from\_args(self, chrom, rsid, pos, quiet=False) | RSEntry.RSCollection |  |
| **chr\_pos\_table** (defined in RSEntry.RSCollection) | RSEntry.RSCollection |  |
| chrom\_to\_int(c) | RSEntry.RSCollection | static |
| dump(self, fn, idx\_file, c, rsids=['all'], old\_size=0, append=False, chunksize=10000) | RSEntry.RSCollection |  |
| dump\_chrs(self, chrs, fp\_out, store\_all=False, store\_maj=False) | RSEntry.RSCollection |  |
| dump\_full(self, fn) | RSEntry.RSCollection |  |
| dump\_vcf(self, fn, pop=None, cons=False, is\_maj=False) | RSEntry.RSCollection |  |
| **entries** (defined in RSEntry.RSCollection) | RSEntry.RSCollection |  |
| from\_1000gp(fn, index\_fn, superpop\_fn, quiet=False) | RSEntry.RSCollection | static |
| from\_dbsnp(fn, quiet=False) | RSEntry.RSCollection | static |
| from\_gnomad(fn, quiet=False) | RSEntry.RSCollection | static |
| get\_by\_chr(self, chrom) | RSEntry.RSCollection |  |
| get\_by\_chr\_pos(self, chrom, pos) | RSEntry.RSCollection |  |
| get\_by\_rsid(self, rsid) | RSEntry.RSCollection |  |
| get\_chrom\_from\_filename(fn) | RSEntry.RSCollection | static |
| get\_major(self, mut=True) | RSEntry.RSCollection |  |
| load\_from\_file\_by\_chr\_pos(fn, idx\_file, c, pos, chunk\_idx\_dict=None, ret\_chunk=False) | RSEntry.RSCollection | static |
| load\_from\_file\_by\_rsid(fn, idx\_file, rsid, chunk\_idx\_dict=None, ret\_chunk=False) | RSEntry.RSCollection | static |
| load\_from\_file\_full(fn) | RSEntry.RSCollection | static |
| load\_from\_file\_pops(fn, pops, cons=False) | RSEntry.RSCollection | static |
| make\_chunk\_idx\_dict(idx\_fn, key\_fields=[1, 2]) | RSEntry.RSCollection | static |
| merge\_files(fn\_list, out\_fn, c=None) | RSEntry.RSCollection | static |
| open(fn) | RSEntry.RSCollection | static |
| parse\_file\_line(d) | RSEntry.RSCollection | static |
| **rsid\_table** (defined in RSEntry.RSCollection) | RSEntry.RSCollection |  |
| sort\_rsidx(rsidx) | RSEntry.RSCollection | static |
| sort\_rsidx\_line(line\_split) | RSEntry.RSCollection | static |


---

Generated by  

 1.8.17
