# Supplementary material for: Pan-human consensus genome significantly improves the accuracy of RNA-seq analyses
Source: Genome Res. 2022 Apr;32(4):738–49. doi: 10.1101/gr.275613.121 (PMC8997357; doi:10.1101/gr.275613.121)
Supplement: Supplemental Material [file supp_gr.275613.121_Supplemental_Code.zip › Supplemental_Code/ConsDB/docs/classRSEntry_1_1RSCollection.html]

ConsDB: RSEntry.RSCollection Class Reference


|  |
| --- |
| ConsDB  1.0  Tool for creating consensus genomes from variant databases. |


- **RSEntry**
- RSCollection

Public Member Functions |
Static Public Member Functions |
Public Attributes |
List of all members

RSEntry.RSCollection Class Reference

|  |  |
| --- | --- |
| Public Member Functions | |
| def | **\_\_init\_\_** (self) |
|  | |
| def | \_\_add\_\_ (self, rsc) |
|  | |
| def | \_\_eq\_\_ (self, rsc) |
|  | |
| def | \_\_getitem\_\_ (self, key) |
|  | |
| def | \_\_iadd\_\_ (self, rsc) |
|  | |
| def | \_\_len\_\_ (self) |
|  | |
| def | \_\_repr\_\_ (self) |
|  | |
| def | \_\_str\_\_ (self) |
|  | |
| def | add\_entry (self, e) |
|  | |
| def | add\_entry\_from\_args (self, chrom, rsid, pos, quiet=False) |
|  | |
| def | dump (self, fn, idx\_file, c, rsids=['all'], old\_size=0, append=False, chunksize=10000) |
|  | |
| def | dump\_full (self, fn) |
|  | |
| def | dump\_chrs (self, chrs, fp\_out, store\_all=False, store\_maj=False) |
|  | |
| def | dump\_vcf (self, fn, pop=None, cons=False, is\_maj=False) |
|  | |
| def | get\_by\_chr (self, chrom) |
|  | |
| def | get\_by\_chr\_pos (self, chrom, pos) |
|  | |
| def | get\_by\_rsid (self, rsid) |
|  | |
| def | get\_major (self, mut=True) |
|  | |

|  |  |
| --- | --- |
| Static Public Member Functions | |
| def | chrom\_to\_int (c) |
|  | |
| def | from\_1000gp (fn, index\_fn, superpop\_fn, quiet=False) |
|  | |
| def | from\_gnomad (fn, quiet=False) |
|  | |
| def | from\_dbsnp (fn, quiet=False) |
|  | |
| def | get\_chrom\_from\_filename (fn) |
|  | |
| def | load\_from\_file\_by\_chr\_pos (fn, idx\_file, c, pos, chunk\_idx\_dict=None, ret\_chunk=False) |
|  | |
| def | load\_from\_file\_by\_rsid (fn, idx\_file, rsid, chunk\_idx\_dict=None, ret\_chunk=False) |
|  | |
| def | load\_from\_file\_full (fn) |
|  | |
| def | load\_from\_file\_pops (fn, pops, cons=False) |
|  | |
| def | make\_chunk\_idx\_dict (idx\_fn, key\_fields=[1, 2]) |
|  | |
| def | merge\_files (fn\_list, out\_fn, c=None) |
|  | |
| def | open (fn) |
|  | |
| def | parse\_file\_line (d) |
|  | |
| def | sort\_rsidx (rsidx) |
|  | |
| def | sort\_rsidx\_line (line\_split) |
|  | |

|  |  |
| --- | --- |
| Public Attributes | |
|  | **entries** |
|  | |
|  | **chr\_pos\_table** |
|  | |
|  | **rsid\_table** |
|  | |

## Detailed Description

```
    A class to represent a collection of entries.

    Attributes
    --------------------
    entries : dict
        Dictionary that maps from (rsid, chrom, pos) to RSEntry object
    chr_pos_table : dict
        Dictionary that maps from (chrom, pos) to (rsid, chrom, pos)
    rsid_table : dict
        Dictionary that maps from rsid to (rsid, chrom, pos)

    Methods
    --------------------
    add_entry(e)
        Add entry from an RSEntry object
    add_entry_from_args(chrom, rsid, pos, quiet=False)
        Add an entry based on the given arguments
    dump(fn, idx_file, c, rsids = ['all'], old_size=0, append=False,
        chunksize=10000)
        Save entries for the given rsids to the given file
    dump_full(fn)
        Quickly save the whole RSCollection object to the given file
    dump_chrs(chrs, fp_out, store_all=False, store_maj=False)
        Save entries for the given chromosomes
    dump_vcf(fn, pop=None, cons=False, is_maj=False)
        Save entries as a VCF file
    get_by_chr(chrom)
        Return a RSCollection object containing all entries from given chrom
    get_by_chr_pos(chrom, pos)
        Return a RSCollection object containing all entries at given chrom
        and given position
    get_by_rsid(rsid)
        Return a list of RSEntry objects that match the given rsid
    get_major(mut=True)
        Return a RSCollection object containing all major alleles

    Static Methods
    --------------------
    chrom_to_int(c)
        Function to use to sort chromosomes (numeric < X < Y < M)
    from_1000gp(fn, index_fn, superpop_fn, quiet=False)
        Create a new RSCollection object from a 1000 Genomes Project VCF file
    from_gnomad(fn, quiet=False)
        Create a new RSCollection object from a gnomAD VCF file
    from_dbsnp(fn, quiet=False)
        Create a new RSCollection object from a dbSNP JSON file
    get_chrom_from_filename(fn)
        Helper method to parse a filename and find which chromosome it is
    load_from_file_by_chr_pos(fn, idx_file, c, pos, chunk_idx_dict=None,
        ret_chunk=False)
        Find an entry in the given file using given chromosome and position
    load_from_file_by_rsid(fn, idx_file, rsid, chunk_idx_dict=None,
        ret_chunk=False)
        Find an entry in the given file using given rsid
    load_from_file_full(fn)
        Load an entire ConsDB file
    load_from_file_pops(fn, pops, cons=False)
        Only load variants that are major variants for pop
    make_chunk_idx_dict(idx_fn, key_fields=[1,2])
        Make a dict representation of the idx file
    merge_files(fn_list, out_fn, c=None)
        Merge multiple ConsDB files into one
    open(fn)
        Helper method to appropriately open the given file
    parse_file_line(d)
        Create a RSEntry object from given line from ConsDB file
    sort_rsidx(rsidx)
        Helper method to sort a list of rsidx
    sort_rsidx_line(line_split)
        Helper method to sort lines from a ConsDB file
```

## Member Function Documentation

## ◆ \_\_add\_\_()

|  |  |  |  |
| --- | --- | --- | --- |
| def RSEntry.RSCollection.\_\_add\_\_ | ( |  | *self*, |
|  |  |  | *rsc* |
|  | ) |  |  |

```
Implement addition for two RSCollection objects.
```

## ◆ \_\_eq\_\_()

|  |  |  |  |
| --- | --- | --- | --- |
| def RSEntry.RSCollection.\_\_eq\_\_ | ( |  | *self*, |
|  |  |  | *rsc* |
|  | ) |  |  |

```
Implement equality checking for two RSCollection objects.
```

## ◆ \_\_getitem\_\_()

|  |  |  |  |
| --- | --- | --- | --- |
| def RSEntry.RSCollection.\_\_getitem\_\_ | ( |  | *self*, |
|  |  |  | *key* |
|  | ) |  |  |

```
Allow access to entries via the [] operator.
```

## ◆ \_\_iadd\_\_()

|  |  |  |  |
| --- | --- | --- | --- |
| def RSEntry.RSCollection.\_\_iadd\_\_ | ( |  | *self*, |
|  |  |  | *rsc* |
|  | ) |  |  |

```
Implement incremental addition.
```

## ◆ \_\_len\_\_()

|  |  |  |  |  |  |
| --- | --- | --- | --- | --- | --- |
| def RSEntry.RSCollection.\_\_len\_\_ | ( |  | *self* | ) |  |

```
Implement len operator.
```

## ◆ \_\_repr\_\_()

|  |  |  |  |  |  |
| --- | --- | --- | --- | --- | --- |
| def RSEntry.RSCollection.\_\_repr\_\_ | ( |  | *self* | ) |  |

```
Implement repr operator.
```

## ◆ \_\_str\_\_()

|  |  |  |  |  |  |
| --- | --- | --- | --- | --- | --- |
| def RSEntry.RSCollection.\_\_str\_\_ | ( |  | *self* | ) |  |

```
Implement str operator.
```

## ◆ add\_entry()

|  |  |  |  |
| --- | --- | --- | --- |
| def RSEntry.RSCollection.add\_entry | ( |  | *self*, |
|  |  |  | *e* |
|  | ) |  |  |

```
Add entry from an RSEntry object. Note that this does not create a new
RSEntry object.

Parameters:
e: RSEntry object
```

## ◆ add\_entry\_from\_args()

|  |  |  |  |
| --- | --- | --- | --- |
| def RSEntry.RSCollection.add\_entry\_from\_args | ( |  | *self*, |
|  |  |  | *chrom*, |
|  |  |  | *rsid*, |
|  |  |  | *pos*, |
|  |  |  | *quiet* = `False` |
|  | ) |  |  |

```
Add an entry based on the given arguments.

Parameters:
chrom: Chromosome of the entry being added
rsid: RefSNP ID of the entry being added
pos: Position of the entry being added
quiet: Suppress progress information being printed
```

## ◆ chrom\_to\_int()

|  |  |  |  |  |  |  |  |
| --- | --- | --- | --- | --- | --- | --- | --- |
| |  |  |  |  |  |  | | --- | --- | --- | --- | --- | --- | | def RSEntry.RSCollection.chrom\_to\_int | ( |  | *c* | ) |  | | static |

```
Function to use to sort chromosomes (numeric < X < Y < M)

Parameters:
c: Chromosome to convert
```

## ◆ dump()

|  |  |  |  |
| --- | --- | --- | --- |
| def RSEntry.RSCollection.dump | ( |  | *self*, |
|  |  |  | *fn*, |
|  |  |  | *idx\_file*, |
|  |  |  | *c*, |
|  |  |  | *rsids* = `['all']`, |
|  |  |  | *old\_size* = `0`, |
|  |  |  | *append* = `False`, |
|  |  |  | *chunksize* = `10000` |
|  | ) |  |  |

```
Save entries for the given rsids to the given file.

Split the entries to write into multiple chunks, then write each chunk
separately to the gzip file. This allows for random access when loading.

Parameters:
fn: Filename to save to
idx_file: Filename to save index information to
c: Which chromosome is being saved
rsids: Which RefSNP IDs/(chr, pos) to save
old_size: Prior size of the output file (used for random access)
append: Whether to append to the given output file
chunksize: Number of entries to write at a time
```

## ◆ dump\_chrs()

|  |  |  |  |
| --- | --- | --- | --- |
| def RSEntry.RSCollection.dump\_chrs | ( |  | *self*, |
|  |  |  | *chrs*, |
|  |  |  | *fp\_out*, |
|  |  |  | *store\_all* = `False`, |
|  |  |  | *store\_maj* = `False` |
|  | ) |  |  |

```
Save entries for the given chromosomes.

Parameters:
chrs: Chromosomes to save
fp_out: Directory to store the files
store_all: Save file with all entries
store_maj: Save file with only major alleles
```

## ◆ dump\_full()

|  |  |  |  |
| --- | --- | --- | --- |
| def RSEntry.RSCollection.dump\_full | ( |  | *self*, |
|  |  |  | *fn* |
|  | ) |  |  |

```
Quickly save the whole RSCollection object to the given file.

Parameters:
fn: Filename to save to
```

## ◆ dump\_vcf()

|  |  |  |  |
| --- | --- | --- | --- |
| def RSEntry.RSCollection.dump\_vcf | ( |  | *self*, |
|  |  |  | *fn*, |
|  |  |  | *pop* = `None`, |
|  |  |  | *cons* = `False`, |
|  |  |  | *is\_maj* = `False` |
|  | ) |  |  |

```
Save entries as a VCF file.

Parameters:
fn: Filename to save to
pop: Population to use
con: Storing a consensus VCF
is_maj: RSCollection already contains only major alleles
```

## ◆ from\_1000gp()

|  |  |  |  |  |  |  |  |  |  |  |  |  |  |  |  |  |  |  |  |  |  |
| --- | --- | --- | --- | --- | --- | --- | --- | --- | --- | --- | --- | --- | --- | --- | --- | --- | --- | --- | --- | --- | --- |
| |  |  |  |  | | --- | --- | --- | --- | | def RSEntry.RSCollection.from\_1000gp | ( |  | *fn*, | |  |  |  | *index\_fn*, | |  |  |  | *superpop\_fn*, | |  |  |  | *quiet* = `False` | |  | ) |  |  | | static |

```
Create a new RSCollection object from a 1000 Genomes Project VCF file.

Parameters:
fn: Filename to load from
index_fn: Filename of the 1000 Genomes .index file to use
superpop_fn: Filename containing superpopulation information
quiet: Disable log/progress messages
```

## ◆ from\_dbsnp()

|  |  |  |  |  |  |  |  |  |  |  |  |  |  |
| --- | --- | --- | --- | --- | --- | --- | --- | --- | --- | --- | --- | --- | --- |
| |  |  |  |  | | --- | --- | --- | --- | | def RSEntry.RSCollection.from\_dbsnp | ( |  | *fn*, | |  |  |  | *quiet* = `False` | |  | ) |  |  | | static |

```
Create a new RSCollection object from a dbSNP JSON file.

Convert each line in the file into a JSON object and perform various
checks/calculations. Each line becomes one RSEntry object.

Parameters:
fn: Filename to load
quiet: Whether to disable log messages
```

## ◆ from\_gnomad()

|  |  |  |  |  |  |  |  |  |  |  |  |  |  |
| --- | --- | --- | --- | --- | --- | --- | --- | --- | --- | --- | --- | --- | --- |
| |  |  |  |  | | --- | --- | --- | --- | | def RSEntry.RSCollection.from\_gnomad | ( |  | *fn*, | |  |  |  | *quiet* = `False` | |  | ) |  |  | | static |

```
Create a new RSCollection object from a gnomAD VCF file.

Parameters:
fn: Filename to load
quiet: Whether to disable log messages
```

## ◆ get\_by\_chr()

|  |  |  |  |
| --- | --- | --- | --- |
| def RSEntry.RSCollection.get\_by\_chr | ( |  | *self*, |
|  |  |  | *chrom* |
|  | ) |  |  |

```
Return a RSCollection object containing all entries from given chrom.

Parameters:
chrom: Chromosome to get
```

## ◆ get\_by\_chr\_pos()

|  |  |  |  |
| --- | --- | --- | --- |
| def RSEntry.RSCollection.get\_by\_chr\_pos | ( |  | *self*, |
|  |  |  | *chrom*, |
|  |  |  | *pos* |
|  | ) |  |  |

```
Return a list of all entries with given chromosome and position.

Parameters:
chrom: Chromosome to get
pos: Position to get
```

## ◆ get\_by\_rsid()

|  |  |  |  |
| --- | --- | --- | --- |
| def RSEntry.RSCollection.get\_by\_rsid | ( |  | *self*, |
|  |  |  | *rsid* |
|  | ) |  |  |

```
Return a list of all entries with given RefSNP ID number.

Parameters:
rsid: RefSNP ID number to get
```

## ◆ get\_chrom\_from\_filename()

|  |  |  |  |  |  |  |  |
| --- | --- | --- | --- | --- | --- | --- | --- |
| |  |  |  |  |  |  | | --- | --- | --- | --- | --- | --- | | def RSEntry.RSCollection.get\_chrom\_from\_filename | ( |  | *fn* | ) |  | | static |

```
Helper method to parse a filename and find which chromosome it is.

Parameters:
fn: Filename to use
```

## ◆ get\_major()

|  |  |  |  |
| --- | --- | --- | --- |
| def RSEntry.RSCollection.get\_major | ( |  | *self*, |
|  |  |  | *mut* = `True` |
|  | ) |  |  |

```
Return a RSCollection object containing all major alleles.

Parameters:
mut: Only include variants that are different between reference and
    alternate allele
```

## ◆ load\_from\_file\_by\_chr\_pos()

|  |  |  |  |  |  |  |  |  |  |  |  |  |  |  |  |  |  |  |  |  |  |  |  |  |  |  |  |  |  |
| --- | --- | --- | --- | --- | --- | --- | --- | --- | --- | --- | --- | --- | --- | --- | --- | --- | --- | --- | --- | --- | --- | --- | --- | --- | --- | --- | --- | --- | --- |
| |  |  |  |  | | --- | --- | --- | --- | | def RSEntry.RSCollection.load\_from\_file\_by\_chr\_pos | ( |  | *fn*, | |  |  |  | *idx\_file*, | |  |  |  | *c*, | |  |  |  | *pos*, | |  |  |  | *chunk\_idx\_dict* = `None`, | |  |  |  | *ret\_chunk* = `False` | |  | ) |  |  | | static |

```
Find an entry in the given file using given chromosome and position.

If ret_chunk is True, return both the (rsid, chrom, pos) of the matching
entry and the RSCollection chunk containing the entry. Otherwise, return
just the matching entry.

Parameters:
fn: Filename to use
idx_file: Filename of the index file (will default to {fn}.idx)
c: Chromosome to load
pos: Position to load
chunk_idx_dict: Dictionary mapping (rsid,chrom,pos) combinations to the
    chunk in the ConsDB file containing that entry
ret_chunk: Whether to return the entire chunk loaded (as opposed to only
    returning the RSEntry object)
```

## ◆ load\_from\_file\_by\_rsid()

|  |  |  |  |  |  |  |  |  |  |  |  |  |  |  |  |  |  |  |  |  |  |  |  |  |  |
| --- | --- | --- | --- | --- | --- | --- | --- | --- | --- | --- | --- | --- | --- | --- | --- | --- | --- | --- | --- | --- | --- | --- | --- | --- | --- |
| |  |  |  |  | | --- | --- | --- | --- | | def RSEntry.RSCollection.load\_from\_file\_by\_rsid | ( |  | *fn*, | |  |  |  | *idx\_file*, | |  |  |  | *rsid*, | |  |  |  | *chunk\_idx\_dict* = `None`, | |  |  |  | *ret\_chunk* = `False` | |  | ) |  |  | | static |

```
Find an entry in the given file using given rsid.

If ret_chunk is True, return both the (rsid, chrom, pos) of the matching
entry and the RSCollection chunk containing the entry. Otherwise, return
just the matching entry.

Use temporary files for compatibility with existing functions that take
filename arguments.

Parameters:
fn: Filename to load
idx_file: Filename of the index file (will default to {fn}.idx)
rsid: RefSNP ID to load
chunk_idx_dict: Dictionary mapping (rsid,chrom,pos) combinations to the
    chunk in the ConsDB file containing that entry
ret_chunk: Whether to return the entire chunk loaded (as opposed to only
    returning the RSEntry object)
```

## ◆ load\_from\_file\_full()

|  |  |  |  |  |  |  |  |
| --- | --- | --- | --- | --- | --- | --- | --- |
| |  |  |  |  |  |  | | --- | --- | --- | --- | --- | --- | | def RSEntry.RSCollection.load\_from\_file\_full | ( |  | *fn* | ) |  | | static |

```
Load an entire ConsDB file.

Parameters:
fn: Filename to load
```

## ◆ load\_from\_file\_pops()

|  |  |  |  |  |  |  |  |  |  |  |  |  |  |  |  |  |  |
| --- | --- | --- | --- | --- | --- | --- | --- | --- | --- | --- | --- | --- | --- | --- | --- | --- | --- |
| |  |  |  |  | | --- | --- | --- | --- | | def RSEntry.RSCollection.load\_from\_file\_pops | ( |  | *fn*, | |  |  |  | *pops*, | |  |  |  | *cons* = `False` | |  | ) |  |  | | static |

```
Only load variants that are major variants for pop.

Parameters:
fn: Filename to load
pops: Populations to load
cons: RSCollection object is being used for a consensus
    (will only load one allele for each position in this case)
```

## ◆ make\_chunk\_idx\_dict()

|  |  |  |  |  |  |  |  |  |  |  |  |  |  |
| --- | --- | --- | --- | --- | --- | --- | --- | --- | --- | --- | --- | --- | --- |
| |  |  |  |  | | --- | --- | --- | --- | | def RSEntry.RSCollection.make\_chunk\_idx\_dict | ( |  | *idx\_fn*, | |  |  |  | *key\_fields* = `[1,2]` | |  | ) |  |  | | static |

```
Make a dict representation of the idx file.
Useful when indexing into a file multiple times in order to avoid
reconstructing this same dict every time.

Use fields [1,2] if loading by chrom, pos. Use field 0 if loading by
RSID.

Parameters:
idx_fn: Filename of the ConsDB index file
key_fields: Which fields in the (rsid,chrom,pos) combination are to be
    used as the keys in the dict
```

## ◆ merge\_files()

|  |  |  |  |  |  |  |  |  |  |  |  |  |  |  |  |  |  |
| --- | --- | --- | --- | --- | --- | --- | --- | --- | --- | --- | --- | --- | --- | --- | --- | --- | --- |
| |  |  |  |  | | --- | --- | --- | --- | | def RSEntry.RSCollection.merge\_files | ( |  | *fn\_list*, | |  |  |  | *out\_fn*, | |  |  |  | *c* = `None` | |  | ) |  |  | | static |

```
Merge multiple ConsDB files into one.

Parameters:
fn_list: List of filenames to merge
out_fn: Filename to use for the resulting merged file
c: Chromosome of the input files
```

## ◆ open()

|  |  |  |  |  |  |  |  |
| --- | --- | --- | --- | --- | --- | --- | --- |
| |  |  |  |  |  |  | | --- | --- | --- | --- | --- | --- | | def RSEntry.RSCollection.open | ( |  | *fn* | ) |  | | static |

```
Helper method to appropriately open the given file.

Parameters:
fn: Filename to open
```

## ◆ parse\_file\_line()

|  |  |  |  |  |  |  |  |
| --- | --- | --- | --- | --- | --- | --- | --- |
| |  |  |  |  |  |  | | --- | --- | --- | --- | --- | --- | | def RSEntry.RSCollection.parse\_file\_line | ( |  | *d* | ) |  | | static |

```
Create a RSEntry object from given line from rsc file.

Parameters:
d: List containing the (rsid,chrom,pos) combination as the first entry
    and the rest of the entry string as the second entry (can be created
    by splitting the RSEntry string by ':')
```

## ◆ sort\_rsidx()

|  |  |  |  |  |  |  |  |
| --- | --- | --- | --- | --- | --- | --- | --- |
| |  |  |  |  |  |  | | --- | --- | --- | --- | --- | --- | | def RSEntry.RSCollection.sort\_rsidx | ( |  | *rsidx* | ) |  | | static |

```
Helper method to sort a list of rsidx. Meant to be used as a
key for sorting. Returns the chromosome and position of the rsidx, both
in int form.

Parameters:
rsidx: List/tuple of (rsid, chrom, pos)
```

## ◆ sort\_rsidx\_line()

|  |  |  |  |  |  |  |  |
| --- | --- | --- | --- | --- | --- | --- | --- |
| |  |  |  |  |  |  | | --- | --- | --- | --- | --- | --- | | def RSEntry.RSCollection.sort\_rsidx\_line | ( |  | *line\_split* | ) |  | | static |

```
Helper method to sort lines from a ConsDB file. Meant to be used as a
key for sorting. Returns the chromosome and position of the line, both
in int form.

Parameters:
line_split: List consisting of ['rsid,chrom,pos', 'rest of line']
```

---

The documentation for this class was generated from the following file:

- RSEntry.py


---

Generated by  

 1.8.17
