# Supplementary material for: Pan-human consensus genome significantly improves the accuracy of RNA-seq analyses
Source: Genome Res. 2022 Apr;32(4):738–49. doi: 10.1101/gr.275613.121 (PMC8997357; doi:10.1101/gr.275613.121)
Supplement: Supplemental Material [file supp_gr.275613.121_Supplemental_Code.zip › Supplemental_Code/ConsDB/docs/classRSEntry_1_1RSEntry-members.html]

ConsDB: Member List


|  |
| --- |
| ConsDB  1.0  Tool for creating consensus genomes from variant databases. |


- **RSEntry**
- RSEntry

RSEntry.RSEntry Member List

This is the complete list of members for RSEntry.RSEntry, including all inherited members.

|  |  |  |
| --- | --- | --- |
| \_\_add\_\_(self, rse) | RSEntry.RSEntry |  |
| \_\_eq\_\_(self, rse) | RSEntry.RSEntry |  |
| \_\_getitem\_\_(self, key) | RSEntry.RSEntry |  |
| \_\_iadd\_\_(self, rse) | RSEntry.RSEntry |  |
| \_\_init\_\_(self, chrom, rsid, pos=None) | RSEntry.RSEntry |  |
| \_\_len\_\_(self) | RSEntry.RSEntry |  |
| \_\_repr\_\_(self) | RSEntry.RSEntry |  |
| \_\_str\_\_(self) | RSEntry.RSEntry |  |
| add\_var(self, var) | RSEntry.RSEntry |  |
| add\_var\_from\_args(self, pos, ref, var, major=0, minor=0, clin=[], afs=[], var\_type='', pop\_afs={}) | RSEntry.RSEntry |  |
| all\_vars\_empty(self) | RSEntry.RSEntry |  |
| **chrom** (defined in RSEntry.RSEntry) | RSEntry.RSEntry |  |
| get\_major\_alleles(self) | RSEntry.RSEntry |  |
| get\_major\_alleles\_pop(self, pop) | RSEntry.RSEntry |  |
| pick\_major\_allele(self, var\_list) | RSEntry.RSEntry |  |
| **pos** (defined in RSEntry.RSEntry) | RSEntry.RSEntry |  |
| **rsid** (defined in RSEntry.RSEntry) | RSEntry.RSEntry |  |
| to\_vcf(self, pop=None, cons=False, is\_maj=False) | RSEntry.RSEntry |  |
| **vars** (defined in RSEntry.RSEntry) | RSEntry.RSEntry |  |


---

Generated by  

 1.8.17
