# Supplementary material for: Pan-human consensus genome significantly improves the accuracy of RNA-seq analyses
Source: Genome Res. 2022 Apr;32(4):738–49. doi: 10.1101/gr.275613.121 (PMC8997357; doi:10.1101/gr.275613.121)
Supplement: Supplemental Material [file supp_gr.275613.121_Supplemental_Code.zip › Supplemental_Code/ConsDB/docs/classRSEntry_1_1RSEntry.html]

ConsDB: RSEntry.RSEntry Class Reference


|  |
| --- |
| ConsDB  1.0  Tool for creating consensus genomes from variant databases. |


- **RSEntry**
- RSEntry

Classes |
Public Member Functions |
Public Attributes |
List of all members

RSEntry.RSEntry Class Reference

|  |  |
| --- | --- |
| Classes | |
| class | RSVar |
|  | |

|  |  |
| --- | --- |
| Public Member Functions | |
| def | \_\_init\_\_ (self, chrom, rsid, pos=None) |
|  | |
| def | \_\_add\_\_ (self, rse) |
|  | |
| def | \_\_eq\_\_ (self, rse) |
|  | |
| def | \_\_getitem\_\_ (self, key) |
|  | |
| def | \_\_iadd\_\_ (self, rse) |
|  | |
| def | \_\_len\_\_ (self) |
|  | |
| def | \_\_repr\_\_ (self) |
|  | |
| def | \_\_str\_\_ (self) |
|  | |
| def | add\_var (self, var) |
|  | |
| def | add\_var\_from\_args (self, pos, ref, var, major=0, minor=0, clin=[], afs=[], var\_type='', pop\_afs={}) |
|  | |
| def | all\_vars\_empty (self) |
|  | |
| def | get\_major\_alleles (self) |
|  | |
| def | get\_major\_alleles\_pop (self, pop) |
|  | |
| def | pick\_major\_allele (self, var\_list) |
|  | |
| def | to\_vcf (self, pop=None, cons=False, is\_maj=False) |
|  | |

|  |  |
| --- | --- |
| Public Attributes | |
|  | **chrom** |
|  | |
|  | **rsid** |
|  | |
|  | **pos** |
|  | |
|  | **vars** |
|  | |

## Detailed Description

```
A class to hold all variants at a given position.

Attributes
--------------------
chrom : str
    Which chromosome the entry is on (1-22, X, Y)
rsid : int
    The RefSNP ID number for the entry
pos : int
    Base position of the entry (1-indexed)
vars : dict
    Dictionary that maps variant code strings to RSVariant objects

Methods
--------------------
add_var(var)
    Add a RSVariant object to the RSEntry
add_var_from_args(pos, ref, var, major=0, minor=0, clin=[], afs=[],
    var_type='', pop_afs={})
    Add a variant to the RSEntry based on the given arguments
all_vars_empty()
    Check if all variants in the RSEntry are empty
get_major_alleles()
    Return a list of major RSVariants
get_major_alleles_pop(pop)
    Return a list of major RSVariants for a given population
pick_major_allele(var_list)
    Reproducibly choose a representative variant
to_vcf(pop=None, cons=False, is_maj=False)
    Create a VCF record from variants
```

## Constructor & Destructor Documentation

## ◆ \_\_init\_\_()

|  |  |  |  |
| --- | --- | --- | --- |
| def RSEntry.RSEntry.\_\_init\_\_ | ( |  | *self*, |
|  |  |  | *chrom*, |
|  |  |  | *rsid*, |
|  |  |  | *pos* = `None` |
|  | ) |  |  |

```
Initialize an instance of the RSEntry class. Requires a chromosome and
a RSID number. Base position is optional, and if not passed will be
inferred from the first added variant.

Parameters:
chrom: Chromosome of the entry
rsid: RefSNP ID number of the entry
pos: Position of the entry
```

## Member Function Documentation

## ◆ \_\_add\_\_()

|  |  |  |  |
| --- | --- | --- | --- |
| def RSEntry.RSEntry.\_\_add\_\_ | ( |  | *self*, |
|  |  |  | *rse* |
|  | ) |  |  |

```
Implement addition for two RSEntry objects.
```

## ◆ \_\_eq\_\_()

|  |  |  |  |
| --- | --- | --- | --- |
| def RSEntry.RSEntry.\_\_eq\_\_ | ( |  | *self*, |
|  |  |  | *rse* |
|  | ) |  |  |

```
Implement equality checking for two RSEntry objects.
```

## ◆ \_\_getitem\_\_()

|  |  |  |  |
| --- | --- | --- | --- |
| def RSEntry.RSEntry.\_\_getitem\_\_ | ( |  | *self*, |
|  |  |  | *key* |
|  | ) |  |  |

```
Allow access to variants via the [] operator.
```

## ◆ \_\_iadd\_\_()

|  |  |  |  |
| --- | --- | --- | --- |
| def RSEntry.RSEntry.\_\_iadd\_\_ | ( |  | *self*, |
|  |  |  | *rse* |
|  | ) |  |  |

```
Implement incremental addition.
```

## ◆ \_\_len\_\_()

|  |  |  |  |  |  |
| --- | --- | --- | --- | --- | --- |
| def RSEntry.RSEntry.\_\_len\_\_ | ( |  | *self* | ) |  |

```
Implement len operator.
```

## ◆ \_\_repr\_\_()

|  |  |  |  |  |  |
| --- | --- | --- | --- | --- | --- |
| def RSEntry.RSEntry.\_\_repr\_\_ | ( |  | *self* | ) |  |

```
Implement repr operator.
```

## ◆ \_\_str\_\_()

|  |  |  |  |  |  |
| --- | --- | --- | --- | --- | --- |
| def RSEntry.RSEntry.\_\_str\_\_ | ( |  | *self* | ) |  |

```
Implement str operator.
```

## ◆ add\_var()

|  |  |  |  |
| --- | --- | --- | --- |
| def RSEntry.RSEntry.add\_var | ( |  | *self*, |
|  |  |  | *var* |
|  | ) |  |  |

```
Add a variant by passing an RSVariant object. Note that this creates a
new RSVar object and doesn't use the passed object.
    
Parameters:
var: RSVar object to add
```

## ◆ add\_var\_from\_args()

|  |  |  |  |
| --- | --- | --- | --- |
| def RSEntry.RSEntry.add\_var\_from\_args | ( |  | *self*, |
|  |  |  | *pos*, |
|  |  |  | *ref*, |
|  |  |  | *var*, |
|  |  |  | *major* = `0`, |
|  |  |  | *minor* = `0`, |
|  |  |  | *clin* = `[]`, |
|  |  |  | *afs* = `[]`, |
|  |  |  | *var\_type* = `''`, |
|  |  |  | *pop\_afs* = `{}` |
|  | ) |  |  |

```
Add a variant by passing all necessary information.

Parameters:
pos: Variant position
ref: Reference sequence at the variant position
var: Variant sequence
major: Number of studies in dbSNP listing this variant as major
minor: Number of studies in dbSNP listing this variant as minor
clin: Clinical significance of this variant
afs: Allele frequency of this allele
var_type: Variant type (SNP or indel) of this allele
pop_afs: Population allele frequencies
```

## ◆ all\_vars\_empty()

|  |  |  |  |  |  |
| --- | --- | --- | --- | --- | --- |
| def RSEntry.RSEntry.all\_vars\_empty | ( |  | *self* | ) |  |

```
Check if all variants are empty.
```

## ◆ get\_major\_alleles()

|  |  |  |  |  |  |
| --- | --- | --- | --- | --- | --- |
| def RSEntry.RSEntry.get\_major\_alleles | ( |  | *self* | ) |  |

```
Get a list of variants representing a major allele (AF >= 0.5).
```

## ◆ get\_major\_alleles\_pop()

|  |  |  |  |
| --- | --- | --- | --- |
| def RSEntry.RSEntry.get\_major\_alleles\_pop | ( |  | *self*, |
|  |  |  | *pop* |
|  | ) |  |  |

```
Get a list of variants representing a major allele (AF >= 0.5) for the
given population.

Parameters:
pop: Population to use
```

## ◆ pick\_major\_allele()

|  |  |  |  |
| --- | --- | --- | --- |
| def RSEntry.RSEntry.pick\_major\_allele | ( |  | *self*, |
|  |  |  | *var\_list* |
|  | ) |  |  |

```
Reproducibly choose a representative variant.

Parameters:
var_list: List of RSVar objects to pick from
```

## ◆ to\_vcf()

|  |  |  |  |
| --- | --- | --- | --- |
| def RSEntry.RSEntry.to\_vcf | ( |  | *self*, |
|  |  |  | *pop* = `None`, |
|  |  |  | *cons* = `False`, |
|  |  |  | *is\_maj* = `False` |
|  | ) |  |  |

```
Create a VCF record from variants. If preparing a consensus VCF
file, makes sure that there is only one alternate allele per line.

Parameters:
pop: Population to use
cons: If this VCF file is a consensus VCF
is_maj: If this RSCollection object already contains only major alleles
```

---

The documentation for this class was generated from the following file:

- RSEntry.py


---

Generated by  

 1.8.17
