# Supplementary material for: Pan-human consensus genome significantly improves the accuracy of RNA-seq analyses
Source: Genome Res. 2022 Apr;32(4):738–49. doi: 10.1101/gr.275613.121 (PMC8997357; doi:10.1101/gr.275613.121)
Supplement: Supplemental Material [file supp_gr.275613.121_Supplemental_Code.zip › Supplemental_Code/ConsDB/docs/classRSEntry_1_1RSEntry_1_1RSVar-members.html]

ConsDB: Member List


|  |
| --- |
| ConsDB  1.0  Tool for creating consensus genomes from variant databases. |


- **RSEntry**
- RSEntry
- RSVar

RSEntry.RSEntry.RSVar Member List

This is the complete list of members for RSEntry.RSEntry.RSVar, including all inherited members.

|  |  |  |
| --- | --- | --- |
| \_\_add\_\_(self, v) | RSEntry.RSEntry.RSVar |  |
| \_\_eq\_\_(self, v) | RSEntry.RSEntry.RSVar |  |
| \_\_iadd\_\_(self, v) | RSEntry.RSEntry.RSVar |  |
| \_\_init\_\_(self, pos, ref, var, major=0, minor=0, clin=[], afs=[], var\_type='', pop\_afs={}) | RSEntry.RSEntry.RSVar |  |
| \_\_repr\_\_(self) | RSEntry.RSEntry.RSVar |  |
| \_\_str\_\_(self) | RSEntry.RSEntry.RSVar |  |
| af(self) | RSEntry.RSEntry.RSVar |  |
| **afs** (defined in RSEntry.RSEntry.RSVar) | RSEntry.RSEntry.RSVar |  |
| calc\_afs(self) | RSEntry.RSEntry.RSVar |  |
| calc\_pop\_afs(self, pop) | RSEntry.RSEntry.RSVar |  |
| **clin** (defined in RSEntry.RSEntry.RSVar) | RSEntry.RSEntry.RSVar |  |
| is\_empty(self) | RSEntry.RSEntry.RSVar |  |
| is\_major(self) | RSEntry.RSEntry.RSVar |  |
| **major** (defined in RSEntry.RSEntry.RSVar) | RSEntry.RSEntry.RSVar |  |
| **minor** (defined in RSEntry.RSEntry.RSVar) | RSEntry.RSEntry.RSVar |  |
| pop\_af(self, pop) | RSEntry.RSEntry.RSVar |  |
| **pop\_afs** (defined in RSEntry.RSEntry.RSVar) | RSEntry.RSEntry.RSVar |  |
| **pos** (defined in RSEntry.RSEntry.RSVar) | RSEntry.RSEntry.RSVar |  |
| **ref** (defined in RSEntry.RSEntry.RSVar) | RSEntry.RSEntry.RSVar |  |
| **var** (defined in RSEntry.RSEntry.RSVar) | RSEntry.RSEntry.RSVar |  |
| var\_code(self) | RSEntry.RSEntry.RSVar |  |
| **var\_type** (defined in RSEntry.RSEntry.RSVar) | RSEntry.RSEntry.RSVar |  |


---

Generated by  

 1.8.17
