# Supplementary material for: Pan-human consensus genome significantly improves the accuracy of RNA-seq analyses
Source: Genome Res. 2022 Apr;32(4):738–49. doi: 10.1101/gr.275613.121 (PMC8997357; doi:10.1101/gr.275613.121)
Supplement: Supplemental Material [file supp_gr.275613.121_Supplemental_Code.zip › Supplemental_Code/ConsDB/docs/classRSEntry_1_1RSEntry_1_1RSVar.html]

ConsDB: RSEntry.RSEntry.RSVar Class Reference


|  |
| --- |
| ConsDB  1.0  Tool for creating consensus genomes from variant databases. |


- **RSEntry**
- RSEntry
- RSVar

Public Member Functions |
Public Attributes |
List of all members

RSEntry.RSEntry.RSVar Class Reference

|  |  |
| --- | --- |
| Public Member Functions | |
| def | \_\_init\_\_ (self, pos, ref, var, major=0, minor=0, clin=[], afs=[], var\_type='', pop\_afs={}) |
|  | |
| def | \_\_add\_\_ (self, v) |
|  | |
| def | \_\_eq\_\_ (self, v) |
|  | |
| def | \_\_iadd\_\_ (self, v) |
|  | |
| def | \_\_repr\_\_ (self) |
|  | |
| def | \_\_str\_\_ (self) |
|  | |
| def | af (self) |
|  | |
| def | calc\_afs (self) |
|  | |
| def | calc\_pop\_afs (self, pop) |
|  | |
| def | is\_major (self) |
|  | |
| def | is\_empty (self) |
|  | |
| def | pop\_af (self, pop) |
|  | |
| def | var\_code (self) |
|  | |

|  |  |
| --- | --- |
| Public Attributes | |
|  | **pos** |
|  | |
|  | **ref** |
|  | |
|  | **var** |
|  | |
|  | **major** |
|  | |
|  | **minor** |
|  | |
|  | **clin** |
|  | |
|  | **afs** |
|  | |
|  | **var\_type** |
|  | |
|  | **pop\_afs** |
|  | |

## Detailed Description

```
A sub-class to represent a variant.

Attributes
--------------------
pos : int
    Base position of the variant
ref : str
    Reference base(s)
var : str
    Variant base(s)
major : int
    Number of studies that show this variant with AF >= 0.5
minor : int
    Number of studies that show this variant with AF < 0.5
clin : list
    List of clinical relevances
afs : list
    List of allele frequencies, stores as tuples of 
    (# patients showing var, # patients in study)
var_type : str
    What type of variant (SNP, indel, or blank)
pop_afs : dict
    Dict containing population allele frequencies

Methods
--------------------
af()
    Get a representative allele frequency for the variant
calc_afs()
    Calculate float allele frequencies from afs list
calc_pop_afs(pop)
    Calculate float allele frequencies for the given population
is_major()
    Return if the variant represents a major allele
is_empty()
    Return if the variant has no studies supporting it
pop_af(pop)
    Get a representative population allele frequency for the variant
    and given population.
var_code()
    Get the representative variant code for the variant
```

## Constructor & Destructor Documentation

## ◆ \_\_init\_\_()

|  |  |  |  |
| --- | --- | --- | --- |
| def RSEntry.RSEntry.RSVar.\_\_init\_\_ | ( |  | *self*, |
|  |  |  | *pos*, |
|  |  |  | *ref*, |
|  |  |  | *var*, |
|  |  |  | *major* = `0`, |
|  |  |  | *minor* = `0`, |
|  |  |  | *clin* = `[]`, |
|  |  |  | *afs* = `[]`, |
|  |  |  | *var\_type* = `''`, |
|  |  |  | *pop\_afs* = `{}` |
|  | ) |  |  |

```
Initialize an instance of the RSVar class. Requires a position,
reference allele, and variant allele. All other information is 
oprtional, and will either be left blank or inferred (in the case
of var_type).

Parameters:
pos: Variant position
ref: Reference sequence at the variant position
var: Variant sequence
major: Number of studies in dbSNP listing this variant as major
minor: Number of studies in dbSNP listing this variant as minor
clin: Clinical significance of this variant
afs: Allele frequency of this allele
var_type: Variant type (SNP or indel) of this allele
pop_afs: Population allele frequencies
```

## Member Function Documentation

## ◆ \_\_add\_\_()

|  |  |  |  |
| --- | --- | --- | --- |
| def RSEntry.RSEntry.RSVar.\_\_add\_\_ | ( |  | *self*, |
|  |  |  | *v* |
|  | ) |  |  |

```
Implement addition for two RSVar objects.
```

## ◆ \_\_eq\_\_()

|  |  |  |  |
| --- | --- | --- | --- |
| def RSEntry.RSEntry.RSVar.\_\_eq\_\_ | ( |  | *self*, |
|  |  |  | *v* |
|  | ) |  |  |

```
Implement equality checking for two RSVar objects.
```

## ◆ \_\_iadd\_\_()

|  |  |  |  |
| --- | --- | --- | --- |
| def RSEntry.RSEntry.RSVar.\_\_iadd\_\_ | ( |  | *self*, |
|  |  |  | *v* |
|  | ) |  |  |

```
Implement incremental addition.
```

## ◆ \_\_repr\_\_()

|  |  |  |  |  |  |
| --- | --- | --- | --- | --- | --- |
| def RSEntry.RSEntry.RSVar.\_\_repr\_\_ | ( |  | *self* | ) |  |

```
Implement repr operator.
```

## ◆ \_\_str\_\_()

|  |  |  |  |  |  |
| --- | --- | --- | --- | --- | --- |
| def RSEntry.RSEntry.RSVar.\_\_str\_\_ | ( |  | *self* | ) |  |

```
Implement str operator.
```

## ◆ af()

|  |  |  |  |  |  |
| --- | --- | --- | --- | --- | --- |
| def RSEntry.RSEntry.RSVar.af | ( |  | *self* | ) |  |

```
Get a representative allele frequency for the variant.
```

## ◆ calc\_afs()

|  |  |  |  |  |  |
| --- | --- | --- | --- | --- | --- |
| def RSEntry.RSEntry.RSVar.calc\_afs | ( |  | *self* | ) |  |

```
Calculate float allele frequencies from afs list.
```

## ◆ calc\_pop\_afs()

|  |  |  |  |
| --- | --- | --- | --- |
| def RSEntry.RSEntry.RSVar.calc\_pop\_afs | ( |  | *self*, |
|  |  |  | *pop* |
|  | ) |  |  |

```
Calculate float allele frequencies from afs list.

Parameters:
pop: Population to use
```

## ◆ is\_empty()

|  |  |  |  |  |  |
| --- | --- | --- | --- | --- | --- |
| def RSEntry.RSEntry.RSVar.is\_empty | ( |  | *self* | ) |  |

```
Return if the variant has no studies supporting it.
```

## ◆ is\_major()

|  |  |  |  |  |  |
| --- | --- | --- | --- | --- | --- |
| def RSEntry.RSEntry.RSVar.is\_major | ( |  | *self* | ) |  |

```
Return if the variant represents a major allele.
```

## ◆ pop\_af()

|  |  |  |  |
| --- | --- | --- | --- |
| def RSEntry.RSEntry.RSVar.pop\_af | ( |  | *self*, |
|  |  |  | *pop* |
|  | ) |  |  |

```
Get a representative population allele frequency for the variant
and given population.

Parameters:
pop: Population to use
```

## ◆ var\_code()

|  |  |  |  |  |  |
| --- | --- | --- | --- | --- | --- |
| def RSEntry.RSEntry.RSVar.var\_code | ( |  | *self* | ) |  |

```
Get the representative variant code for the variant.
```

---

The documentation for this class was generated from the following file:

- RSEntry.py


---

Generated by  

 1.8.17
