# Supplementary material for: Pan-human consensus genome significantly improves the accuracy of RNA-seq analyses
Source: Genome Res. 2022 Apr;32(4):738–49. doi: 10.1101/gr.275613.121 (PMC8997357; doi:10.1101/gr.275613.121)
Supplement: Supplemental Material [file supp_gr.275613.121_Supplemental_Code.zip › Supplemental_Code/ConsDB/docs/classSlimRSCollection_1_1BitRSCollection-members.html]

ConsDB: Member List


|  |
| --- |
| ConsDB  1.0  Tool for creating consensus genomes from variant databases. |


- **SlimRSCollection**
- BitRSCollection

SlimRSCollection.BitRSCollection Member List

This is the complete list of members for SlimRSCollection.BitRSCollection, including all inherited members.

|  |  |  |
| --- | --- | --- |
| \_\_add\_\_(self, rsc) | SlimRSCollection.BitRSCollection |  |
| \_\_add\_\_(self, rsc) | SlimRSCollection.BitRSCollection |  |
| \_\_eq\_\_(self, rsc) | SlimRSCollection.BitRSCollection |  |
| \_\_eq\_\_(self, rsc) | SlimRSCollection.BitRSCollection |  |
| \_\_getitem\_\_(self, key) | SlimRSCollection.BitRSCollection |  |
| \_\_getitem\_\_(self, key) | SlimRSCollection.BitRSCollection |  |
| \_\_iadd\_\_(self, rsc) | SlimRSCollection.BitRSCollection |  |
| \_\_iadd\_\_(self, rsc) | SlimRSCollection.BitRSCollection |  |
| **\_\_init\_\_**(self) (defined in SlimRSCollection.BitRSCollection) | SlimRSCollection.BitRSCollection |  |
| **\_\_init\_\_**(self) (defined in SlimRSCollection.BitRSCollection) | SlimRSCollection.BitRSCollection |  |
| \_\_len\_\_(self) | SlimRSCollection.BitRSCollection |  |
| \_\_len\_\_(self) | SlimRSCollection.BitRSCollection |  |
| add\_entry(self, rsidx, e) | SlimRSCollection.BitRSCollection |  |
| add\_entry(self, rsidx, e) | SlimRSCollection.BitRSCollection |  |
| add\_entry\_from\_args(self, chrom, rsid, pos, silent=False) | SlimRSCollection.BitRSCollection |  |
| add\_entry\_from\_args(self, chrom, rsid, pos, quiet=False) | SlimRSCollection.BitRSCollection |  |
| add\_entry\_line(self, e) | SlimRSCollection.BitRSCollection |  |
| add\_entry\_line(self, e) | SlimRSCollection.BitRSCollection |  |
| **BASE\_ENC** (defined in SlimRSCollection.BitRSCollection) | SlimRSCollection.BitRSCollection | static |
| **chr\_pos\_table** (defined in SlimRSCollection.BitRSCollection) | SlimRSCollection.BitRSCollection |  |
| chrom\_to\_int(c) | SlimRSCollection.BitRSCollection | static |
| decode\_bit(bit\_code) | SlimRSCollection.BitRSCollection | static |
| decode\_bit(bit\_code) | SlimRSCollection.BitRSCollection | static |
| dump\_vcf(self, fn, cons=False, is\_maj=False, append=False) | SlimRSCollection.BitRSCollection |  |
| encode\_bit(var\_list) | SlimRSCollection.BitRSCollection | static |
| encode\_bit(var\_list) | SlimRSCollection.BitRSCollection | static |
| **entries** (defined in SlimRSCollection.BitRSCollection) | SlimRSCollection.BitRSCollection |  |
| filter\_vcf(rsc\_dir, fn\_in, fn\_out, pop=None, log\_fn=None, quiet=False) | SlimRSCollection.BitRSCollection | static |
| filter\_vcf(rsc\_dir, fn\_in, fn\_out, pop=None, log\_fn=None, cons=False, keep\_samps=False, quiet=False) | SlimRSCollection.BitRSCollection | static |
| get\_by\_chr\_pos(self, chrom, pos) | SlimRSCollection.BitRSCollection |  |
| get\_by\_chr\_pos(self, chrom, pos) | SlimRSCollection.BitRSCollection |  |
| get\_by\_rsid(self, rsid) | SlimRSCollection.BitRSCollection |  |
| get\_by\_rsid(self, rsid) | SlimRSCollection.BitRSCollection |  |
| get\_chrom\_from\_filename(fn) | SlimRSCollection.BitRSCollection | static |
| get\_chrom\_from\_filename(fn) | SlimRSCollection.BitRSCollection | static |
| get\_major(self, mut=True) | SlimRSCollection.BitRSCollection |  |
| get\_major(self, mut=True) | SlimRSCollection.BitRSCollection |  |
| load\_from\_file\_full(fn, quiet=False) | SlimRSCollection.BitRSCollection | static |
| load\_from\_file\_full(fn, quiet=False) | SlimRSCollection.BitRSCollection | static |
| load\_from\_file\_pop(fn, pop, quiet=False) | SlimRSCollection.BitRSCollection | static |
| load\_from\_file\_pop(fn, pop, quiet=False) | SlimRSCollection.BitRSCollection | static |
| open(fn) | SlimRSCollection.BitRSCollection | static |
| open(fn) | SlimRSCollection.BitRSCollection | static |
| **rsid\_table** (defined in SlimRSCollection.BitRSCollection) | SlimRSCollection.BitRSCollection |  |
| sort\_rsidx(rsidx) | SlimRSCollection.BitRSCollection | static |
| var\_list\_to\_vcf(rsidx, var\_list, cons=False) | SlimRSCollection.BitRSCollection | static |


---

Generated by  

 1.8.17
