# Supplementary material for: Pan-human consensus genome significantly improves the accuracy of RNA-seq analyses
Source: Genome Res. 2022 Apr;32(4):738–49. doi: 10.1101/gr.275613.121 (PMC8997357; doi:10.1101/gr.275613.121)
Supplement: Supplemental Material [file supp_gr.275613.121_Supplemental_Code.zip › Supplemental_Code/ConsDB/docs/classSlimRSCollection_1_1BitRSCollection.html]

ConsDB: SlimRSCollection.BitRSCollection Class Reference


|  |
| --- |
| ConsDB  1.0  Tool for creating consensus genomes from variant databases. |


- **SlimRSCollection**
- BitRSCollection

Public Member Functions |
Static Public Member Functions |
Public Attributes |
Static Public Attributes |
List of all members

SlimRSCollection.BitRSCollection Class Reference

|  |  |
| --- | --- |
| Public Member Functions | |
| def | **\_\_init\_\_** (self) |
|  | |
| def | \_\_add\_\_ (self, rsc) |
|  | |
| def | \_\_eq\_\_ (self, rsc) |
|  | |
| def | \_\_getitem\_\_ (self, key) |
|  | |
| def | \_\_iadd\_\_ (self, rsc) |
|  | |
| def | \_\_len\_\_ (self) |
|  | |
| def | add\_entry (self, rsidx, e) |
|  | |
| def | add\_entry\_line (self, e) |
|  | |
| def | add\_entry\_from\_args (self, chrom, rsid, pos, silent=False) |
|  | |
| def | get\_by\_chr\_pos (self, chrom, pos) |
|  | |
| def | get\_by\_rsid (self, rsid) |
|  | |
| def | get\_major (self, mut=True) |
|  | |
| def | **\_\_init\_\_** (self) |
|  | |
| def | \_\_add\_\_ (self, rsc) |
|  | |
| def | \_\_eq\_\_ (self, rsc) |
|  | |
| def | \_\_getitem\_\_ (self, key) |
|  | |
| def | \_\_iadd\_\_ (self, rsc) |
|  | |
| def | \_\_len\_\_ (self) |
|  | |
| def | add\_entry (self, rsidx, e) |
|  | |
| def | add\_entry\_line (self, e) |
|  | |
| def | add\_entry\_from\_args (self, chrom, rsid, pos, quiet=False) |
|  | |
| def | dump\_vcf (self, fn, cons=False, is\_maj=False, append=False) |
|  | |
| def | get\_by\_chr\_pos (self, chrom, pos) |
|  | |
| def | get\_by\_rsid (self, rsid) |
|  | |
| def | get\_major (self, mut=True) |
|  | |

|  |  |
| --- | --- |
| Static Public Member Functions | |
| def | decode\_bit (bit\_code) |
|  | |
| def | encode\_bit (var\_list) |
|  | |
| def | filter\_vcf (rsc\_dir, fn\_in, fn\_out, pop=None, log\_fn=None, quiet=False) |
|  | |
| def | get\_chrom\_from\_filename (fn) |
|  | |
| def | load\_from\_file\_full (fn, quiet=False) |
|  | |
| def | load\_from\_file\_pop (fn, pop, quiet=False) |
|  | |
| def | open (fn) |
|  | |
| def | chrom\_to\_int (c) |
|  | |
| def | decode\_bit (bit\_code) |
|  | |
| def | encode\_bit (var\_list) |
|  | |
| def | filter\_vcf (rsc\_dir, fn\_in, fn\_out, pop=None, log\_fn=None, cons=False, keep\_samps=False, quiet=False) |
|  | |
| def | get\_chrom\_from\_filename (fn) |
|  | |
| def | load\_from\_file\_full (fn, quiet=False) |
|  | |
| def | load\_from\_file\_pop (fn, pop, quiet=False) |
|  | |
| def | open (fn) |
|  | |
| def | sort\_rsidx (rsidx) |
|  | |
| def | var\_list\_to\_vcf (rsidx, var\_list, cons=False) |
|  | |

|  |  |
| --- | --- |
| Public Attributes | |
|  | **entries** |
|  | |
|  | **chr\_pos\_table** |
|  | |
|  | **rsid\_table** |
|  | |

|  |  |
| --- | --- |
| Static Public Attributes | |
| dictionary | **BASE\_ENC** |
|  | |

## Member Function Documentation

## ◆ \_\_add\_\_() [1/2]

|  |  |  |  |
| --- | --- | --- | --- |
| def SlimRSCollection.BitRSCollection.\_\_add\_\_ | ( |  | *self*, |
|  |  |  | *rsc* |
|  | ) |  |  |

```
Implement addition for two RSCollection objects.
```

## ◆ \_\_add\_\_() [2/2]

|  |  |  |  |
| --- | --- | --- | --- |
| def SlimRSCollection.BitRSCollection.\_\_add\_\_ | ( |  | *self*, |
|  |  |  | *rsc* |
|  | ) |  |  |

```
Implement addition for two RSCollection objects.
```

## ◆ \_\_eq\_\_() [1/2]

|  |  |  |  |
| --- | --- | --- | --- |
| def SlimRSCollection.BitRSCollection.\_\_eq\_\_ | ( |  | *self*, |
|  |  |  | *rsc* |
|  | ) |  |  |

```
Implement equality checking for two RSCollection objects.
```

## ◆ \_\_eq\_\_() [2/2]

|  |  |  |  |
| --- | --- | --- | --- |
| def SlimRSCollection.BitRSCollection.\_\_eq\_\_ | ( |  | *self*, |
|  |  |  | *rsc* |
|  | ) |  |  |

```
Implement equality checking for two RSCollection objects.
```

## ◆ \_\_getitem\_\_() [1/2]

|  |  |  |  |
| --- | --- | --- | --- |
| def SlimRSCollection.BitRSCollection.\_\_getitem\_\_ | ( |  | *self*, |
|  |  |  | *key* |
|  | ) |  |  |

```
Allow access to entries via the [] operator.
```

## ◆ \_\_getitem\_\_() [2/2]

|  |  |  |  |
| --- | --- | --- | --- |
| def SlimRSCollection.BitRSCollection.\_\_getitem\_\_ | ( |  | *self*, |
|  |  |  | *key* |
|  | ) |  |  |

```
Allow access to entries via the [] operator.
```

## ◆ \_\_iadd\_\_() [1/2]

|  |  |  |  |
| --- | --- | --- | --- |
| def SlimRSCollection.BitRSCollection.\_\_iadd\_\_ | ( |  | *self*, |
|  |  |  | *rsc* |
|  | ) |  |  |

```
Implement incremental addition.
```

## ◆ \_\_iadd\_\_() [2/2]

|  |  |  |  |
| --- | --- | --- | --- |
| def SlimRSCollection.BitRSCollection.\_\_iadd\_\_ | ( |  | *self*, |
|  |  |  | *rsc* |
|  | ) |  |  |

```
Implement incremental addition.
```

## ◆ \_\_len\_\_() [1/2]

|  |  |  |  |  |  |
| --- | --- | --- | --- | --- | --- |
| def SlimRSCollection.BitRSCollection.\_\_len\_\_ | ( |  | *self* | ) |  |

```
Implement len operator.
```

## ◆ \_\_len\_\_() [2/2]

|  |  |  |  |  |  |
| --- | --- | --- | --- | --- | --- |
| def SlimRSCollection.BitRSCollection.\_\_len\_\_ | ( |  | *self* | ) |  |

```
Implement len operator.
```

## ◆ add\_entry() [1/2]

|  |  |  |  |
| --- | --- | --- | --- |
| def SlimRSCollection.BitRSCollection.add\_entry | ( |  | *self*, |
|  |  |  | *rsidx*, |
|  |  |  | *e* |
|  | ) |  |  |

```
Add entry from a variant list.

Parameters:
rsidx: Tuple of (rsid, chrom, pos)
e: List of variants (output from decode_bit)
```

## ◆ add\_entry() [2/2]

|  |  |  |  |
| --- | --- | --- | --- |
| def SlimRSCollection.BitRSCollection.add\_entry | ( |  | *self*, |
|  |  |  | *rsidx*, |
|  |  |  | *e* |
|  | ) |  |  |

```
Add entry from a variant list.

Parameters:
rsidx: Tuple of (rsid, chrom, pos)
e: List of variants (output from decode_bit)
```

## ◆ add\_entry\_from\_args() [1/2]

|  |  |  |  |
| --- | --- | --- | --- |
| def SlimRSCollection.BitRSCollection.add\_entry\_from\_args | ( |  | *self*, |
|  |  |  | *chrom*, |
|  |  |  | *rsid*, |
|  |  |  | *pos*, |
|  |  |  | *quiet* = `False` |
|  | ) |  |  |

```
Add an entry based on the given arguments.

Parameters:
chrom: Chromosome of the entry being added
rsid: RefSNP ID of the entry being added
pos: Position of the entry being added
quiet: Suppress progress information being printed
```

## ◆ add\_entry\_from\_args() [2/2]

|  |  |  |  |
| --- | --- | --- | --- |
| def SlimRSCollection.BitRSCollection.add\_entry\_from\_args | ( |  | *self*, |
|  |  |  | *chrom*, |
|  |  |  | *rsid*, |
|  |  |  | *pos*, |
|  |  |  | *silent* = `False` |
|  | ) |  |  |

```
Add an entry based on the given arguments.

Parameters:
chrom: Chromosome of the entry being added
rsid: RefSNP ID of the entry being added
pos: Position of the entry being added
silent: Whether to suppress progress information being printed
```

## ◆ add\_entry\_line() [1/2]

|  |  |  |  |
| --- | --- | --- | --- |
| def SlimRSCollection.BitRSCollection.add\_entry\_line | ( |  | *self*, |
|  |  |  | *e* |
|  | ) |  |  |

```
Add entry from a line in a ConsDB file.

Parameters:
e: Line from a ConsDB file containing the entry to add
```

## ◆ add\_entry\_line() [2/2]

|  |  |  |  |
| --- | --- | --- | --- |
| def SlimRSCollection.BitRSCollection.add\_entry\_line | ( |  | *self*, |
|  |  |  | *e* |
|  | ) |  |  |

```
Add entry from a line in a ConsDB file.

Parameters:
e: Line from a ConsDB file containing the entry to add
```

## ◆ chrom\_to\_int()

|  |  |  |  |  |  |  |  |
| --- | --- | --- | --- | --- | --- | --- | --- |
| |  |  |  |  |  |  | | --- | --- | --- | --- | --- | --- | | def SlimRSCollection.BitRSCollection.chrom\_to\_int | ( |  | *c* | ) |  | | static |

```
Function to use to sort chromosomes (numeric < X < Y < M)

Parameters:
c: Chromosome to convert
```

## ◆ decode\_bit() [1/2]

|  |  |  |  |  |  |  |  |
| --- | --- | --- | --- | --- | --- | --- | --- |
| |  |  |  |  |  |  | | --- | --- | --- | --- | --- | --- | | def SlimRSCollection.BitRSCollection.decode\_bit | ( |  | *bit\_code* | ) |  | | static |

```
Convert from bit-packed int to list of variants.

Parameters:
bit_code: int that can be unpacked into a list of variants
```

## ◆ decode\_bit() [2/2]

|  |  |  |  |  |  |  |  |
| --- | --- | --- | --- | --- | --- | --- | --- |
| |  |  |  |  |  |  | | --- | --- | --- | --- | --- | --- | | def SlimRSCollection.BitRSCollection.decode\_bit | ( |  | *bit\_code* | ) |  | | static |

```
Convert from bit-packed int to list of variants.

Parameters:
bit_code: int that can be unpacked into a list of variants
```

## ◆ dump\_vcf()

|  |  |  |  |
| --- | --- | --- | --- |
| def SlimRSCollection.BitRSCollection.dump\_vcf | ( |  | *self*, |
|  |  |  | *fn*, |
|  |  |  | *cons* = `False`, |
|  |  |  | *is\_maj* = `False`, |
|  |  |  | *append* = `False` |
|  | ) |  |  |

```
Create a VCF file containing the variants in the BitRSCollection object.
If saving a consensus VCF file, makes sure that there is only one
alternate allele per line. The append argument allows support for
loading/saving in a piecewise fashion.

Parameters:
fn: File to save VCF to
cons: If this VCF file is a consensus VCF
is_maj: If this RSCollection object already contains only major alleles
append: If appending to an existing VCF file
```

## ◆ encode\_bit() [1/2]

|  |  |  |  |  |  |  |  |
| --- | --- | --- | --- | --- | --- | --- | --- |
| |  |  |  |  |  |  | | --- | --- | --- | --- | --- | --- | | def SlimRSCollection.BitRSCollection.encode\_bit | ( |  | *var\_list* | ) |  | | static |

```
Create a bit-packed int from a list of variants.

Parameters:
var_list: List of variants
```

## ◆ encode\_bit() [2/2]

|  |  |  |  |  |  |  |  |
| --- | --- | --- | --- | --- | --- | --- | --- |
| |  |  |  |  |  |  | | --- | --- | --- | --- | --- | --- | | def SlimRSCollection.BitRSCollection.encode\_bit | ( |  | *var\_list* | ) |  | | static |

```
Create a bit-packed int from a list of variants.

Parameters:
var_list: List of variants
```

## ◆ filter\_vcf() [1/2]

|  |  |  |  |  |  |  |  |  |  |  |  |  |  |  |  |  |  |  |  |  |  |  |  |  |  |  |  |  |  |  |  |  |  |  |  |  |  |
| --- | --- | --- | --- | --- | --- | --- | --- | --- | --- | --- | --- | --- | --- | --- | --- | --- | --- | --- | --- | --- | --- | --- | --- | --- | --- | --- | --- | --- | --- | --- | --- | --- | --- | --- | --- | --- | --- |
| |  |  |  |  | | --- | --- | --- | --- | | def SlimRSCollection.BitRSCollection.filter\_vcf | ( |  | *rsc\_dir*, | |  |  |  | *fn\_in*, | |  |  |  | *fn\_out*, | |  |  |  | *pop* = `None`, | |  |  |  | *log\_fn* = `None`, | |  |  |  | *cons* = `False`, | |  |  |  | *keep\_samps* = `False`, | |  |  |  | *quiet* = `False` | |  | ) |  |  | | static |

```
Filter a given VCF file using ConsDB files stored in the given dir.

Remove all records that call a variant that is a major allele and write
all other records.

Parameters:
rsc_dir: Directory containing ConsDB files
fn_in: Input VCF file to filter
fn_out: Output filename
pop: Population to use
log_fn: Optional file to store progress/log output
cons: Making a consensus VCF (keep major alleles instead of minor)
keep_samps: Write sample information
quiet: Disable progress/log output to stdout
```

## ◆ filter\_vcf() [2/2]

|  |  |  |  |  |  |  |  |  |  |  |  |  |  |  |  |  |  |  |  |  |  |  |  |  |  |  |  |  |  |
| --- | --- | --- | --- | --- | --- | --- | --- | --- | --- | --- | --- | --- | --- | --- | --- | --- | --- | --- | --- | --- | --- | --- | --- | --- | --- | --- | --- | --- | --- |
| |  |  |  |  | | --- | --- | --- | --- | | def SlimRSCollection.BitRSCollection.filter\_vcf | ( |  | *rsc\_dir*, | |  |  |  | *fn\_in*, | |  |  |  | *fn\_out*, | |  |  |  | *pop* = `None`, | |  |  |  | *log\_fn* = `None`, | |  |  |  | *quiet* = `False` | |  | ) |  |  | | static |

```
Filter a given VCF file using ConsDB files stored in the given dir.

Remove all records that call a variant that is a major allele and write
all other records.

Parameters:
rsc_dir: Directory containing ConsDB files
fn_in: Input VCF file to filter
fn_out: Output filename
pop: Population to use
log_fn: Optional file to store progress/log output
quiet: Disable progress/log output to stdout
```

## ◆ get\_by\_chr\_pos() [1/2]

|  |  |  |  |
| --- | --- | --- | --- |
| def SlimRSCollection.BitRSCollection.get\_by\_chr\_pos | ( |  | *self*, |
|  |  |  | *chrom*, |
|  |  |  | *pos* |
|  | ) |  |  |

```
Return a list of all (rsid, chrom, pos) combinations that match the
given chrom and pos, and a list of corresponding entries.

Parameters:
chrom: Chromosome to get
pos: Position to get
```

## ◆ get\_by\_chr\_pos() [2/2]

|  |  |  |  |
| --- | --- | --- | --- |
| def SlimRSCollection.BitRSCollection.get\_by\_chr\_pos | ( |  | *self*, |
|  |  |  | *chrom*, |
|  |  |  | *pos* |
|  | ) |  |  |

```
Return a list of all (rsid, chrom, pos) combinations that match the
given chrom and position, and a list of corresponding entries.

Parameters:
chrom: Chromosome to get
pos: Position to get
```

## ◆ get\_by\_rsid() [1/2]

|  |  |  |  |
| --- | --- | --- | --- |
| def SlimRSCollection.BitRSCollection.get\_by\_rsid | ( |  | *self*, |
|  |  |  | *rsid* |
|  | ) |  |  |

```
Return a list of all (rsid, chrom, pos) combinations that match the
given RefSNP ID, and a list of corresponding entries.

Parameters:
rsid: RefSNP ID number to get
```

## ◆ get\_by\_rsid() [2/2]

|  |  |  |  |
| --- | --- | --- | --- |
| def SlimRSCollection.BitRSCollection.get\_by\_rsid | ( |  | *self*, |
|  |  |  | *rsid* |
|  | ) |  |  |

```
Return a list of all (rsid, chrom, pos) combinations that match the
given RefSNP ID, and a list of corresponding entries.

Parameters:
rsid: RefSNP ID number to get
```

## ◆ get\_chrom\_from\_filename() [1/2]

|  |  |  |  |  |  |  |  |
| --- | --- | --- | --- | --- | --- | --- | --- |
| |  |  |  |  |  |  | | --- | --- | --- | --- | --- | --- | | def SlimRSCollection.BitRSCollection.get\_chrom\_from\_filename | ( |  | *fn* | ) |  | | static |

```
Helper method to parse a filename and find which chromosome it is.

Parameters:
fn: Filename to use
```

## ◆ get\_chrom\_from\_filename() [2/2]

|  |  |  |  |  |  |  |  |
| --- | --- | --- | --- | --- | --- | --- | --- |
| |  |  |  |  |  |  | | --- | --- | --- | --- | --- | --- | | def SlimRSCollection.BitRSCollection.get\_chrom\_from\_filename | ( |  | *fn* | ) |  | | static |

```
Helper method to parse a filename and find which chromosome it is.

Parameters:
fn: Filename to use
```

## ◆ get\_major() [1/2]

|  |  |  |  |
| --- | --- | --- | --- |
| def SlimRSCollection.BitRSCollection.get\_major | ( |  | *self*, |
|  |  |  | *mut* = `True` |
|  | ) |  |  |

```
Return a RSCollection object containing all major alleles.

Parameters:
mut: Whether to only include variants that are different between
    reference and alternate allele
```

## ◆ get\_major() [2/2]

|  |  |  |  |
| --- | --- | --- | --- |
| def SlimRSCollection.BitRSCollection.get\_major | ( |  | *self*, |
|  |  |  | *mut* = `True` |
|  | ) |  |  |

```
Return a BitRSCollection object containing all major alleles.

Parameters:
mut: Whether to only include variants that are different between
    reference and alternate allele
```

## ◆ load\_from\_file\_full() [1/2]

|  |  |  |  |  |  |  |  |  |  |  |  |  |  |
| --- | --- | --- | --- | --- | --- | --- | --- | --- | --- | --- | --- | --- | --- |
| |  |  |  |  | | --- | --- | --- | --- | | def SlimRSCollection.BitRSCollection.load\_from\_file\_full | ( |  | *fn*, | |  |  |  | *quiet* = `False` | |  | ) |  |  | | static |

```
Load an entire ConsDB file.

Parameters:
fn: Filename to load
quiet: Disable progress/log output to stdout
```

## ◆ load\_from\_file\_full() [2/2]

|  |  |  |  |  |  |  |  |  |  |  |  |  |  |
| --- | --- | --- | --- | --- | --- | --- | --- | --- | --- | --- | --- | --- | --- |
| |  |  |  |  | | --- | --- | --- | --- | | def SlimRSCollection.BitRSCollection.load\_from\_file\_full | ( |  | *fn*, | |  |  |  | *quiet* = `False` | |  | ) |  |  | | static |

```
Load an entire ConsDB file.

Parameters:
fn: Filename to load
quiet: Disable progress/log output to stdout
```

## ◆ load\_from\_file\_pop() [1/2]

|  |  |  |  |  |  |  |  |  |  |  |  |  |  |  |  |  |  |
| --- | --- | --- | --- | --- | --- | --- | --- | --- | --- | --- | --- | --- | --- | --- | --- | --- | --- |
| |  |  |  |  | | --- | --- | --- | --- | | def SlimRSCollection.BitRSCollection.load\_from\_file\_pop | ( |  | *fn*, | |  |  |  | *pop*, | |  |  |  | *quiet* = `False` | |  | ) |  |  | | static |

```
Load an entire ConsDB file, setting the allele frequency as the AF of
the given population.
Skip variants that are not present in the given population.

Parameters:
fn: Filename to use
pop: Population to load
quiet: Disable progress/log output to stdout
```

## ◆ load\_from\_file\_pop() [2/2]

|  |  |  |  |  |  |  |  |  |  |  |  |  |  |  |  |  |  |
| --- | --- | --- | --- | --- | --- | --- | --- | --- | --- | --- | --- | --- | --- | --- | --- | --- | --- |
| |  |  |  |  | | --- | --- | --- | --- | | def SlimRSCollection.BitRSCollection.load\_from\_file\_pop | ( |  | *fn*, | |  |  |  | *pop*, | |  |  |  | *quiet* = `False` | |  | ) |  |  | | static |

```
Load an entire ConsDB file, setting the allele frequency as the AF of
the given population.
Skip variants that are not present in the given population.

Parameters:
fn: Filename to use
pop: Population to load
quiet: Disable progress/log output to stdout
```

## ◆ open() [1/2]

|  |  |  |  |  |  |  |  |
| --- | --- | --- | --- | --- | --- | --- | --- |
| |  |  |  |  |  |  | | --- | --- | --- | --- | --- | --- | | def SlimRSCollection.BitRSCollection.open | ( |  | *fn* | ) |  | | static |

```
Helper method to appropriately open the given file.
```

## ◆ open() [2/2]

|  |  |  |  |  |  |  |  |
| --- | --- | --- | --- | --- | --- | --- | --- |
| |  |  |  |  |  |  | | --- | --- | --- | --- | --- | --- | | def SlimRSCollection.BitRSCollection.open | ( |  | *fn* | ) |  | | static |

```
Helper method to appropriately open the given file.

Parameters:
fn: Filename to open
```

## ◆ sort\_rsidx()

|  |  |  |  |  |  |  |  |
| --- | --- | --- | --- | --- | --- | --- | --- |
| |  |  |  |  |  |  | | --- | --- | --- | --- | --- | --- | | def SlimRSCollection.BitRSCollection.sort\_rsidx | ( |  | *rsidx* | ) |  | | static |

```
Helper method to sort a list of rsidx. Meant to be used as a
key for sorting. Returns the chromosome and position of the rsidx, both
in int form.

Parameters:
rsidx: List/tuple of (rsid, chrom, pos)
```

## ◆ var\_list\_to\_vcf()

|  |  |  |  |  |  |  |  |  |  |  |  |  |  |  |  |  |  |
| --- | --- | --- | --- | --- | --- | --- | --- | --- | --- | --- | --- | --- | --- | --- | --- | --- | --- |
| |  |  |  |  | | --- | --- | --- | --- | | def SlimRSCollection.BitRSCollection.var\_list\_to\_vcf | ( |  | *rsidx*, | |  |  |  | *var\_list*, | |  |  |  | *cons* = `False` | |  | ) |  |  | | static |

```
Helper method to convert a list of variants to vcf entry(ies).

Parameters:
rsidx: List/tuple of (rsid, chrom, pos)
var_list: List of variants (as returned from BitRSCollection.decode_bit)
cons: Making a consensus VCF file
```

## Member Data Documentation

## ◆ BASE\_ENC

|  |  |  |
| --- | --- | --- |
| |  | | --- | | dictionary SlimRSCollection.BitRSCollection.BASE\_ENC | | static |

**Initial value:**

= {

'A': 0,

'C': 1,

'G': 2,

'T': 3

}

---

The documentation for this class was generated from the following files:

- SlimRSCollection.old.py
- SlimRSCollection.py


---

Generated by  

 1.8.17
