# Supplementary material for: Pan-human consensus genome significantly improves the accuracy of RNA-seq analyses
Source: Genome Res. 2022 Apr;32(4):738–49. doi: 10.1101/gr.275613.121 (PMC8997357; doi:10.1101/gr.275613.121)
Supplement: Supplemental Material [file supp_gr.275613.121_Supplemental_Code.zip › Supplemental_Code/ConsDB/docs/functions.html]

ConsDB: Class Members


|  |
| --- |
| ConsDB  1.0  Tool for creating consensus genomes from variant databases. |


Here is a list of all documented class members with links to the class documentation for each member:

### - \_ -

- \_\_add\_\_()
  : RSEntry.RSCollection
  , RSEntry.RSEntry
  , RSEntry.RSEntry.RSVar
  , SlimRSCollection.BitRSCollection
- \_\_eq\_\_()
  : RSEntry.RSCollection
  , RSEntry.RSEntry
  , RSEntry.RSEntry.RSVar
  , SlimRSCollection.BitRSCollection
- \_\_getitem\_\_()
  : RSEntry.RSCollection
  , RSEntry.RSEntry
  , SlimRSCollection.BitRSCollection
- \_\_iadd\_\_()
  : RSEntry.RSCollection
  , RSEntry.RSEntry
  , RSEntry.RSEntry.RSVar
  , SlimRSCollection.BitRSCollection
- \_\_init\_\_()
  : RSEntry.RSEntry
  , RSEntry.RSEntry.RSVar
- \_\_len\_\_()
  : RSEntry.RSCollection
  , RSEntry.RSEntry
  , SlimRSCollection.BitRSCollection
- \_\_repr\_\_()
  : RSEntry.RSCollection
  , RSEntry.RSEntry
  , RSEntry.RSEntry.RSVar
- \_\_str\_\_()
  : RSEntry.RSCollection
  , RSEntry.RSEntry
  , RSEntry.RSEntry.RSVar

### - a -

- add\_entry()
  : RSEntry.RSCollection
  , SlimRSCollection.BitRSCollection
- add\_entry\_from\_args()
  : RSEntry.RSCollection
  , SlimRSCollection.BitRSCollection
- add\_entry\_line()
  : SlimRSCollection.BitRSCollection
- add\_var()
  : RSEntry.RSEntry
- add\_var\_from\_args()
  : RSEntry.RSEntry
- af()
  : RSEntry.RSEntry.RSVar
- all\_vars\_empty()
  : RSEntry.RSEntry

### - c -

- calc\_afs()
  : RSEntry.RSEntry.RSVar
- calc\_pop\_afs()
  : RSEntry.RSEntry.RSVar
- chrom\_to\_int()
  : RSEntry.RSCollection
  , SlimRSCollection.BitRSCollection

### - d -

- decode\_bit()
  : SlimRSCollection.BitRSCollection
- dump()
  : RSEntry.RSCollection
- dump\_chrs()
  : RSEntry.RSCollection
- dump\_full()
  : RSEntry.RSCollection
- dump\_vcf()
  : RSEntry.RSCollection
  , SlimRSCollection.BitRSCollection

### - e -

- encode\_bit()
  : SlimRSCollection.BitRSCollection

### - f -

- filter\_vcf()
  : SlimRSCollection.BitRSCollection
- from\_1000gp()
  : RSEntry.RSCollection
- from\_dbsnp()
  : RSEntry.RSCollection
- from\_gnomad()
  : RSEntry.RSCollection

### - g -

- get\_by\_chr()
  : RSEntry.RSCollection
- get\_by\_chr\_pos()
  : RSEntry.RSCollection
  , SlimRSCollection.BitRSCollection
- get\_by\_rsid()
  : RSEntry.RSCollection
  , SlimRSCollection.BitRSCollection
- get\_chrom\_from\_filename()
  : RSEntry.RSCollection
  , SlimRSCollection.BitRSCollection
- get\_major()
  : RSEntry.RSCollection
  , SlimRSCollection.BitRSCollection
- get\_major\_alleles()
  : RSEntry.RSEntry
- get\_major\_alleles\_pop()
  : RSEntry.RSEntry

### - i -

- is\_empty()
  : RSEntry.RSEntry.RSVar
- is\_major()
  : RSEntry.RSEntry.RSVar

### - l -

- load\_from\_file\_by\_chr\_pos()
  : RSEntry.RSCollection
- load\_from\_file\_by\_rsid()
  : RSEntry.RSCollection
- load\_from\_file\_full()
  : RSEntry.RSCollection
  , SlimRSCollection.BitRSCollection
- load\_from\_file\_pop()
  : SlimRSCollection.BitRSCollection
- load\_from\_file\_pops()
  : RSEntry.RSCollection

### - m -

- make\_chunk\_idx\_dict()
  : RSEntry.RSCollection
- merge\_files()
  : RSEntry.RSCollection

### - o -

- open()
  : RSEntry.RSCollection
  , SlimRSCollection.BitRSCollection

### - p -

- parse\_file\_line()
  : RSEntry.RSCollection
- pick\_major\_allele()
  : RSEntry.RSEntry
- pop\_af()
  : RSEntry.RSEntry.RSVar

### - s -

- sort\_rsidx()
  : RSEntry.RSCollection
  , SlimRSCollection.BitRSCollection
- sort\_rsidx\_line()
  : RSEntry.RSCollection

### - t -

- to\_vcf()
  : RSEntry.RSEntry

### - v -

- var\_code()
  : RSEntry.RSEntry.RSVar
- var\_list\_to\_vcf()
  : SlimRSCollection.BitRSCollection


---

Generated by  

 1.8.17
