# Supplementary material for: Pan-human consensus genome significantly improves the accuracy of RNA-seq analyses
Source: Genome Res. 2022 Apr;32(4):738–49. doi: 10.1101/gr.275613.121 (PMC8997357; doi:10.1101/gr.275613.121)
Supplement: Supplemental Material [file supp_gr.275613.121_Supplemental_Code.zip › Supplemental_Code/ConsDB/docs/md_README.html]

ConsDB: ConsDB


|  |
| --- |
| ConsDB  1.0  Tool for creating consensus genomes from variant databases. |

ConsDB

A Python tool for interfacing with large variant databases and performing consensus genome operations.

Written by Benjamin Kaminow

# Usage

The main ConsDB script can be run as follows:

```
cd consdb
python ConsDB.py <run mode> [arguments]
```

Where `<run mode>` is one of the following:

- `Parse` - Parse database files into ConsDB files
- `Filter` - Filter a VCF file to remove major alleles
- `Merge` - Merge multiple ConsDB files
- `Cons` - Create a consensus VCF file
- `FA` - Create a consensus FASTA file

Arguments for each run mode can be viewed by passing `-h` as the sole argument.

# Back-End

ConsDB utilizes an object oriented back-end, which can easily be incorporated into pipeline/scripts. Documentation for this back-end is found in the `docs` subfolder.


---

Generated by  

 1.8.17
