# Supplementary material for: Pan-human consensus genome significantly improves the accuracy of RNA-seq analyses
Source: Genome Res. 2022 Apr;32(4):738–49. doi: 10.1101/gr.275613.121 (PMC8997357; doi:10.1101/gr.275613.121)
Supplement: Supplemental Material [file supp_gr.275613.121_Supplemental_Code.zip › Supplemental_Code/ConsDB/docs/pages.html]

ConsDB: Related Pages


|  |
| --- |
| ConsDB  1.0  Tool for creating consensus genomes from variant databases. |


Related Pages

Here is a list of all related documentation pages:

|  |  |
| --- | --- |
| ConsDB |  |


---

Generated by  

 1.8.17
