# Supplementary material for: Pan-human consensus genome significantly improves the accuracy of RNA-seq analyses
Source: Genome Res. 2022 Apr;32(4):738–49. doi: 10.1101/gr.275613.121 (PMC8997357; doi:10.1101/gr.275613.121)
Supplement: Supplemental Material [file supp_gr.275613.121_Supplemental_Information.pdf]

Supplementary Information

Consensus allele changes in isoform expression

We used RSEM (Li and Dewey 2011) to quantify transcript expression (TPM) for reads mapped to the reference and to the pan-human consensus, again using the haploid personal genome as the ground truth. In this analysis, we excluded insertions and deletions in order to avoid any discrepancies between the internal transformed genome generated by STAR and the genome used by RSEM to generate its genomic indices. The quantification error is defined as the $\log_2$-ratio of the TPM in the reference or pan-human consensus to the TPM in the personal (ground truth) genome. Although the vast majority of transcripts show very similar expression results for both genomes, there are many transcripts with large quantification errors (Supplementary Figure Supplementary Figures S34A-C). To reduce noise, we filtered the transcripts with low expression in all three genomes at three TPM thresholds of 0.2, 1 and 5 (Supplementary Figure Supplementary Figures S34B-C). For all three thresholds, there were ~6 times as many transcripts for which the quantification error in the reference was higher than that in the pan-human consensus (Supplementary Figure Supplementary Figure S34C).

To illustrate the effect of consensus genomes on the transcript quantification, we looked at a transcript of the *ALDH3A2* gene, which has zero expression in the personal genome and the pan-human consensus genome (no error), but at the same time exhibits non-zero expression in the reference (~2 TPM), which signifies a large error with respect to the ground truth. A genome browser snapshot of selected regions of this gene is shown in Supplementary Figure Supplementary Figure S34D, highlighting the effects that MARs can have on read mapping and on transcript expression prediction. In this case, the reference contains the minor allele, which causes reads to map to the short exon, and hence the isoform (ENST00000582991.5) containing this exon has non-zero expression. At the same time, both the pan-human consensus and personal HG00512 genome contain the major allele, which prompts the read alignments to skip the short exon, resulting in zero expression for the ENST00000582991.5 isoform.

Isoform expression calculations

The genome generation and mapping procedures for the transcript expression calculations were similar to the procedures for the mapping error section, however there were some key differences. The main difference in the genome generation step was the exclusion of insertions and deletions from the pan-human consensus and HG00512 personal haploid genome. We excluded insertions and deletions for this analysis in order to avoid any discrepancies between the internal transformed genome generated by STAR and the genome used by RSEM to generate its genomic indices. The VCF files used to generate the pan-human consensus and the HG00512 personal haploid genome were generated by using an awk script to remove insertions and deletions from the VCF files that were used to generate the genomes for the mapping error section. We generated the STAR genomes as previously described, using the SNP-only VCF files. We generated the RSEM genome indices using transformed genome FASTA files, which were made using BCFtools to incorporate the SNPs in the VCF files into the PAR-masked genome.

The mapping steps for this analysis were the same as previously described, with the addition of the `--quantMode GeneCounts TranscriptomeSAM` option to force STAR to export transcriptomic alignments in addition to the standard genomic alignments. We ran RSEM using these STAR-generated transcriptomic alignments as the input.

We used Python scripts to calculate the transcript expression error for each genome, using the TPM values from the RSEM predictions. The $\log_2$ fold change values shown in Supplementary Figure Supplementary Figure S34A were plotted without normalization, and the transcripts for which either the pan-human consensus or the reference had an estimated TPM of 0 were represented as arrows. Transcripts for which the personal genome had an estimated TPM of 0 were excluded because these transcripts would, by definition, have an infinite $\log_2$ fold change for both the pan-human consensus and the reference. Additionally, transcripts for which both the pan-human consensus and the

reference had an estimated TPM of 0 were excluded in this plot. In Supplementary Figure S34B-C, all TPM values were normalized by an addition of 0.001 in order to prevent infinite $\log_2$ fold change values.

Selection of transcript of interest

We selected transcript *ALDH3A2*-222 through a manual inspection process. We searched for a transcript with significant differential expression between the reference and the pan-human consensus, in order to find an example that would highlight the differences in transcript expression calculation between the two genomes. We used DESeq2 to determine statistical significance. However, DESeq2 requires replicates in its inputs, and the HGSV reads only contained one sample. In order to utilize DESeq2 with this data, we randomly split the transcriptomic alignment of each genome into two separate BAM files, ensuring that all alignments for a given read were grouped in the same file. This splitting was performed using a Python script. We then ran RSEM on these split BAM files, and used the expected_count column of the RSEM output as the input for DESeq2. Because this column can contain non-integer values, all expected_count values were rounded down to the previous integer. For our selection, we only considered transcripts with a DESeq2 adjusted p-value < 0.05. In addition, we also required that the gene to which the transcript belongs was protein coding.

Consensus alleles generate large changes in splice junction expression

Here, we explore the effect of replacing the reference with a consensus genome on splice junction expression. We define splice junction expression as the number of uniquely mapping reads which are spliced through the junction. Here we only consider annotated junctions, and define quantification error as the $\log_2$-ratio of the junction read counts in the reference or pan-human consensus to the junction read count in the personal genome (ground truth). Although the vast majority of splice junctions show very similar expression results for both genomes, there are many splice junctions with large quantification errors (Supplementary Figures S35A-C). To reduce noise, we filtered the splice junctions with low expression in all three genomes at three counts thresholds of 1, 10, and 25 (Supplementary Figures S35B-C). For all three thresholds, there were ~4-5 times as many splice junctions for which the quantification error in the reference was higher than that in the pan-human consensus (Supplementary Figure S35C).

To illustrate the effect of consensus genomes on splice junction expression, we looked at a splice junction in the *CBWD1* gene. This splice junction has very low expression in the reference, but is highly expressed in the pan-human consensus genome and the HG00512 personal genome. This disparity signifies a large error in the reference with respect to the ground truth, which is mitigated by the pan-human consensus. A genome browser snapshot of the region of the *CBWD1* gene containing this splice junction is shown in Supplementary Figure S35D, highlighting the effects that MARs can have on read mapping and on splice junction quantification. In this case, the reference contains the minor allele, which prevents reads from mapping to the exon. However, both the pan-human consensus and personal HG00512 genome contain the major allele, allowing the reads to be mapped to the exon. Because of this MAR, the isoforms containing this splice junction have erroneously low expression when reads are mapped to the reference. The pan-human consensus rectifies the problem, predicting high expression of these isoforms that agrees with the ground truth of the personal genome mapping.

Splice junction expression calculations

The splice junction expression values used in our calculations were generated during the previously described read mapping section. Specifically, we used the number of uniquely mapped reads crossing the splice junction, which is given in the 7th column of the SJ.out.tab file generated by STAR. To calculate the quantification error for each genome, we used custom Python scripts. The $\log_2$ fold change values shown in Supplementary Figure S35A were plotted without normalization, and the splice junctions for which the reference had no unique reads crossing the splice junction were represented as arrows. Splice junctions for which both the pan-human consensus and the reference had 0 expression were excluded from this plot. Additionally, these $\log_2$ fold change values were thresholded to +/- 5. Splice junctions with an absolute log fold change > 1.5 and a max expression value > 50 were labeled with the gene in which they fall. In Supplementary Figures S35B-C, all read count values were normalized by an addition of 0.001 in order to prevent infinite $\log_2$ fold change values.

Selection of splice junction of interest

We selected the splice junction through a manual inspection process. We searched for a splice junction with a large absolute $\log_2$ fold change between the reference and the pan-human consensus, in order to find an example that would highlight the differences in splice junction expression between the two genomes. We also required that the splice junction fall within a protein coding gene.

Construction of a haploid personal genome from a diploid genome

To construct a haploid personal genome from a diploid genome, we incorporate all alleles that are homozygous for a variant, and randomly select an allele for heterozygous variants (Supplementary Figure S36). Since typically both heterozygous alleles are present in the RNA-seq, choosing one of them randomly should be neutral for alignment accuracy on average, neither improving the alignment or making it worse. Hence, a haploid genome is a good proxy for the true personal diploid genome as it maintains the alignment accuracy of homozygous variants while not affecting the heterozygous variants.
