# Supplementary material for: Pan-human consensus genome significantly improves the accuracy of RNA-seq analyses
Source: Genome Res. 2022 Apr;32(4):738–49. doi: 10.1101/gr.275613.121 (PMC8997357; doi:10.1101/gr.275613.121)
Supplement: Supplemental Material [file supp_gr.275613.121_Supplemental_Tables.pdf]

	Ref	Pan	AFR	AFR_YRI
	**All reads**			
Number of input reads	63,949,386	63,949,386	63,949,386	63,949,386
Uniquely mapped reads %	91.21%	91.17%	91.19%	91.19%
Average mapped length	101.7	101.7	101.7	101.7
Mismatch rate per base, %	0.19%	0.18%	0.18%	0.18%
Deletion rate per base	0.00%	0.00%	0.00%	0.00%
Insertion rate per base	0.00%	0.00%	0.00%	0.00%
% of reads mapped to multiple loci	6.72%	6.76%	6.75%	6.74%
	**Reads that overlap personal homozygous SNPs**			
Number of input reads	1,384,158	1,384,158	1,384,158	1,384,158
Uniquely mapped reads %	92.55%	93.70%	94.45%	94.49%
Average mapped length	101.6	101.7	101.7	101.7
Mismatch rate per base, %	1.30%	0.58%	0.50%	0.49%
Deletion rate per base	0.01%	0.01%	0.01%	0.01%
Insertion rate per base	0.01%	0.00%	0.00%	0.00%
% of reads mapped to multiple loci	7.45%	6.23%	5.48%	5.44%
	**Read that overlap personal homozygous indels**			
Number of input reads	93,119	93,119	93,119	93,119
Uniquely mapped reads %	96.12%	98.04%	98.04%	98.04%
Average mapped length	99.1	100.8	100.9	100.9
Mismatch rate per base, %	0.52%	0.33%	0.32%	0.31%
Deletion rate per base	0.45%	0.20%	0.16%	0.16%
Insertion rate per base	0.38%	0.12%	0.12%	0.12%
% of reads mapped to multiple loci	3.88%	1.83%	1.83%	1.82%

Supplementary Table S1: Summary statistics for alignment to the reference and consensus genomes for individual NA12938.

Gene Name	log2(Pan/Ref)	CPM Ref	CPM Pan	p_adj	Gene Type	Gene Description	Phenotype Description
RRN3P2	-1.09	28.70	13.71	2.6E-29	lncRNA	RRN3 pseudogene 2	
ARMC9	-1.53	13.14	4.57	3.2E-23	protein_coding	armadillo repeat containing 9	Joubert Syndrome
BEND7	-3.56	4.73	0.42	8.1E-23	protein_coding	BEN domain containing 7	
THSD4-AS1	-2.83	4.86	0.70	1.2E-18	lncRNA	THSD4 antisense RNA 1	
EHD2	2.28	1.15	5.98	2.9E-18	protein_coding	EH domain containing 2	
ADGRL3-AS1	1.08	6.23	12.82	2.6E-15	lncRNA	ADGRL3 antisense RNA 1	
PCOLCE2	-3.80	2.17	0.16	3.5E-11	protein_coding	procollagen C-endopeptidase enhancer 2	
AL590683.1	-2.07	3.48	0.86	9.8E-11	lncRNA	long intergenic non-protein coding RNA 2800	
KAZN-AS1	3.12	0.29	2.49	4.2E-10	lncRNA	KAZN antisense RNA 1	
C3orf67	-1.50	5.50	1.98	2.6E-09	protein_coding	CFAP20 domain containing	
EVPL	1.87	0.93	3.74	3.7E-09	protein_coding	envoplakin	
CCDC40	1.36	1.98	5.18	3.4E-08	protein_coding	coiled-coil domain containing 40	Primary ciliary dyskinesia
KIZ-AS1	-0.80	11.95	6.71	6.8E-08	lncRNA	KIZ antisense RNA 1	
LRP1B	-1.06	6.17	2.97	6.1E-07	protein_coding	LDL receptor related protein 1B	
RIMS1	-2.39	1.82	0.38	1.1E-06	protein_coding	regulating synaptic membrane exocytosis 1	Cone rod dystrophy
ALDH7A1	-1.40	4.00	1.66	2.1E-06	protein_coding	aldehyde dehydrogenase 7 family member A1	Pyridoxine-dependent epilepsy
AMBN	-2.35	1.85	0.35	2.1E-06	protein_coding	ameloblastin	Hypoplastic amelogenesis imperfecta
SPARCL1	1.76	0.74	2.46	3.4E-06	protein_coding	SPARC like 1	
AC006480.2	-1.12	4.60	2.05	6.4E-06	lncRNA	novel transcript	
C6orf10	-2.20	1.76	0.42	7.3E-06	protein_coding	testis expressed basic protein 1	
AL033504.1	-2.13	1.73	0.42	7.4E-06	lncRNA	novel transcript	
SLC6A7	-3.19	1.21	0.13	2.0E-05	protein_coding	solute carrier family 6 member 7	
KCNB2	-1.72	2.05	0.61	5.0E-05	protein_coding	potassium voltage-gated channel subfamily B member 2	
AL049812.3	-2.91	1.02	0.13	7.4E-05	lncRNA	novel transcript	
LINC02269	-2.59	1.18	0.22	7.4E-05	lncRNA	long intergenic non-protein coding RNA 2269	
SEZ6L	-1.57	2.14	0.67	8.0E-05	protein_coding	seizure related 6 homolog like	
AC095050.1	-22.31	0.67	0.00	1.0E-04	lncRNA	novel transcript	
AC108215.1	3.61	0.06	0.93	1.2E-04	TEC	TEC	
CDH18-AS1	4.50	0.03	0.74	1.2E-04	lncRNA	CDH18 antisense RNA 1	
ASPH	-0.40	27.59	21.67	1.2E-04	protein_coding	aspartate beta-hydroxylase	Facial dysmorphism-lens dislocation-ant
LINC00908	-1.35	2.40	0.93	1.7E-04	lncRNA		
AC135050.4	-1.44	2.27	0.83	2.2E-04	lncRNA		
AC025265.2	-1.59	1.89	0.61	2.6E-04	lncRNA		
LINC01500	-1.19	3.20	1.53	4.1E-04	lncRNA	long intergenic non-protein coding RNA 1500	
LINC01592	1.81	0.42	1.47	6.8E-04	lncRNA	long intergenic non-protein coding RNA 1592	
TF	0.65	4.79	7.10	7.1E-04	protein_coding	transferrin	Congenital atransferrinemia
AC048337.1	2.20	0.26	1.09	1.2E-03	lncRNA	novel transcript	
FGF1	-0.68	5.66	3.48	2.0E-03	protein_coding	fibroblast growth factor 1	
PPP1R3A	2.21	0.19	0.86	2.4E-03	protein_coding	protein phosphatase 1 regulatory subunit 3A	TYPE 2 DIABETES MELLITUS
CREB3L3	-1.68	1.21	0.38	2.8E-03	protein_coding	cAMP responsive element binding protein 3 like 3	
AC013391.2	0.89	1.89	3.45	2.8E-03	lncRNA	novel transcript	
AC093895.1	1.92	0.26	0.99	2.9E-03	lncRNA	novel transcript	
AC073320.1	-0.59	7.42	5.11	4.0E-03	lncRNA		
HGF	-2.21	0.86	0.22	4.1E-03	protein_coding	hepatocyte growth factor	Autosomal recessive non-syndromic ser
MCM8	-0.49	10.96	8.05	4.2E-03	protein_coding	minichromosome maintenance 8 homologous recom	NON RARE IN EUROPE: Primary ovaria
AC010319.4	-0.85	3.39	1.85	4.8E-03	lncRNA		
AL049775.2	2.58	0.13	0.64	6.6E-03	lncRNA	novel transcript	
SLC17A1	2.48	0.16	0.77	6.6E-03	protein_coding	solute carrier family 17 member 1	
BICC1	-1.16	1.79	0.83	6.7E-03	protein_coding	BicC family RNA binding protein 1	Autosomal dominant polycystic kidney d
PLCXD3	1.43	0.45	1.21	7.9E-03	protein_coding	phosphatidylinositol specific phospholipase C X domain containing 3	
AC068512.1	-3.00	0.51	0.06	8.1E-03	lncRNA	MARCHF10 divergent transcript	
AL121935.1	0.82	1.92	3.32	1.3E-02	lncRNA	novel transcript	
PQLC2L	3.82	0.03	0.38	1.3E-02	transcribed_unita	solute carrier family 66 member 1 like	
AL035467.2	-1.00	1.92	0.96	1.5E-02	lncRNA	novel transcript	
FRMPD2	2.11	0.13	0.64	1.7E-02	protein_coding	FERM and PDZ domain containing 2	
RYR2	-0.65	3.74	2.40	2.0E-02	protein_coding	ryanodine receptor 2	Catecholaminergic polymorphic ventricu
AC078785.1	-0.42	15.09	11.92	2.4E-02	lncRNA	novel transcript	
LHFPL3	-0.58	5.05	3.45	2.4E-02	protein_coding	LHFPL tetraspan subfamily member 3	
SLC15A1	-1.79	0.67	0.19	3.8E-02	protein_coding	solute carrier family 15 member 1	
LINC00265	-0.43	7.51	5.56	5.0E-02	lncRNA	long intergenic non-protein coding RNA 265	
TMEM132C	-0.76	2.30	1.31	5.0E-02	protein_coding	transmembrane protein 132C	
ABCB5	-0.92	1.63	0.86	5.3E-02	protein_coding	ATP binding cassette subfamily B member 5	
TMC2	-0.79	2.08	1.18	5.8E-02	protein_coding	transmembrane channel like 2	
LINC02004	-2.44	0.38	0.06	6.1E-02	lncRNA	long intergenic non-protein coding RNA 2004	
PACRG	-0.49	7.51	5.72	6.6E-02	protein_coding	parkin coregulated	
KLKB1	-0.65	3.00	1.92	7.2E-02	protein_coding	kallikrein B1	Congenital prekallikrein deficiency
TEKT4	-1.94	0.48	0.13	7.3E-02	protein_coding	tektin 4	
GRIN2A	-1.20	0.90	0.42	7.5E-02	protein_coding	glutamate ionotropic receptor NMDA type subunit 2A	Continuous spikes and waves during sle
TEKT1	0.88	1.02	1.76	7.7E-02	protein_coding	tektin 1	
ZNF215	1.14	0.67	1.31	8.2E-02	protein_coding	zinc finger protein 215	
EFS	-1.42	0.70	0.26	8.2E-02	protein_coding	embryonal Fyn-associated substrate	
SLC22A2	-1.72	0.51	0.16	8.3E-02	protein_coding	solute carrier family 22 member 2	
LINC01484	0.73	1.25	2.05	8.6E-02	lncRNA	long intergenic non-protein coding RNA 1484	
ANO2	-0.77	1.76	0.99	9.6E-02	protein_coding	anoctamin 2	

Supplementary Table S2: Gene names, biotypes, descriptions, and phenotypes obtained from ENSEMBL BioMart for the genes significantly differentially expressed between the pan-human consensus and the reference genome in the T-cell cluster (Figure 4).
