# Supplementary material for: Pan-human consensus genome significantly improves the accuracy of RNA-seq analyses
Source: Genome Res. 2022 Apr;32(4):738–49. doi: 10.1101/gr.275613.121 (PMC8997357; doi:10.1101/gr.275613.121)
Supplement: Supplemental Material [file supp_gr.275613.121_Supplemental_Figures.pdf]

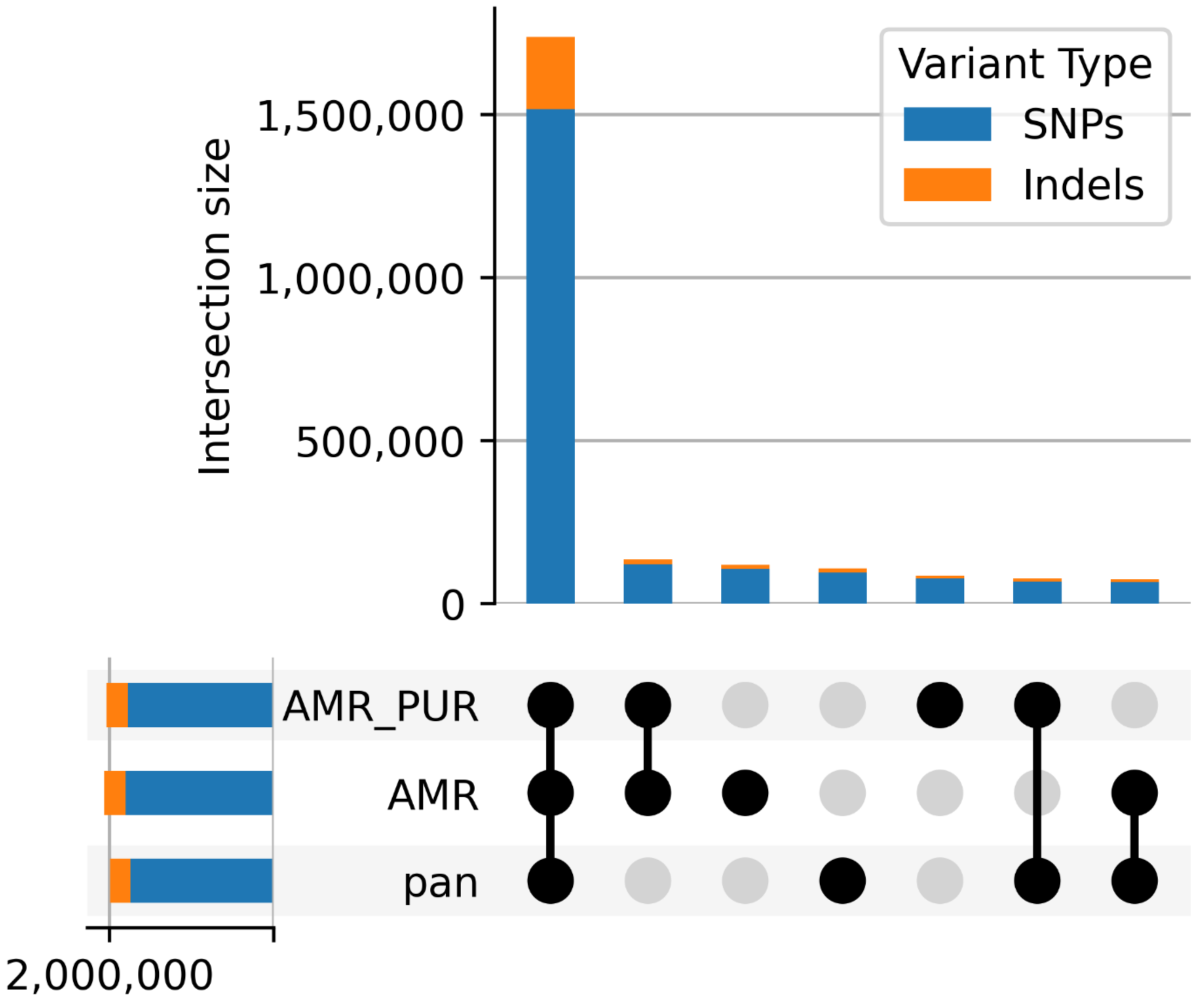

Supplementary Figure S1: Number of SNPs and indels shared between different combinations of the pan-human, super-population, and population consensus genomes for the Admixed American population.

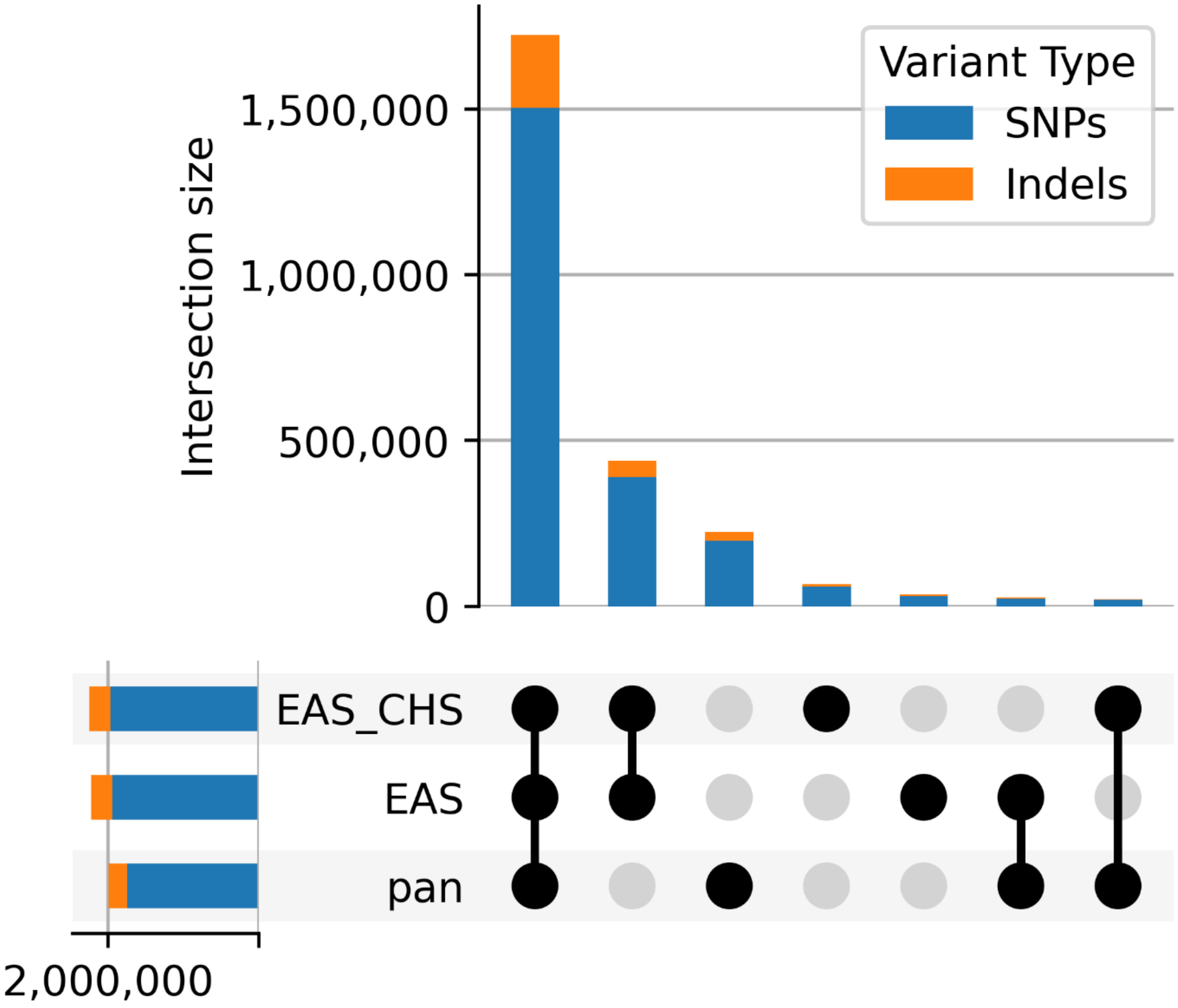

Supplementary Figure S2: Number of SNPs and indels shared between different combinations of the pan-human, super-population, and population consensus genomes for the East Asian population.

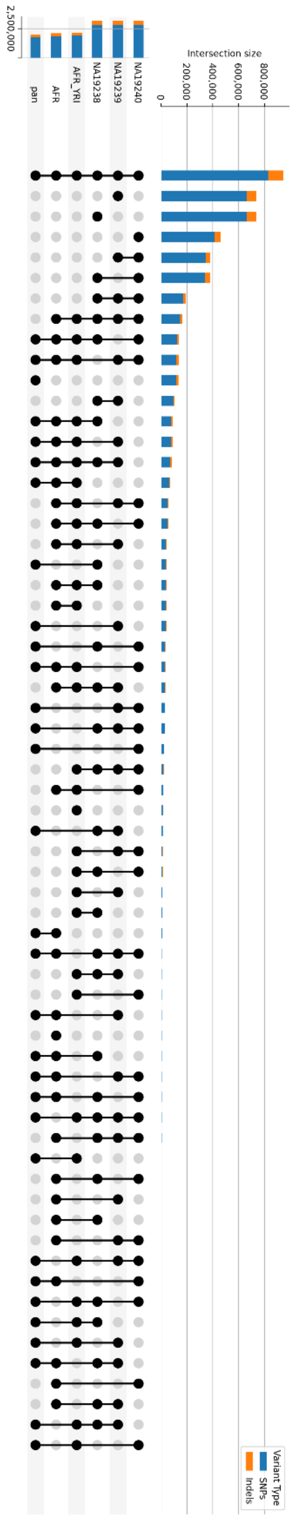

Supplementary Figure S3: Number of SNPs and indels shared between different combinations of the pan-human, super-population, and population consensus genomes, and individual haploid personal genomes for the African population.

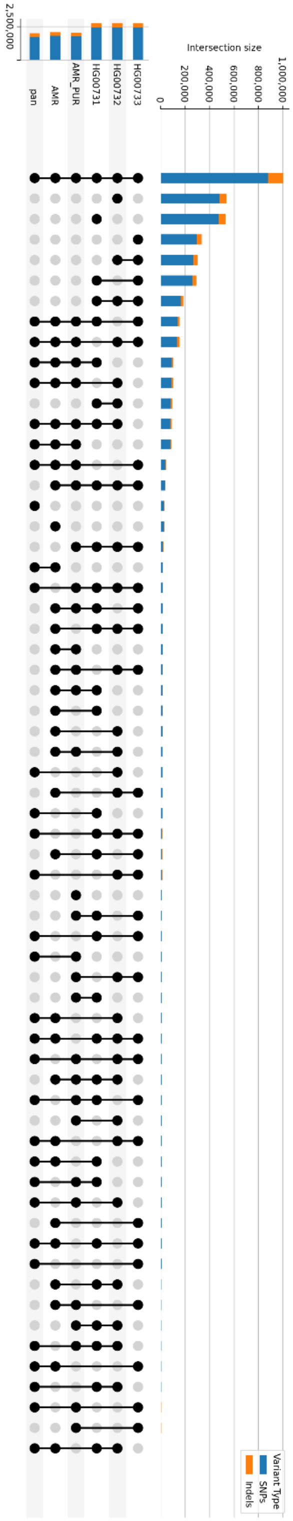

Supplementary Figure S4: Number of SNPs and indels shared between different combinations of the pan-human, super-population, and population consensus genomes, and individual haploid personal genomes for the Admixed American population.

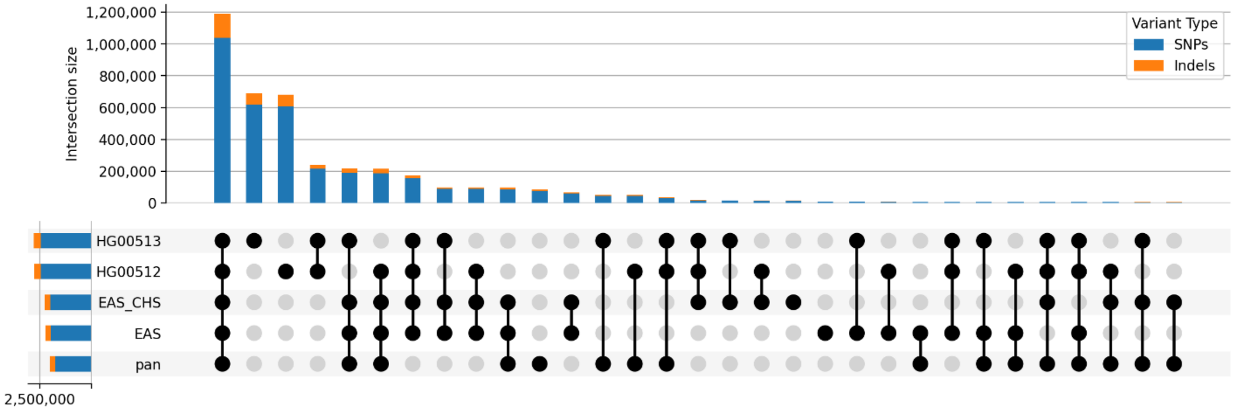

Supplementary Figure S5: Number of SNPs and indels shared between different combinations of the pan-human, super-population, and population consensus genomes, and individual haploid personal genomes for the East Asian population.

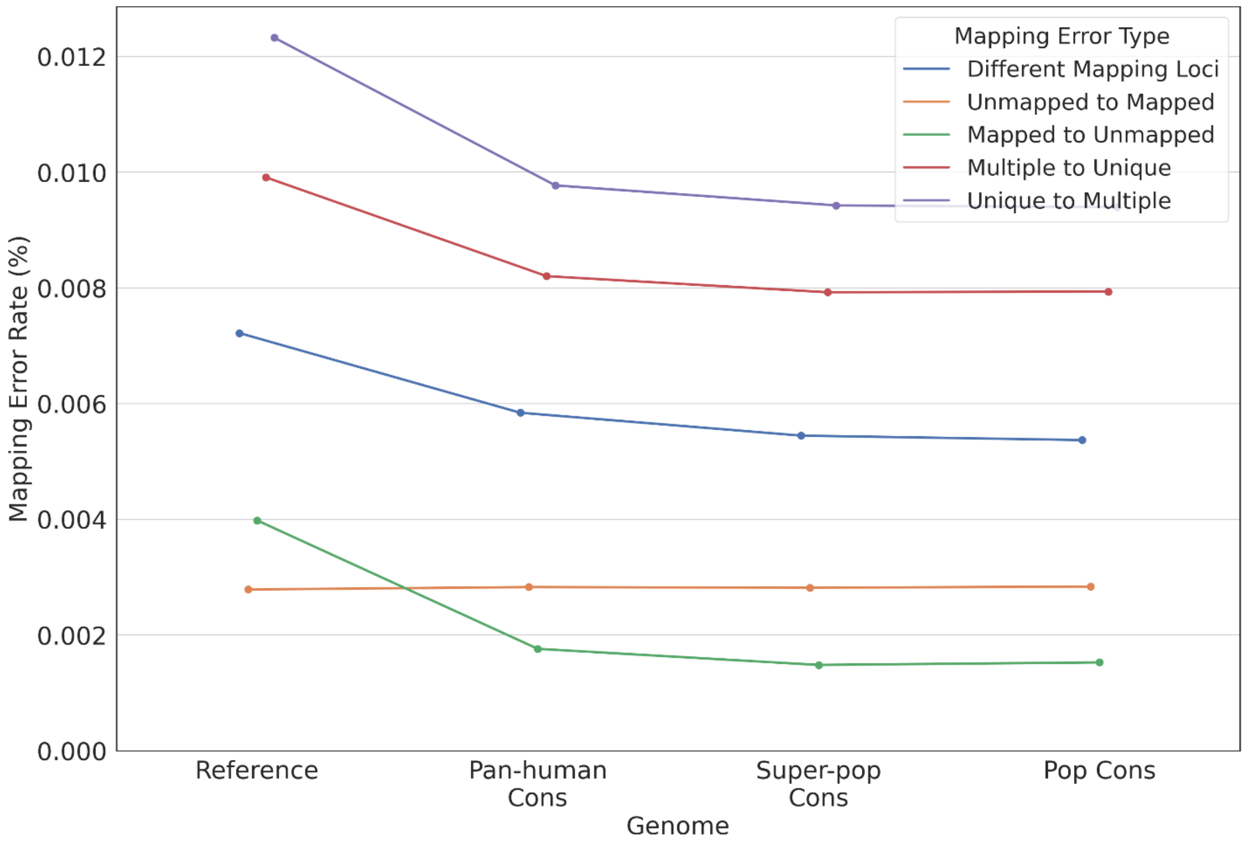

Supplementary Figure S6: Overall mapping error rate of reads overlapping insertions or deletions for each error type for individual NA19238.

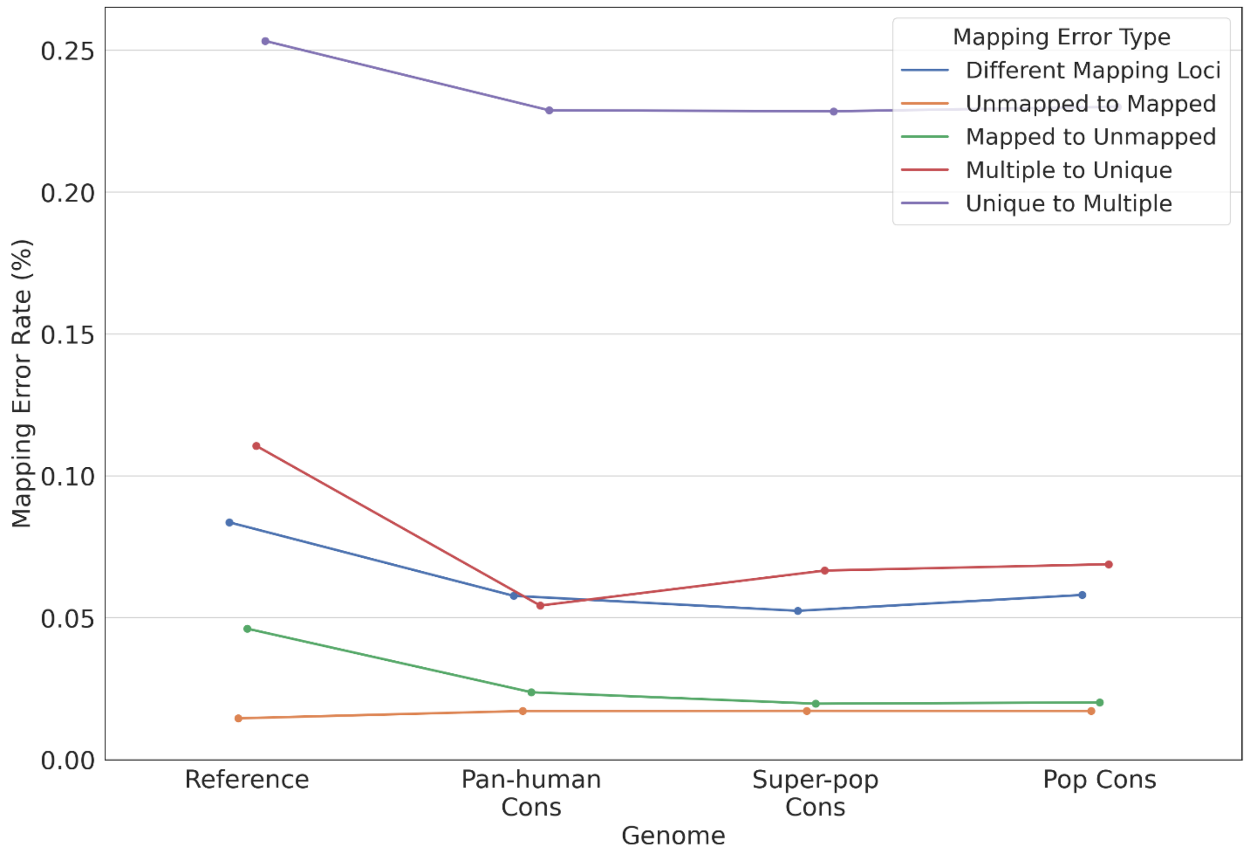

Supplementary Figure S7: Overall mapping error rate for each error type for individual HG00512.

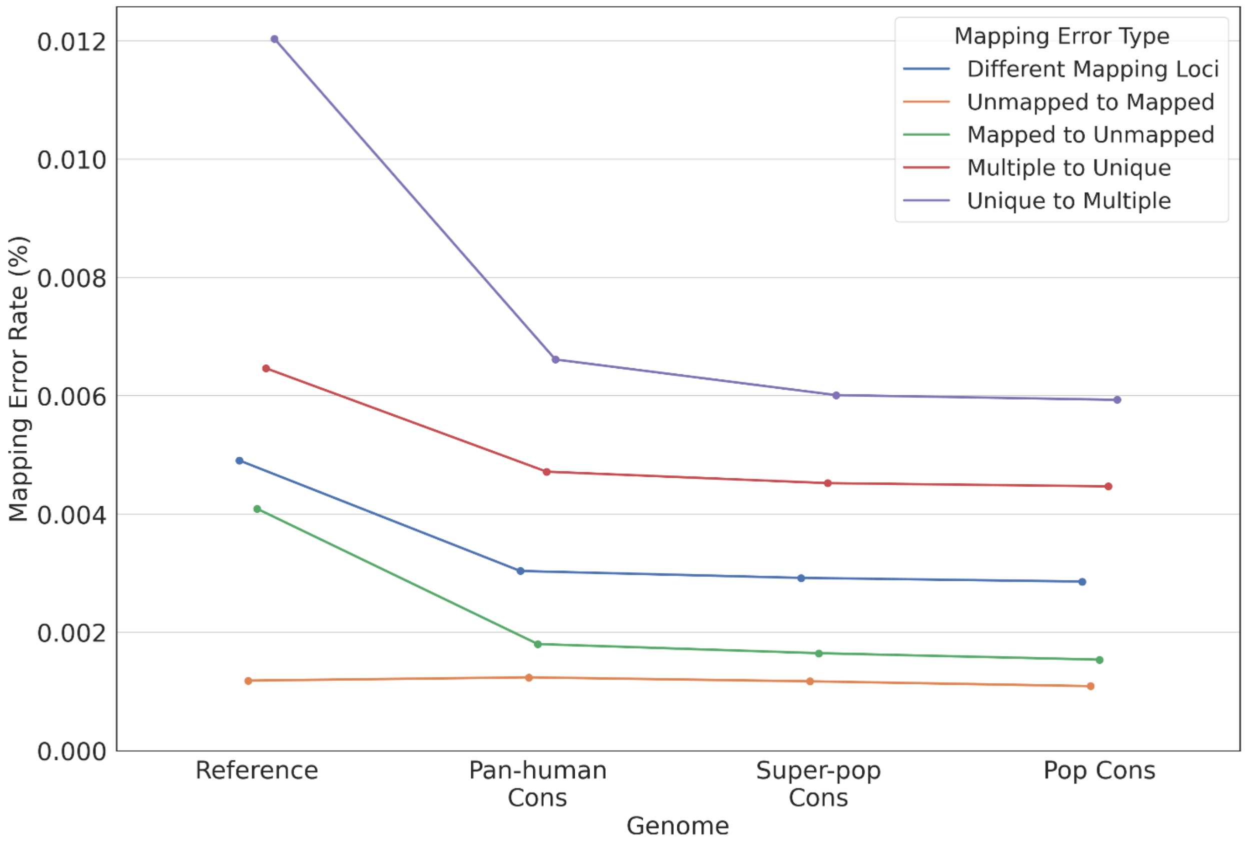

Supplementary Figure S8: Overall mapping error rate of reads overlapping insertions or deletions for each error type for individual HG00512.

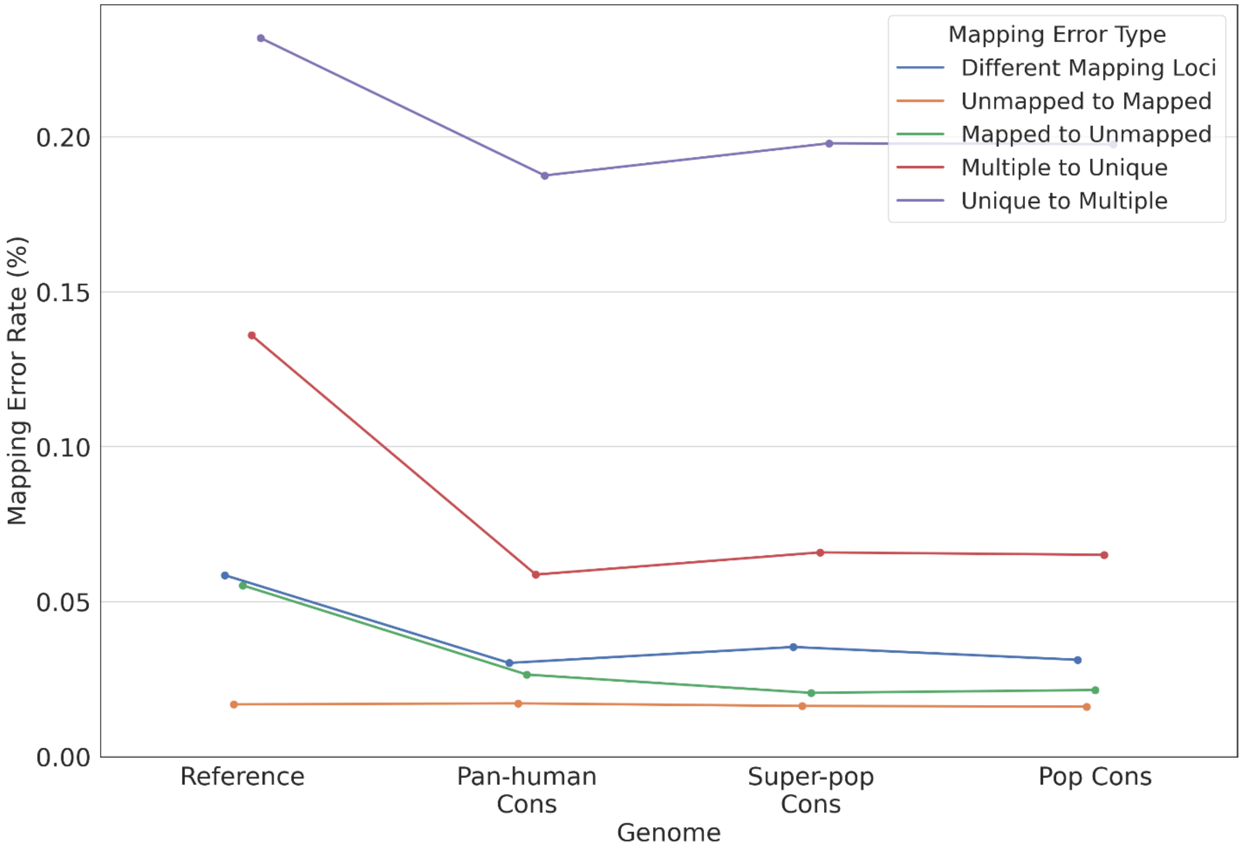

Supplementary Figure S9: Overall mapping error rate for each error type for individual HG00513.

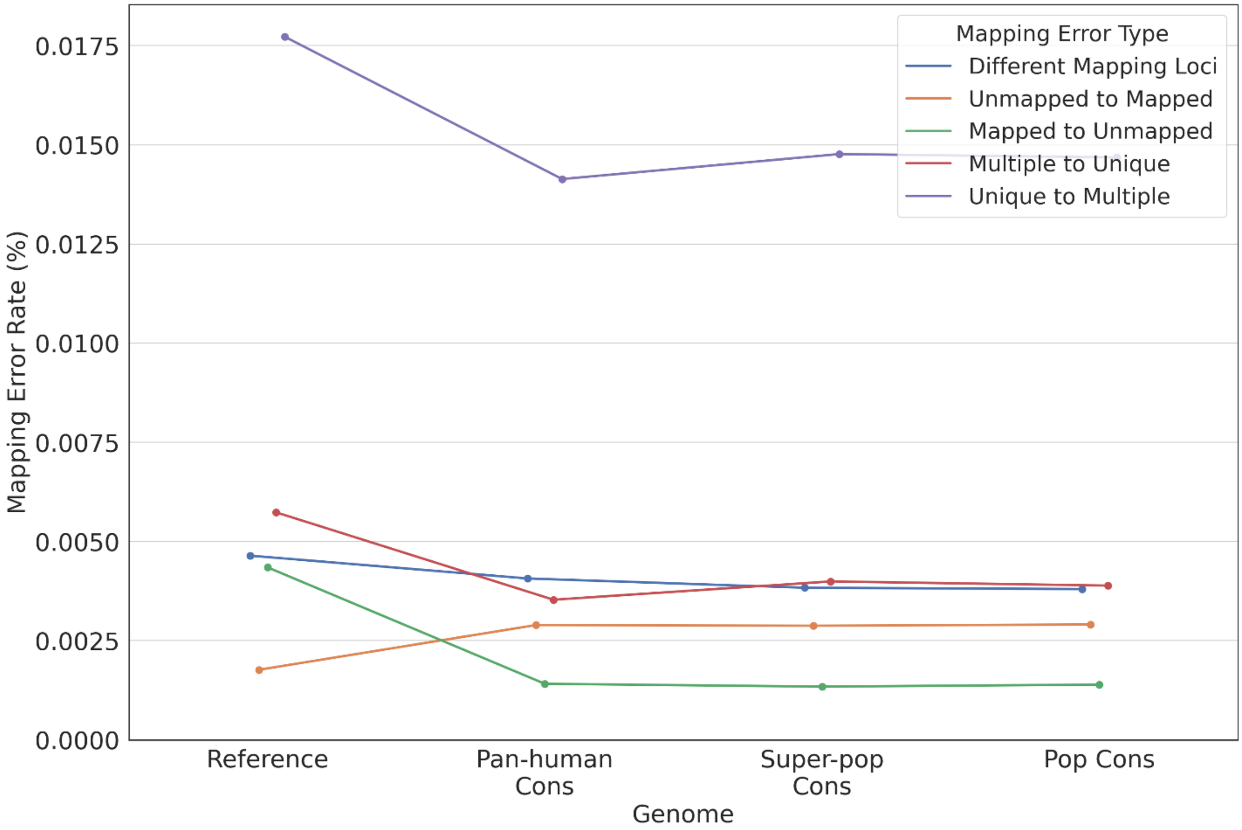

Supplementary Figure S10: Overall mapping error rate of reads overlapping insertions or deletions for each error type for individual HG00513.

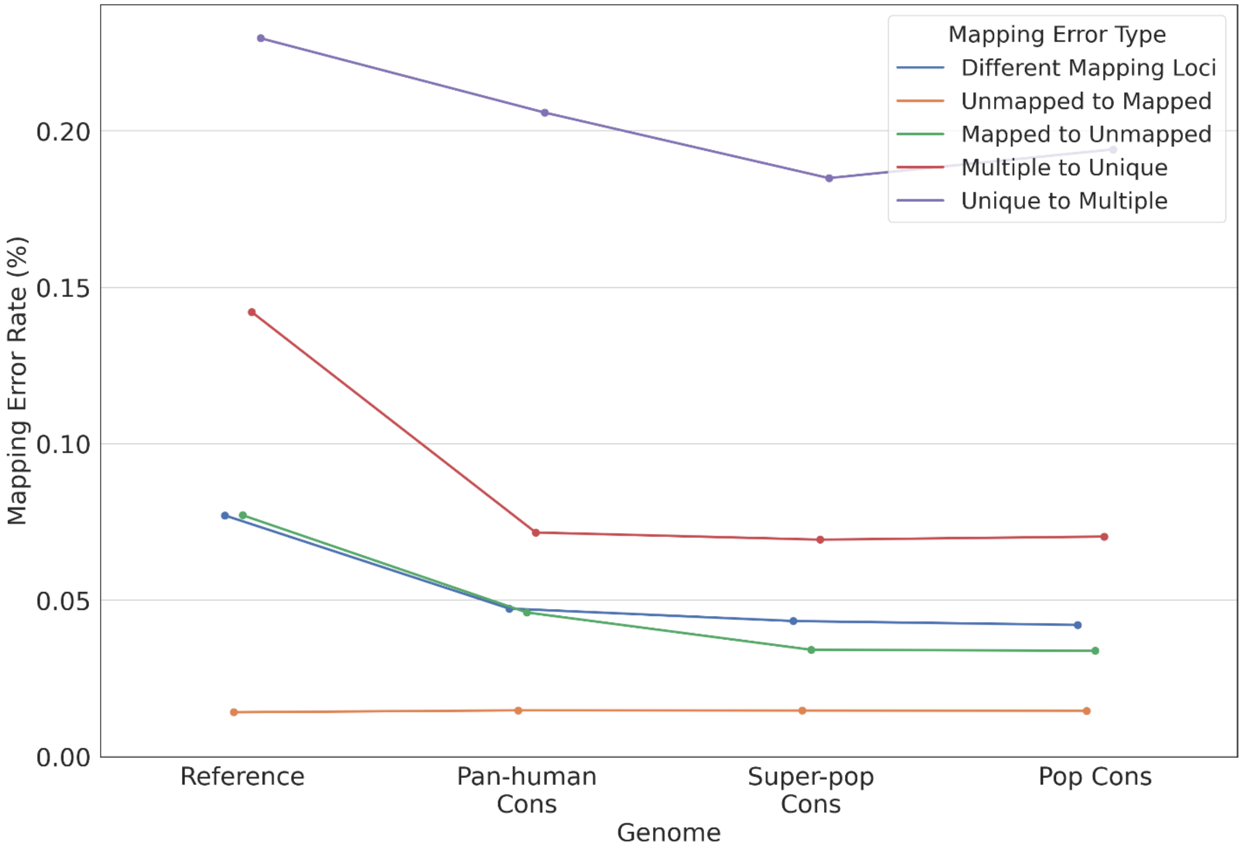

Supplementary Figure S11: Overall mapping error rate for each error type for individual HG00731.

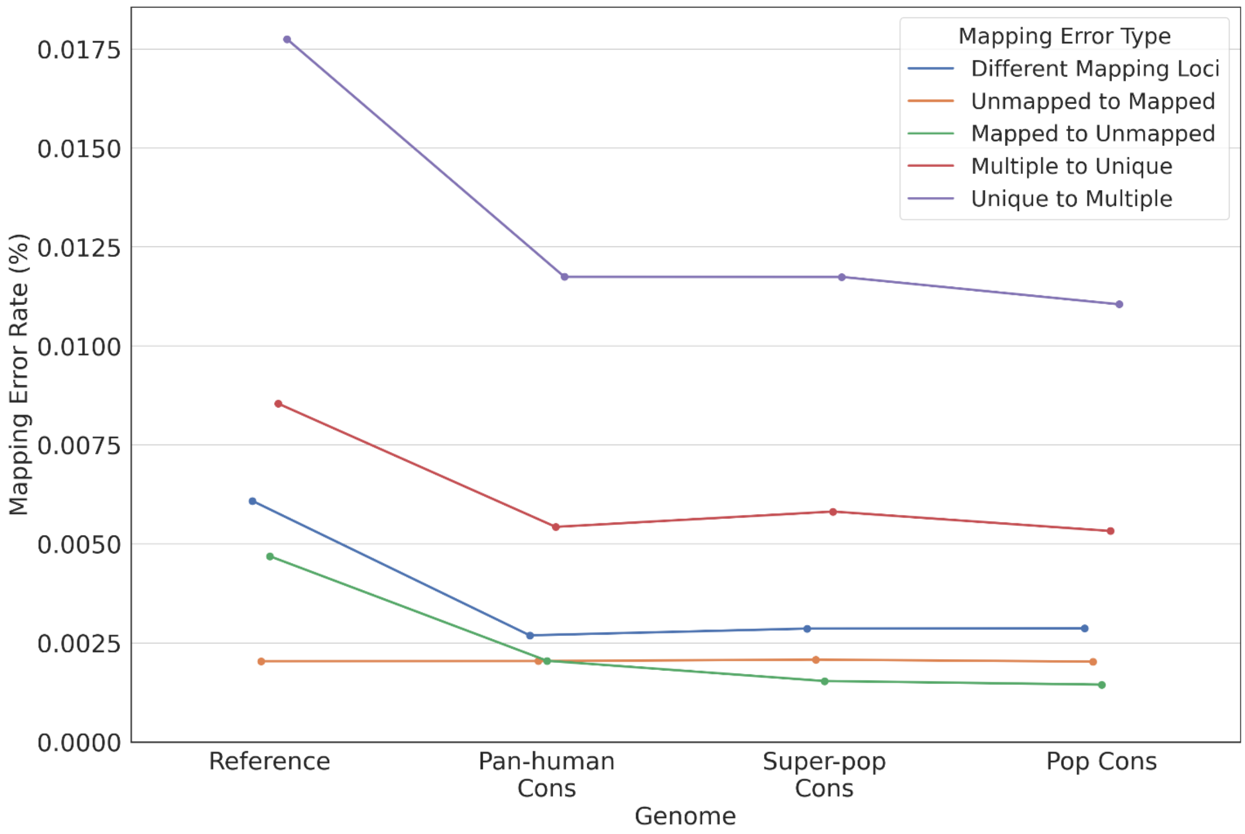

Supplementary Figure S12: Overall mapping error rate of reads overlapping insertions or deletions for each error type for individual HG00731.

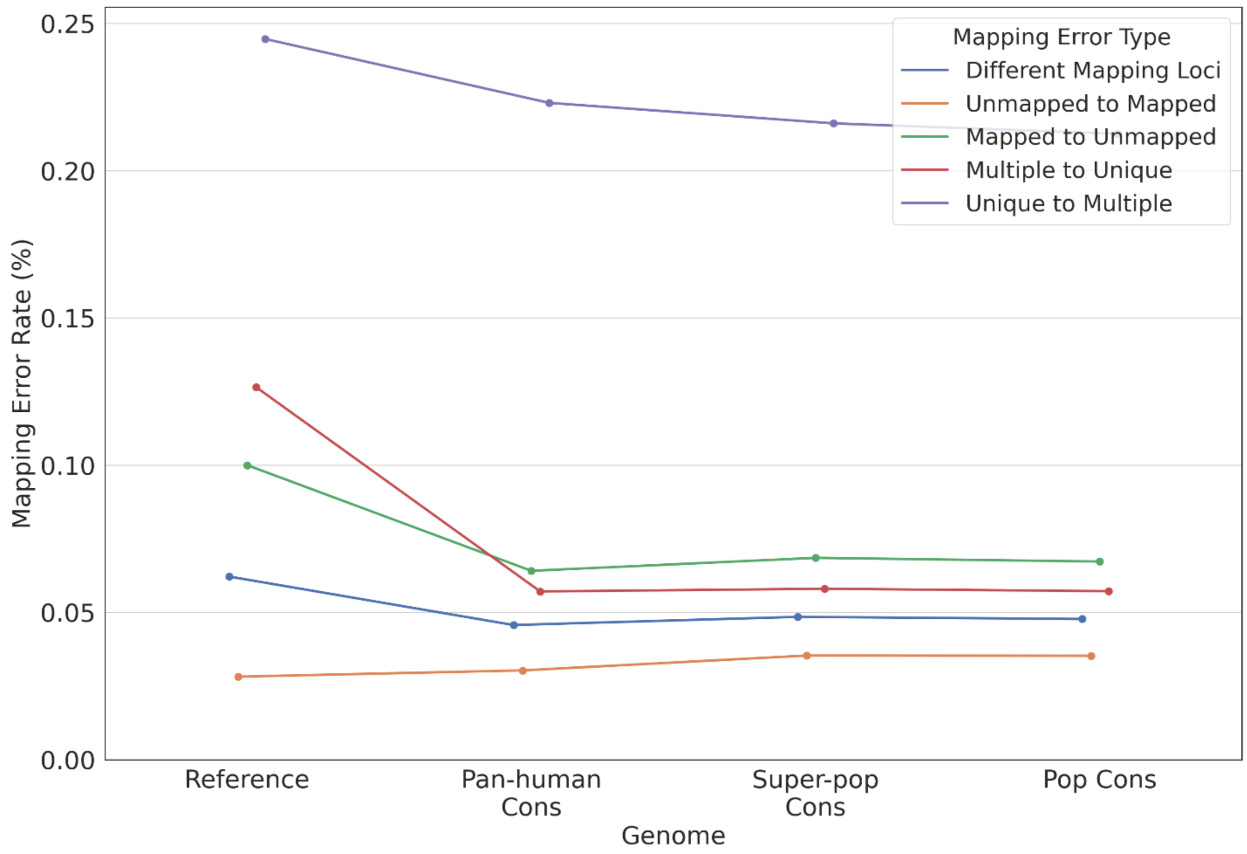

Supplementary Figure S13: Overall mapping error rate for each error type for individual HG00732.

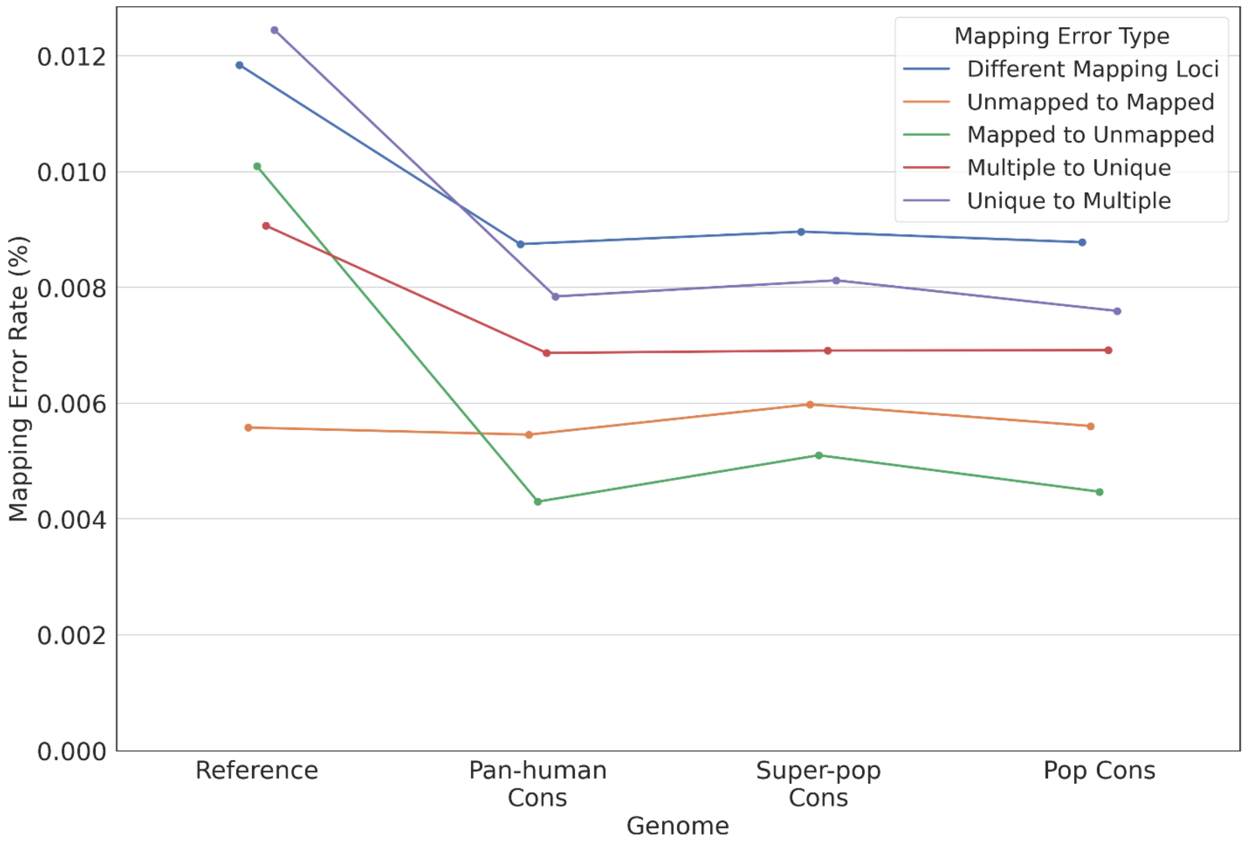

Supplementary Figure S14: Overall mapping error rate of reads overlapping insertions or deletions for each error type for individual HG00732.

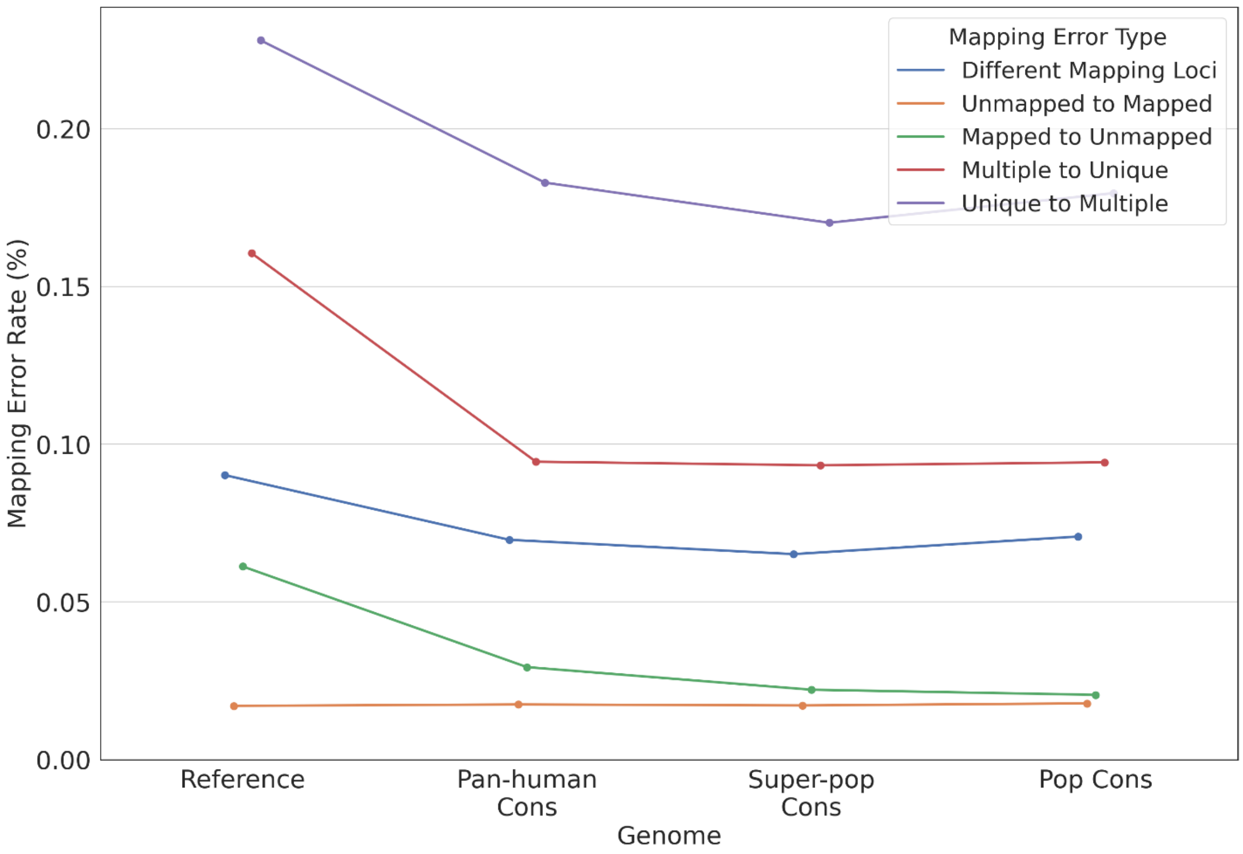

Supplementary Figure S15: Overall mapping error rate for each error type for individual HG00733.

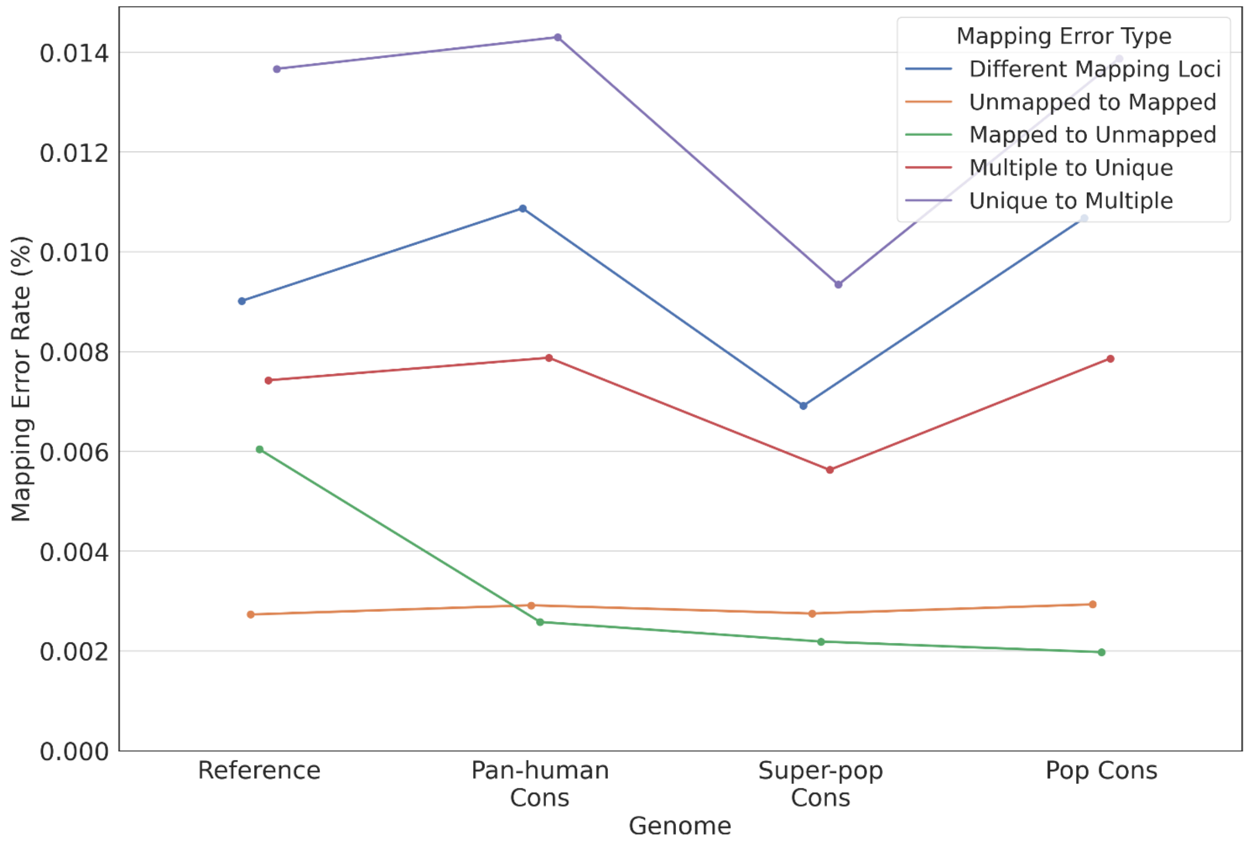

Supplementary Figure S16: Overall mapping error rate of reads overlapping insertions or deletions for each error type for individual HG00733.

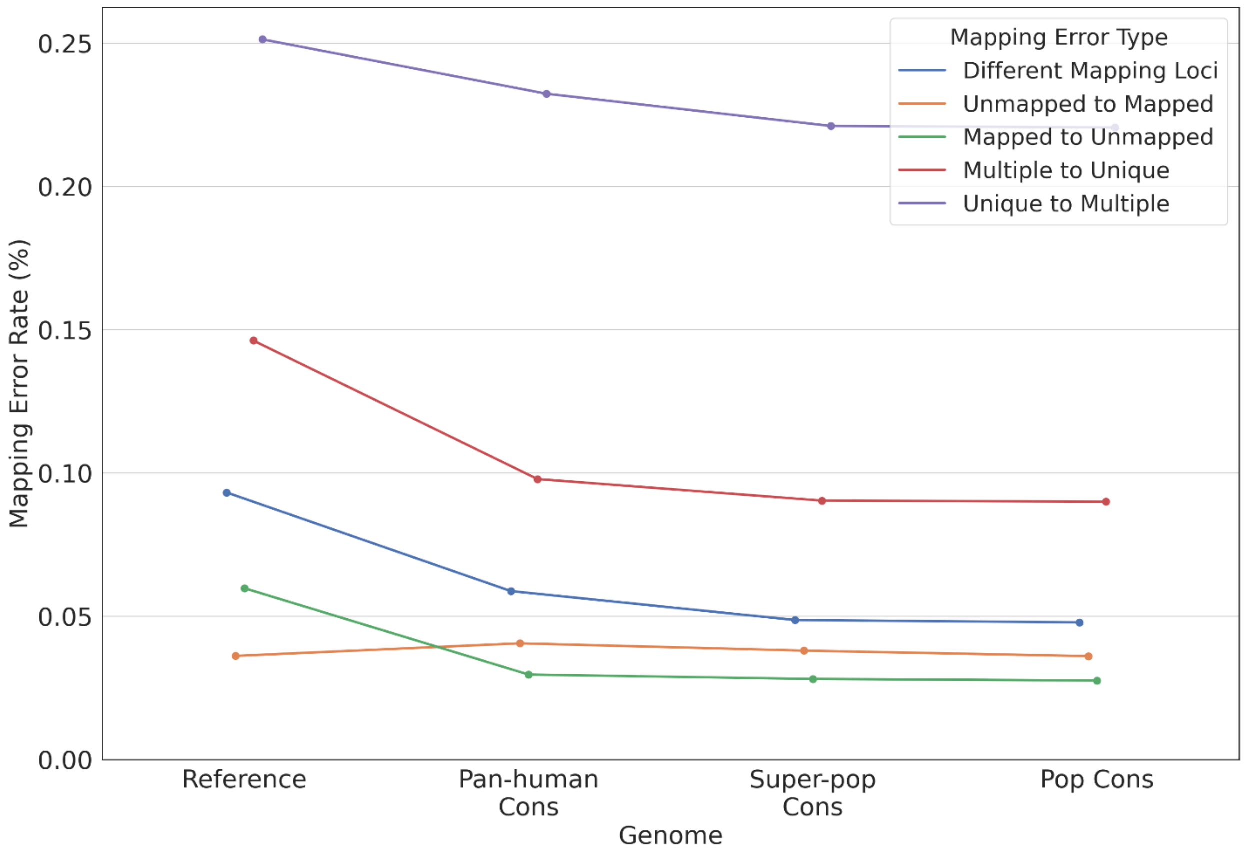

Supplementary Figure S17: Overall mapping error rate for each error type for individual NA19239.

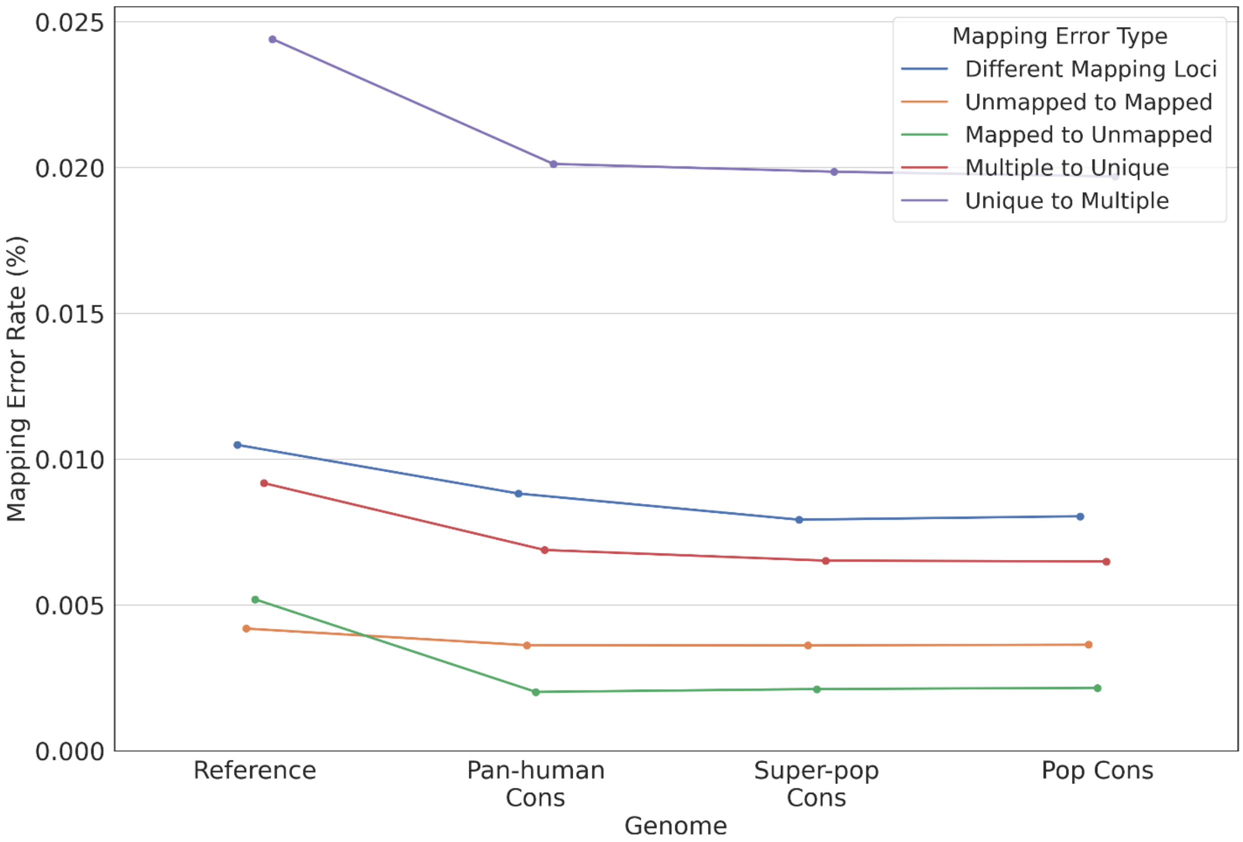

Supplementary Figure S18: Overall mapping error rate of reads overlapping insertions or deletions for each error type for individual NA19239.

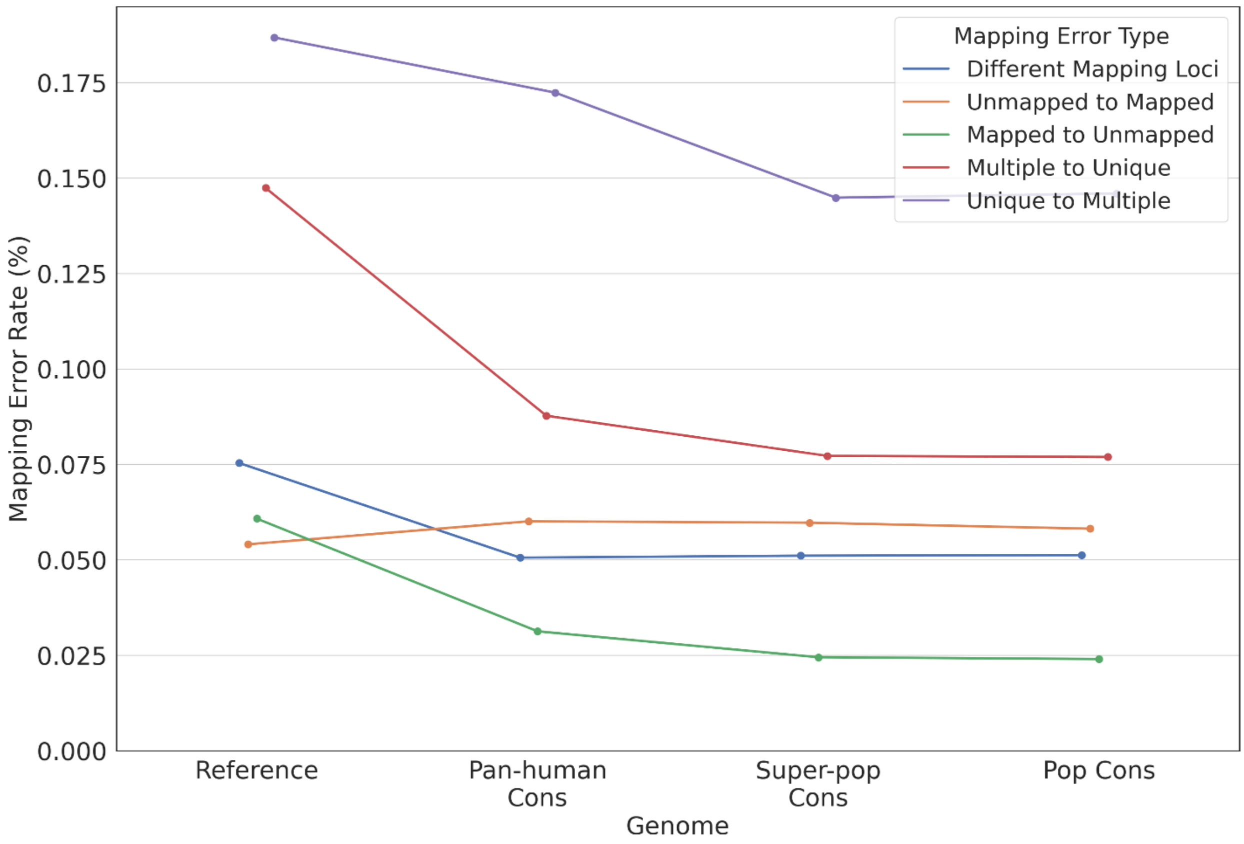

Supplementary Figure S19: Overall mapping error rate for each error type for individual NA19240.

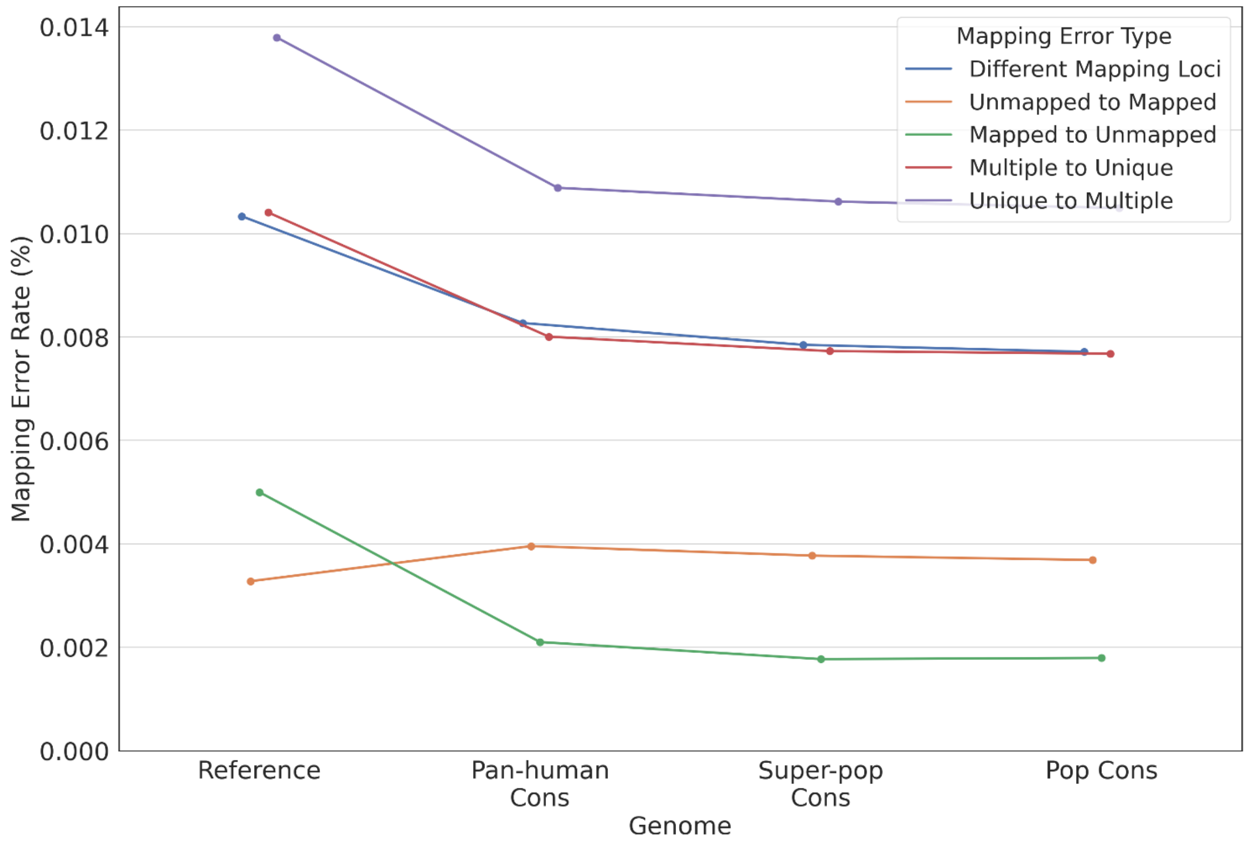

Supplementary Figure S20: Overall mapping error rate of reads overlapping insertions or deletions for each error type for individual NA19240.

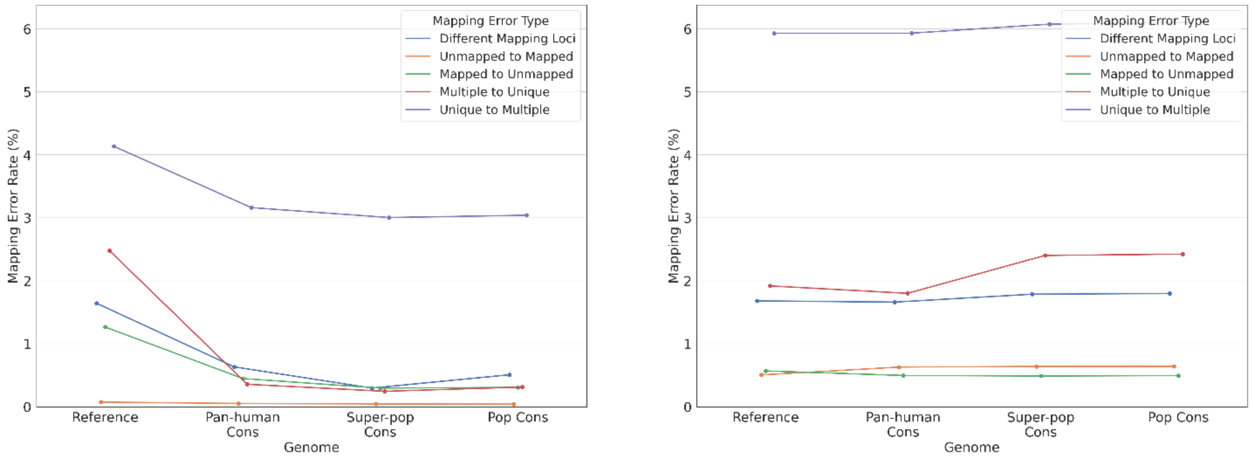

Supplementary Figure S21: Homozygous (left) and heterozygous (right) mapping error rate for each error type for individual HG00512.

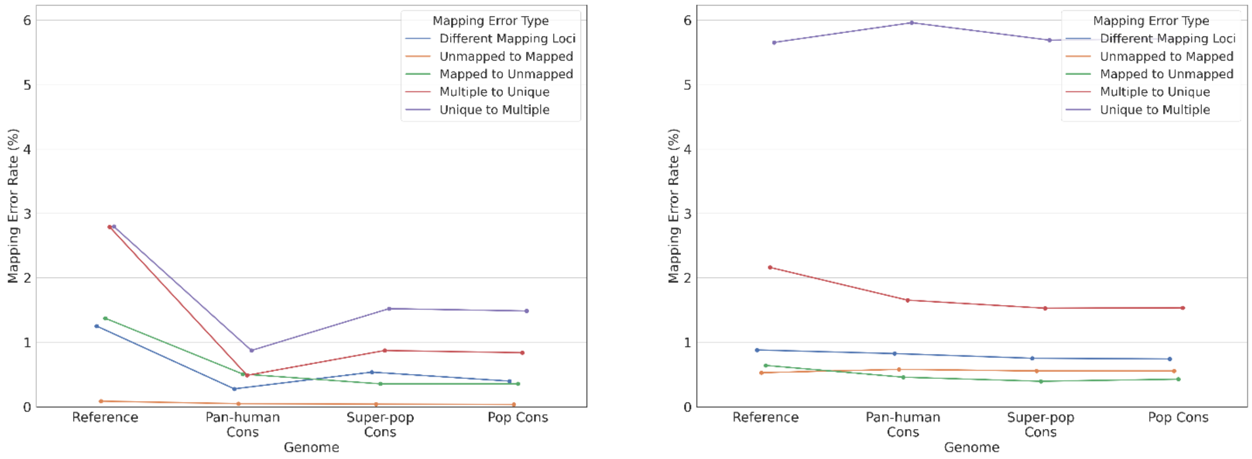

Supplementary Figure S22: Homozygous (left) and heterozygous (right) mapping error rate for each error type for individual HG00513.

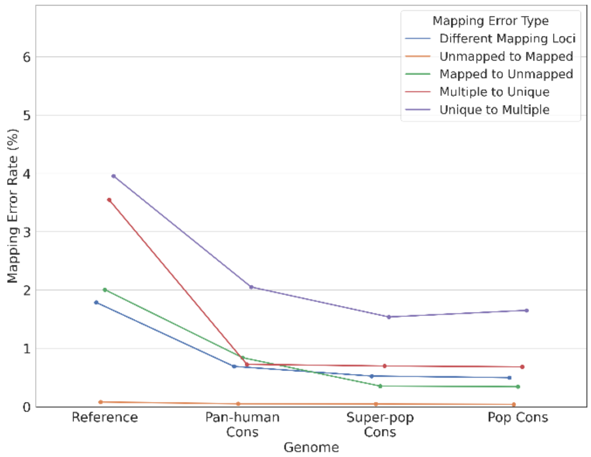

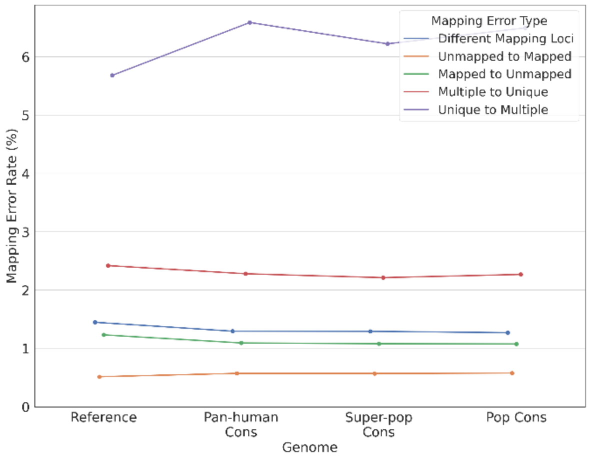

Supplementary Figure S23: Homozygous (left) and heterozygous (right) mapping error rate for each error type for individual HG00731.

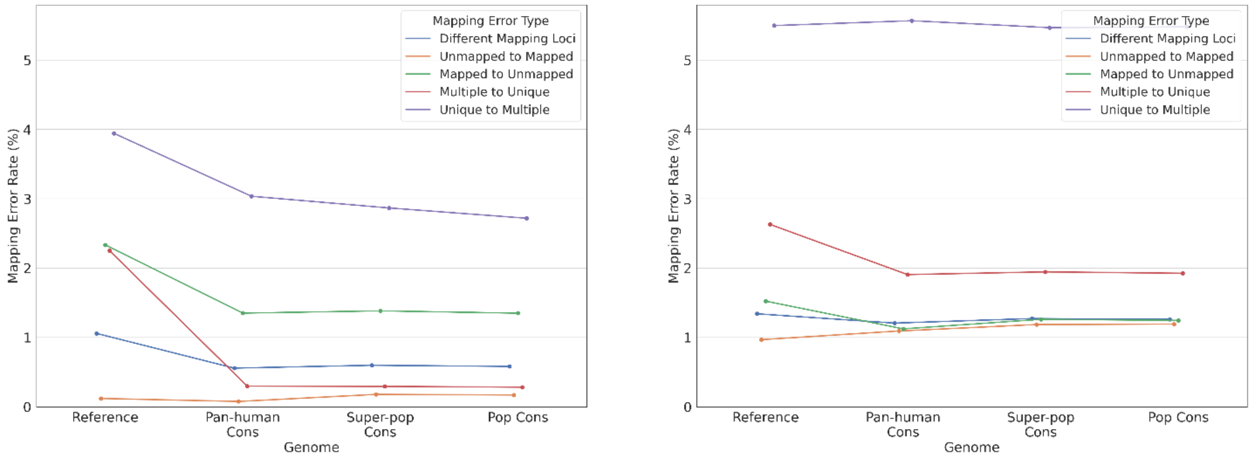

Supplementary Figure S24: Homozygous (left) and heterozygous (right) mapping error rate for each error type for individual HG00732.

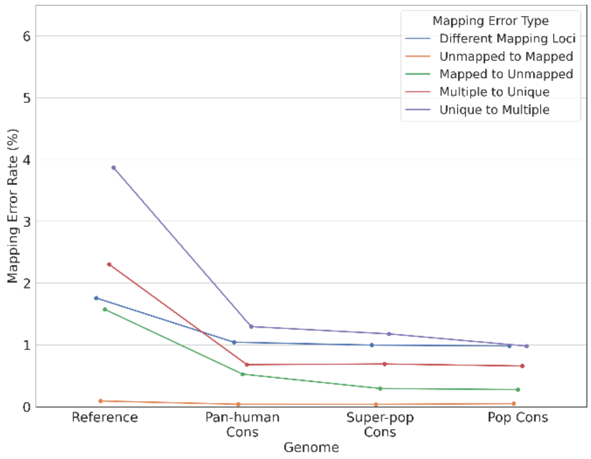

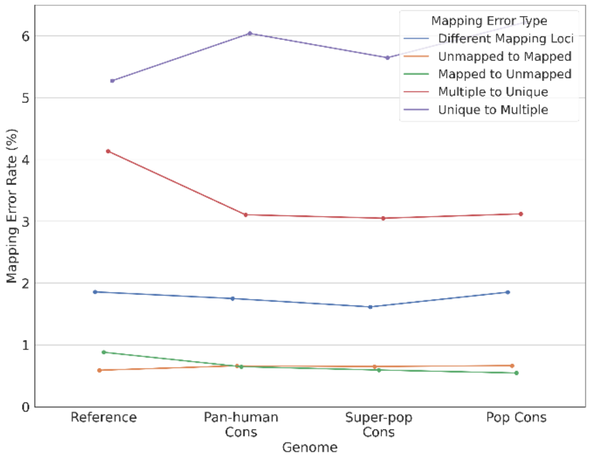

Supplementary Figure S25: Homozygous (left) and heterozygous (right) mapping error rate for each error type for individual HG00733.

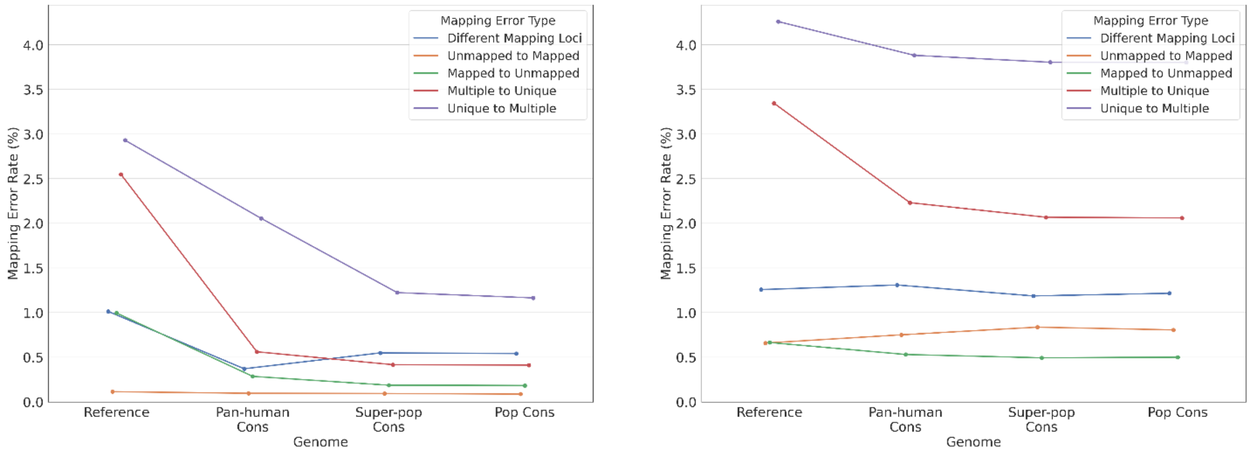

Supplementary Figure S26: Homozygous (left) and heterozygous (right) mapping error rate for each error type for individual NA19238.

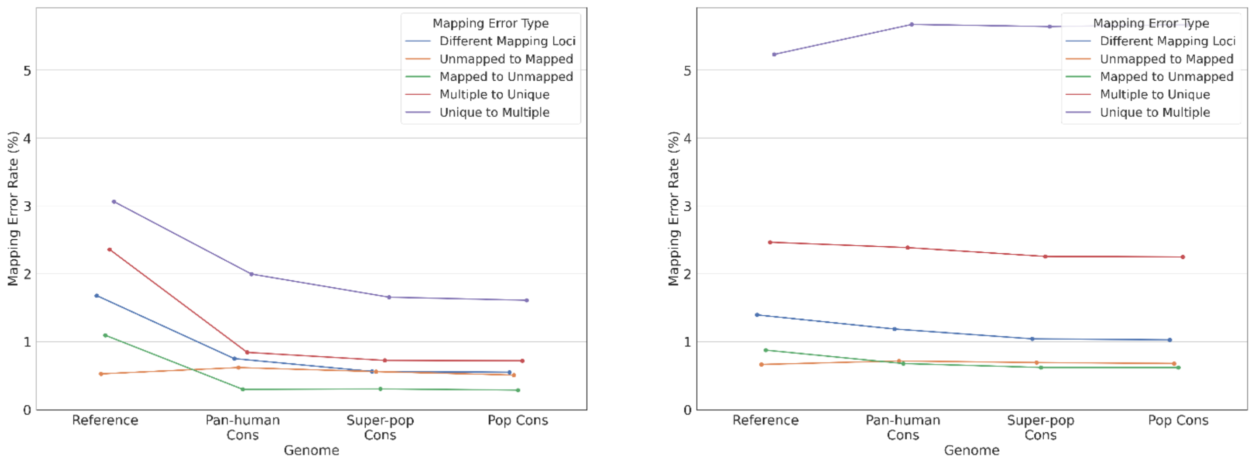

Supplementary Figure S27: Homozygous (left) and heterozygous (right) mapping error rate for each error type for individual NA19239.

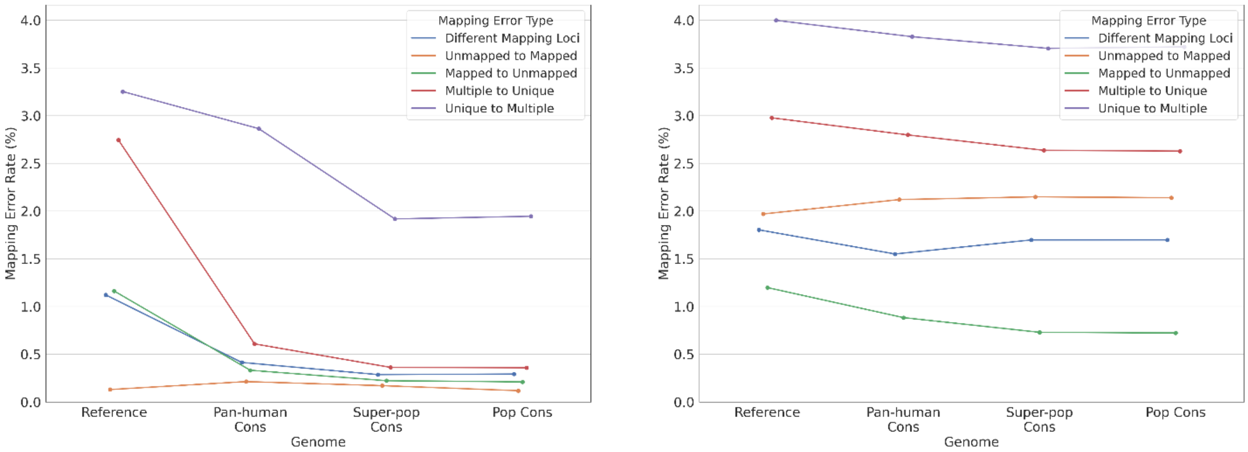

Supplementary Figure S28: Homozygous (left) and heterozygous (right) mapping error rate for each error type for individual NA19240.

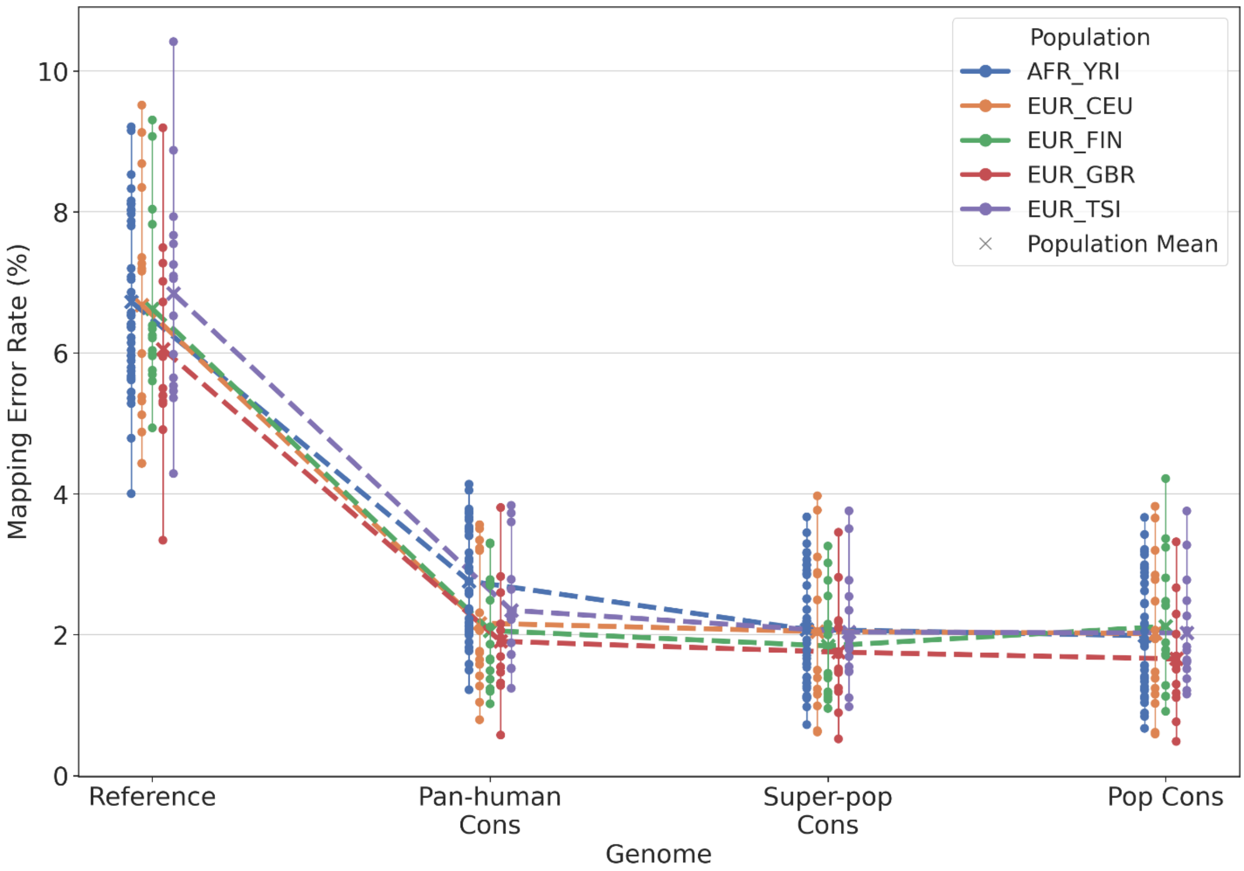

Supplementary Figure S29: Homozygous mapping error rates for individuals sequenced by the Geuvadis consortium. Genome is shown on the x-axis and the mapping error rate is shown on the y-axis. Individuals from the same population are grouped together by color, with each marker representing one individual in the population. The dashed line shows the average error rate for the population and the solid vertical line shows the range of the population.

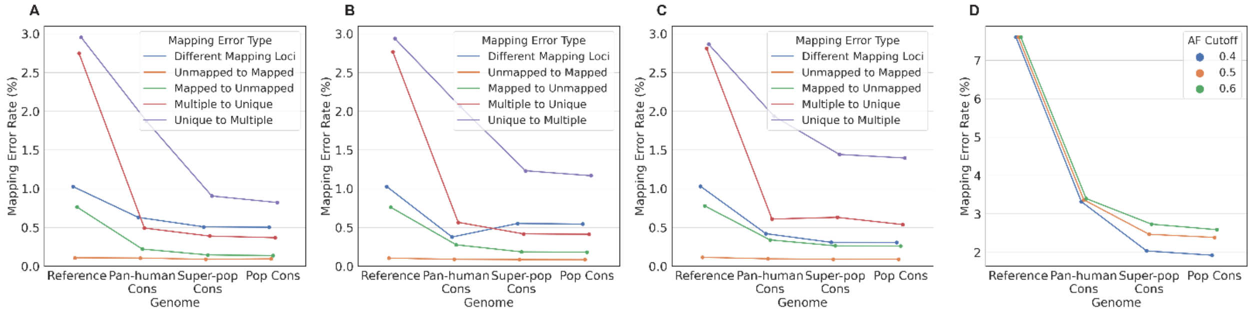

Supplementary Figure S30: Mapping error rates by error type for individual NA19238 for different definitions of major alleles. We used the following allele frequency cutoffs:

A) 40%

B) 50% (the standard)

C) 60%

D) Total mapping error rate for individual NA19238 for different major allele frequency thresholds.

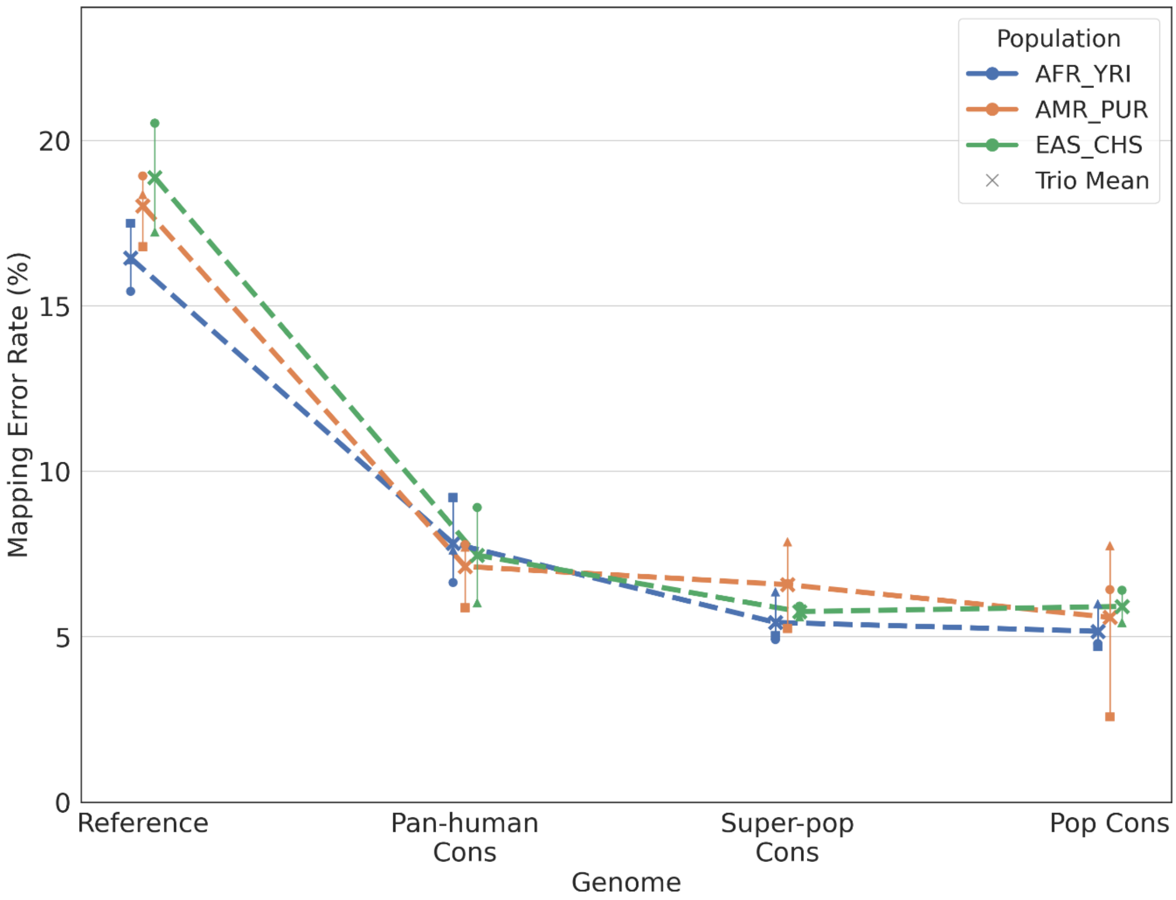

Supplementary Figure S31: Homozygous mapping error rate for all individuals, for reads mapped with the **HISAT2** aligner. Genome is shown on the x-axis and the mapping error rate is shown on the y-axis. Individuals from the same population are grouped together by color, with each marker shape representing one individual in the population. The dashed line shows the average error rate for the population and the solid vertical line shows the range of the population.

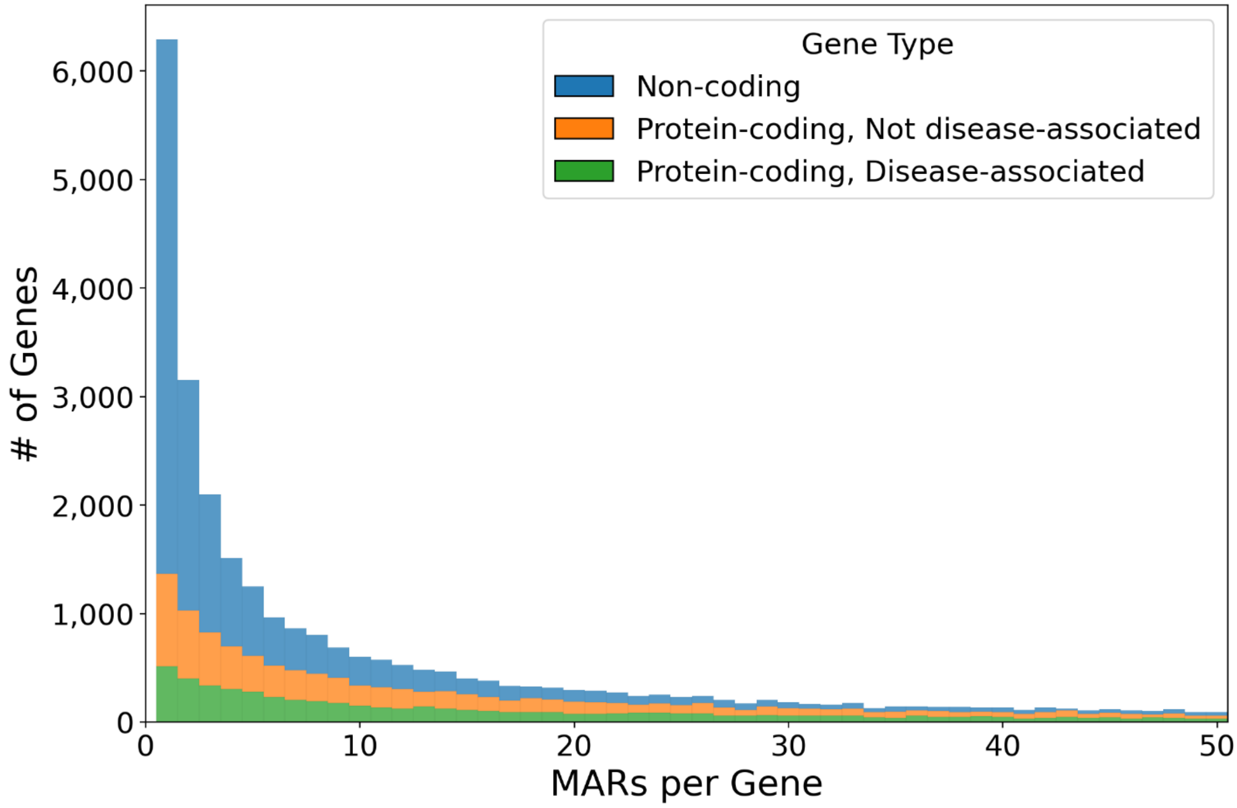

Supplementary Figure S32: Histogram of the number of MARs in exons and introns of the non-coding, protein-coding, and disease-associated genes.

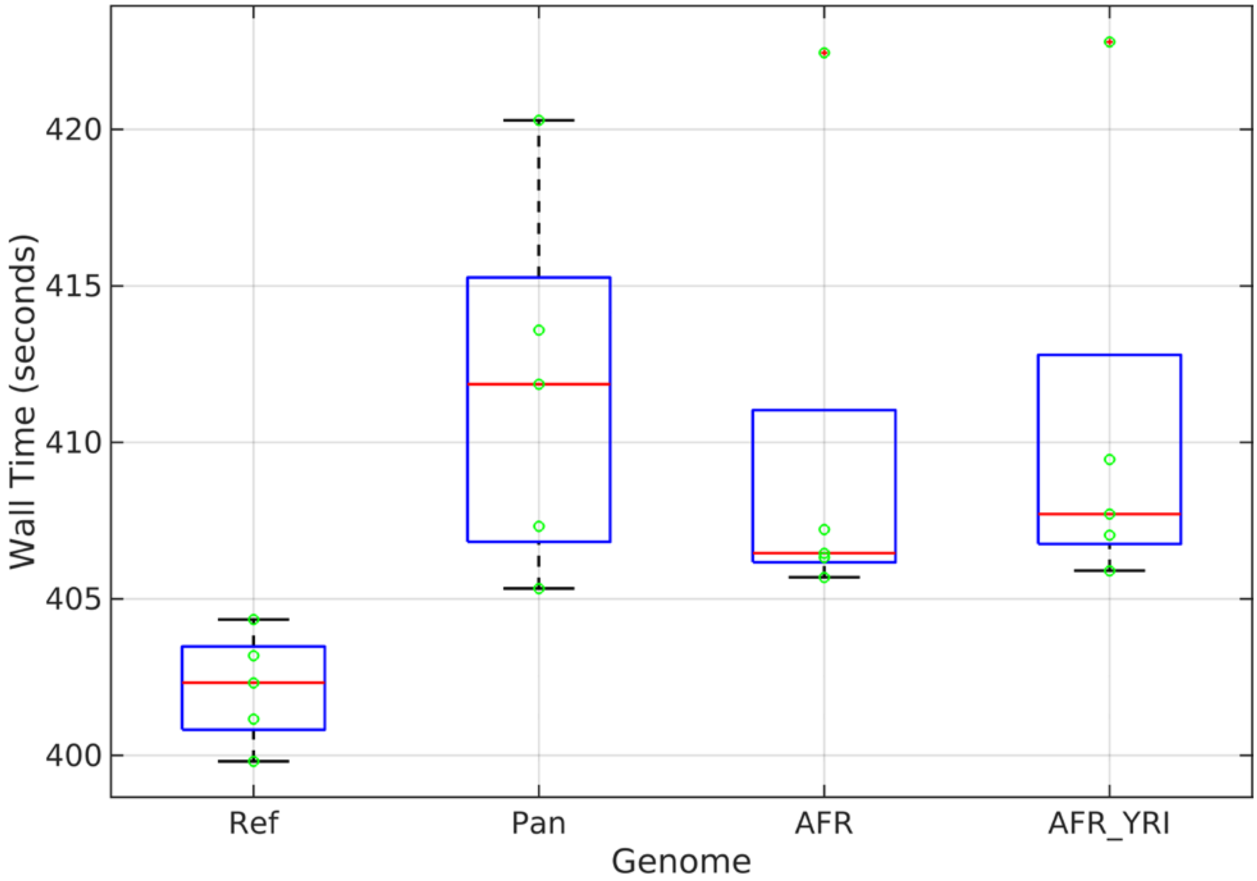

Supplementary Figure S33: Elapsed (wall) time for mapping 64M 2x50b reads from the NA12938 individual to the reference genome, and to the three consensus genomes with coordinate conversion. For each genome, the mapping was repeated 5 times.

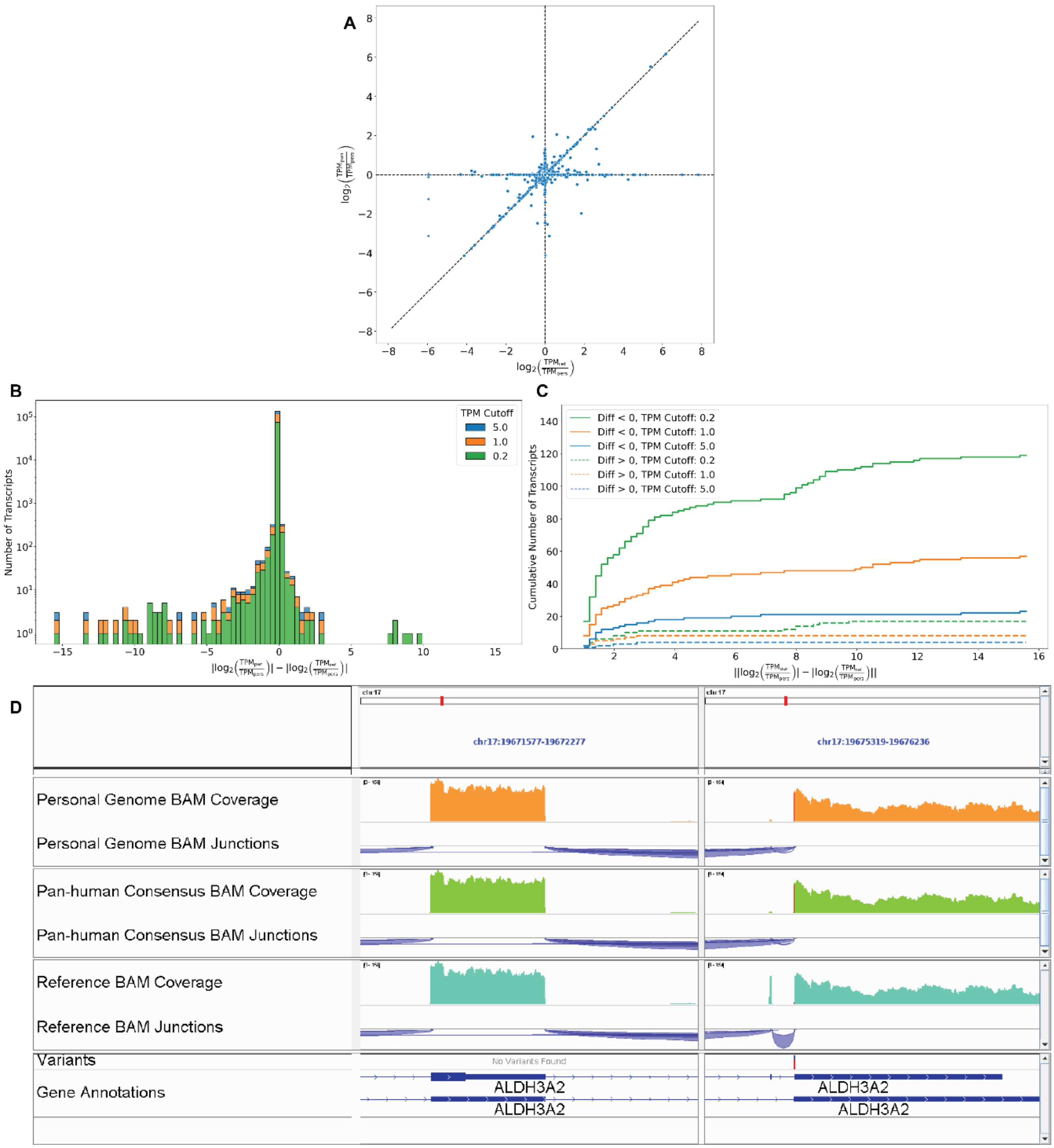

Supplementary Figure S34

(caption on following page)

Supplementary Figure S34: A) Error in transcript quantification in pan-human vs reference genome with respect to the personal genome. Triangles indicate infinite error (i.e. zero expression in one of the genomes).
B) Difference between absolute values of pan-human to personal and reference to personal log-ratios. Different TPM thresholds are represented by different colors.
C) Cumulative distribution of the transcript quantification error. Solid lines represent transcripts which have larger quantification errors in the reference than in the pan-human genome; dashed lines represent the opposite cases. Green, yellow and blue bars correspond to different thresholds (1,10,28) for splice junctions read counts.
D) Read coverage and splice junction tracks for HG00512 reads aligned to the reference, pan-human consensus, and HG00512 personal genome. The regions shown are part of the ALDH3A2 gene. The Variants track shows the location of one MAR that is present in the pan-human consensus and the personal genome.

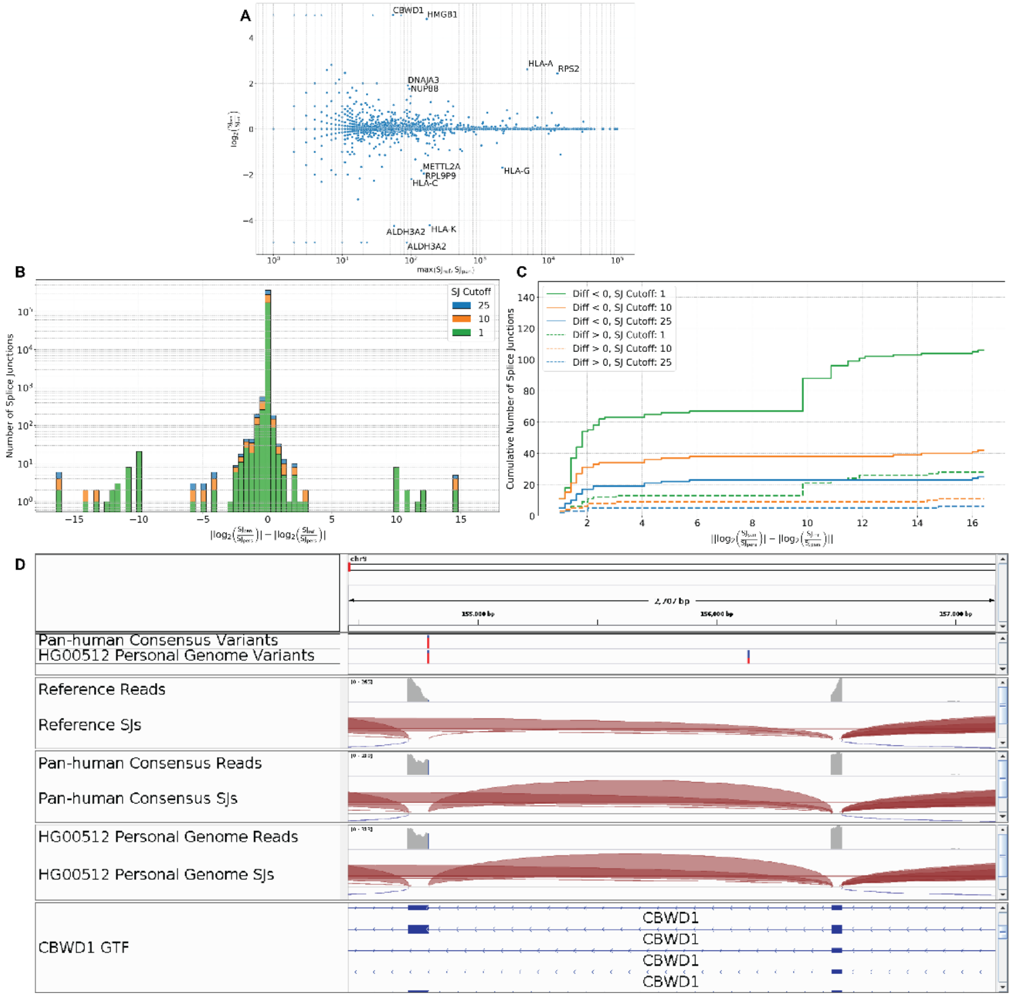

Supplementary Figure S35: A) Log2 fold change between pan-human consensus and reference as a function of the max splice junction expression. Splice junctions with an absolute log fold change > 1.5 and a max expression value > 50 are labeled with the gene in which they fall. Triangles indicate an infinite log2 fold change (i.e. zero expression in one of the genomes).
B) Difference between absolute values of pan-human to personal and reference to personal log-ratios. Different read count thresholds are represented by different colors.
C) Cumulative distribution of the quantification error. Solid lines represent splice junctions which have larger quantification errors in the reference than in the pan-human genome; dashed lines represent the opposite cases.
D) Read coverage and splice junction tracks for HG00512 reads aligned to the reference, pan-human consensus, and HG00512 personal genome. The region shown is part of the CBWD1 gene. The two Variants tracks show the location of a shared MAR that is present in the pan-human consensus and the HG00512 personal genome.

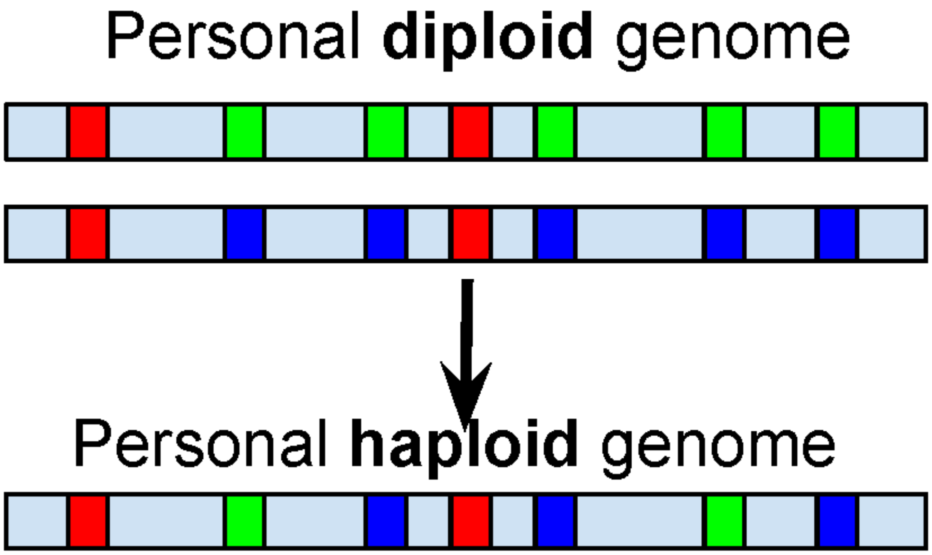

Supplementary Figure S36: Construction of the personal haploid genome from the personal diploid genome. Red blocks represent homozygous variants, which are all incorporated into the haploid genome. Green and blue represent heterozygous alleles from the two haplotypes. For each variant, one of the two alleles is randomly selected.

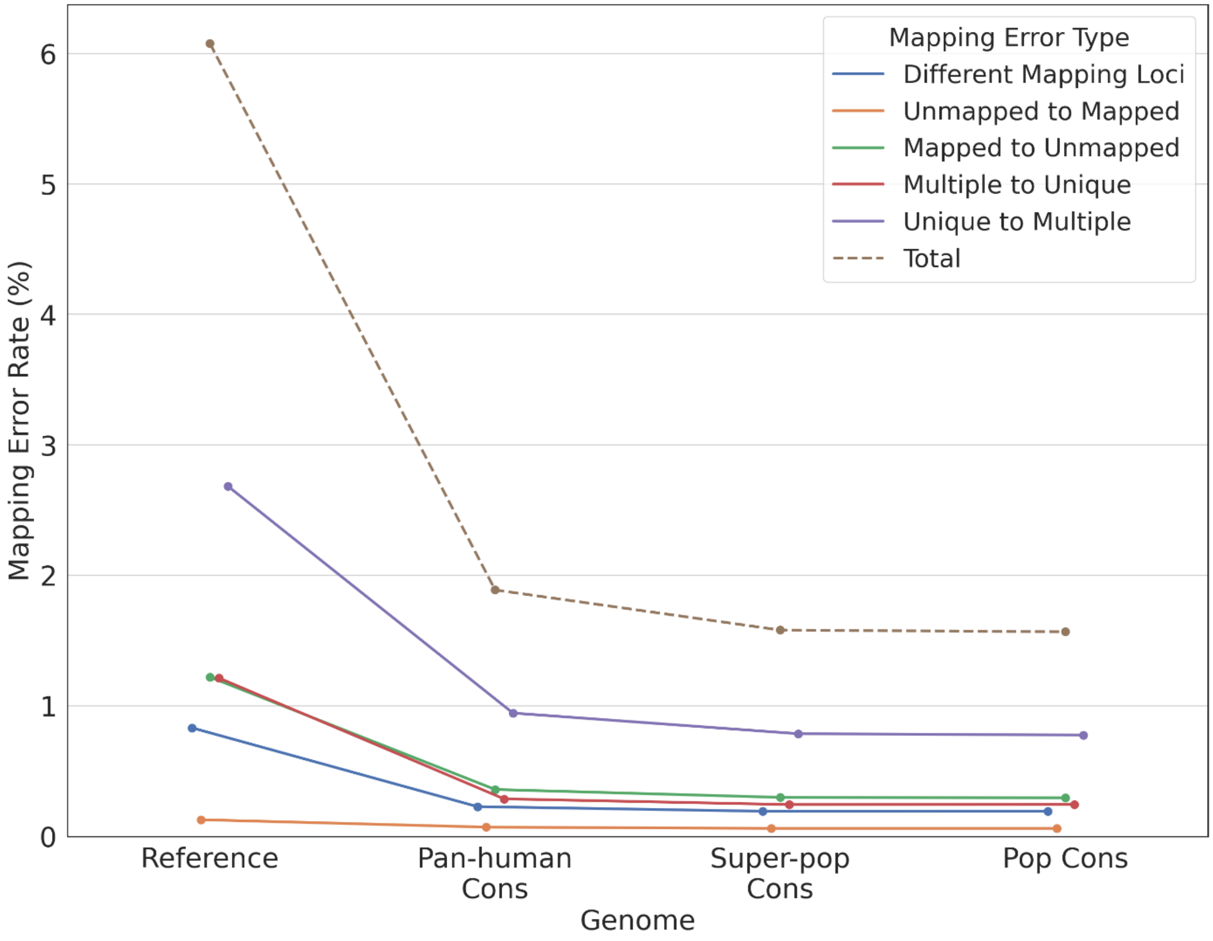

Supplementary Figure S37: Homozygous mapping error rate for the H3K4me3 histone modification ChIP-seq dataset from the ENCODE consortium for the NA12878 individual.
